# Ixcatec ethnoecology: plant management and biocultural heritage in Oaxaca, Mexico

**DOI:** 10.1186/s13002-016-0101-3

**Published:** 2016-07-20

**Authors:** Selene Rangel-Landa, Alejandro Casas, Erandi Rivera-Lozoya, Ignacio Torres-García, Mariana Vallejo-Ramos

**Affiliations:** Instituto de Investigaciones en Ecosistemas y Sustentabilidad, UNAM, Antigua Carretera a Pátzcuaro 8711, Morelia, Michoacán 58190 Mexico; Posgrado en Ciencias Biológicas, UNAM, Ciudad Universitaria Del. Coyoacan, C. P. 04510 México, Mexico; Centro de Investigaciones en Geografía Ambiental, UNAM, Antigua Carretera a Pátzcuaro 8711, Morelia, Michoacán 58190 Mexico

**Keywords:** Biocultural heritage, Domestication, Ethnoecology, Tehuacán-Cuicatlán Valley, Ixcatec, Cultural value, Plant management

## Abstract

**Background:**

Studying motives of plant management allows understanding processes that originated agriculture and current forms of traditional technology innovation. Our work analyses the role of native plants in the Ixcatec subsistence, management practices, native plants biocultural importance, and motivations influencing management decisions. Cultural and ecological importance and management complexity may differ among species according with their use value and availability. We hypothesized that decreasing risk in availability of resources underlies the main motives of management, but curiosity, aesthetic, and ethical values may also be determinant.

**Methods:**

Role of plants in subsistence strategies, forms of use and management was documented through 130 semi-structured interviews and participant observation. Free listing interviews to 38 people were used to estimate the cognitive importance of species used as food, medicine, fuel, fodder, ornament and ceremonial. Species ecological importance was evaluated through sampling vegetation in 22 points. Principal Components Analysis were performed to explore the relation between management, cultural and ecological importance and estimating the biocultural importance of native species.

**Results:**

We recorded 627 useful plant species, 589 of them native. Livelihood strategies of households rely on agriculture, livestock and multiple use of forest resources. At least 400 species are managed, some of them involving artificial selection. Management complexity is the main factor reflecting the biocultural importance of plant species, and the weight of ecological importance and cultural value varied among use types. Management strategies aim to ensure resources availability, to have them closer, to embellish human spaces or satisfying ethical principles.

**Conclusion:**

Decisions about plants management are influenced by perception of risk to satisfy material needs, but immaterial principles are also important. Studying such relation is crucial for understanding past and present technological innovation processes and understand the complex process of developing biocultural legacy.

## Background

In most rural areas of Mexico, especially in those inhabited by indigenous peoples, human subsistence patterns generally involve multiple strategies. Agriculture for direct consumption of products is commonly the main activity, complemented by small scale livestock and the use of numerous forest resources destined to direct consumption and commercialization [1]. These activities occur in territories that are settings of multidimensional and complex interrelationships between humans and nature in socio-ecological systems, integrated as totalities with elements and processes mutually influencing their features and changes [2]. Expressions of these interrelationships are management of wild plant and animal species, domesticated organisms and territories of indigenous and local peoples, which constitute part of the biocultural heritage that are created and maintained through long term by the continuous use and management [3–5]. Management or transformations and decisions made by humans on ecosystems, and on their elements and functions [6], based on TEK are fundamental in the biocultural heritage development process, and constitute a traditional form of facing the uncertainty inherent to complex systems [3, 7–9].

Management may include a broad spectrum of strategies and interactions for appropriation and maintaining natural resources [6, 10, 11]; collective actions to protect them [12], as well as those directed to recover or restore them [6]. These practices (*praxis*) are based on TEK about species and ecosystems (*corpus*) that are in turn strongly linked to beliefs systems (*kosmos*) [7, 13], which have direct influence on resources and ecosystem management.

Plant management is influenced by ecological and social factors [14–17], including the cultural importance of plant species in human life. Some investigations have found positive correlation between cultural and ecological importance, suggesting that most conspicuous plants have more important use values, but numerous examples have been reported contradicting this hypothesis [18, 19]. More informative for constructing ethnobiological theory has been analyzing the complex of the relationships between cultural significance, ecological importance and management complexity. In edible plants, it has been found that species with high cultural value and limited availability are more intensely managed, as a response to the risk in their availability [14–17]. However, humans are not only respondents of critical situations. Curiosity, attraction for beauty, experimentation, innovation, among other intentions are part of human nature and should also be taken into account as factors influencing people’s decision to manage organisms [20–22].

Understanding the role of plant resources with different use types in human subsistence patterns, how management interactions are, and how are these influenced by social and ecological factors, may help to understand the principles of the construction of management techniques, management systems, how processes of domestication are originated, and how processes of current technical innovations are developed, in order to understand the process of construction of the biocultural heritage [6].

The Tehuacán-Cuicatlán Valley in central Mexico, is an important region of the Mexican biocultural heritage [3], harbouring more than 3,000 species of vascular plant species and human cultures with ancestors nearly 10,000 years old [23, 24]. Currently, the Popolocan, Mazatec, Mixtec, Chinantec, Cuicatec, Ixcatec, Chocho, Náhuatl and Mestizo communities make use of nearly 1,750 plant species, at least 610 of them receiving management practices [11, 25]. These figures make the Tehuacán Valley an ideal setting for studying processes influencing decision, innovation and diffusion of experiences on plant management.

This study was performed in Santa María Ixcatlán, the only town where the Ixcatec currently live in the world. It was directed to document subsistence strategies, plants use and management locally practiced, and the main motives to manage them. Also, we examined how cultural, ecological and management factors interact and determine the importance of native plants with different use type on Ixcatec biocultural heritage.

We analyzed the hypothesis that the main motive of managing plants is decreasing the risk that represent their low availability and in some cases to enhance their abundance and quality. Therefore, subsistence is based on multiple activities, diversified management strategies to prevent risks in staple resources availability; and the high cultural importance and management intensity may be associated with low ecological importance. But, attraction for beauty, curiosity and ethical concerns, beyond the satisfaction of primary needs, should also be important aspects in decisions to manage plant resources.

## Methods

### Study area

**Fig. 1:**
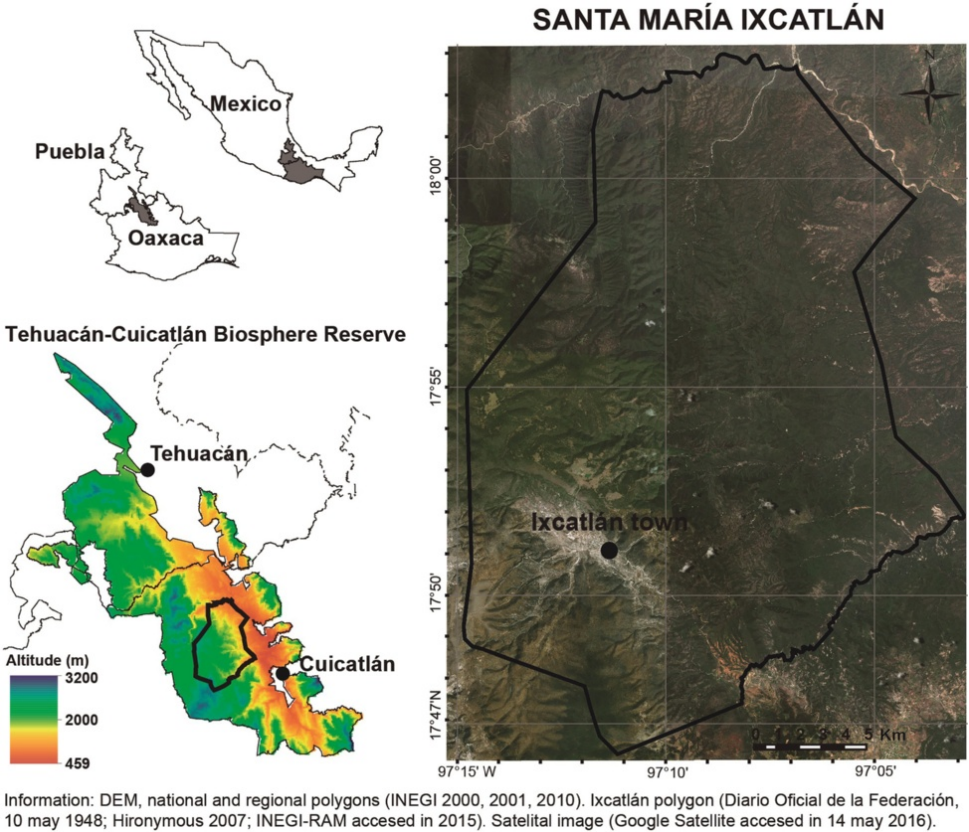
Study area. Location of the the community of Santa María Ixcatlán, Oaxaca, in the Biosphere Reserve Tehuacán-Cuicatlán, central México

At present, the Ixcatec live only in the community of Santa María Ixcatlán, a town governed by the regime of traditional practices and customs. Land tenure is communal with 41,530 ha [26, 27] belonging to the Tehuacán-Cuicatlán Biosphere Reserve, Mexico (Fig. 1). The whole territory is mountainous, with elevations ranging from 800 to 2600 m. Soils in most of the territory derived from calcareous rocks, with thin layers of black organic soils. The town has temperate climate, with annual mean temperature of 17.2 °C, and annual rainfall averaging 721 mm [28, 29]. The rest of the territory has semiarid climate [29]. Vegetation types are oak forests, tropical dry forest, induced grassland and secondary vegetation [30].

In Santa María Ixcatlán live 175 households and 516 people [31]. There is a high migration of young people to the cities of Tehuacán, México, Orizaba, and more recently to the US [32]. Local households’ economy is based on direct consumption of agricultural products, livestock raising and use of forest products [32, 33]. The Communitarian Assembly, conformed by all adult men, is the maximum authority [32], and people obtain rights to have access to resources and lands of the territory through a system of charges and cooperation to communitarian activities [32]. Practically all families are Catholic [32], and have a complex calendar of ceremonies [27, 32, 33]. Nearly a dozen of persons are fluently speakers of Ixcatec, an almost extinct language [34, 35].

### Flora inventory

We conducted ethnoecological studies in Ixcatlán in the period 1999–2001 and in the period 2011–2015 with 16 campaigns of field work. Trial walks accompanied with local informants were carried out to identify vegetation types [36] and collecting botanical voucher specimens throughout the territory of the community. Voucher specimens were deposited at MEXU, EBUM, IE-BAJÍO and IBUG herbaria with Selene Rangel, Erandi Rivera, and Ricardo collection numbers. Nomenclature and classification of species are presented following the APG III classification system consulted in the site www.theplantlist.org [37].

### Interviews

A total of 130 semi-structured interviews to 62 people were conducted to document common names of plants, their use, management practices and motivations to conduct them. Alive plants in their own homegardens, agricultural fields or seen in trial walks, fresh specimens collected a day before, dried specimens and pictures were used as stimulus in these interviews; 22 of the 62 interviewees (9 women and 13 men, with average age of 58.9 years, SD = 22.5) were considered key informants because of their deep knowledge of the territory and plants or because they were Ixcatec speakers. Key informants were selected by the snowball sampling technique, by asking for people with these skills; 15 of them were interviewed from 2 to 11 times in a total of 77 audio or video-recorded sessions, in which on average 17.2 (SD = 23.4) species were reviewed per work session. The other 40 interviewees were considered occasional participants (21 female and 17 male, whose age averaged 53.2 years, SD = 20.8), and they were selected randomly.

More detailed information about informants and activities are included in the Table 6 of Appendix. All interviews used for the analysis showed in this paper were performed in Spanish. All interviews and participant observation data about plant resources use and management were transcribed and systematized into the format of the ethnobotanical data base of Mexico (BADEPLAM) of the Botanical Garden, UNAM. Audio-visual material was stored in the Ixcatec Culture Archive and The Endangered Languages Archive.

### Surveys

Semi-structured surveys with questions on agricultural production and consumption of plant resources were conducted in Spanish between 2000 and 2012 to 21 and 20 households representing the 12 % of the households of Ixcatlán in each year (householders averaging 61.2 years old, SD = 17.2). In 2000 households were selected at random, while in 2012, 24 % of the households surveyed in 2000 were selected, and the rest were selected at random.

### Free listing

In order to identify the plant species with the higher cognitive importance, in 2013 we used the free listing method [38]. We requested in Spanish to 38 people (22 men and 16 women, aging on average 50.6 years, SD = 18.8) to spontaneously listing the names of plants that grow in the territory of Santa María Ixcatlán that are used: 1) as food, 2) to attend illnesses and take care of health, 3) as firewood, 4) to feed livestock, 5) to offer them to Saints, dead people or used in ceremonies, and 6) to embellish the houses and crop land. Once informants stopped listing plants for one use, we asked them to listing plants for other use, and we continued this procedure until finishing the lists of plants for the six uses. Of the 38 people interviewed, 19 were previous informants (13 considered key informants and 6 occasional informants), the other 19 people interviewed were selected at random. Details on the number of lists per use type, the number of items named, the levels of saturation of the datasets, and information about interviewees can be consulted in the Appendix.

### Vegetation sampling

We conducted vegetation samplings in 22 points of nine natural and transformed vegetation types in order to estimate the ecological importance value of species [36]: *Quercus liebmanni* and *Quercus laeta* forest (3 points), *Quercus urbanni* forest (1 point), riparian forest of *Taxodium huegelii* (1 point), *Juniperus flaccida* forest (2 points), izotal of *Beaucarnea stricta* (2 points), mexical (2 points), palm scrubland of *Brahea dulcis* (2 points), grassland (2 points), and agricultural fields (7 points). At each point we established a 500 m^2^ quadrant, where all shrubs and trees were counted and their height and two canopy diameters were measured. Herbs were sampled in five subplots (1 m^2^ each) randomly placed within the area of each 500 m^2^ quadrant. Density and frequency was calculated for each species. Shrubs and trees biomass was calculated through volume formulas of geometric figures [39]. In addition, the floristic composition was sampled in 17 homegardens.

### Data analyses

Livelihood analysis was conducted to assess the subsistence strategies [38], and descriptive data of use and management of plants species were estimated.

Series of Principal Component Analyses (PCA) with native plants species (species with wild populations or Mesoamerican species with naturalized populations in Ixcatlán territory), were performed. Species were considered as operational taxonomic units according to its number of uses, cognitive importance, consumption, ecological importance, complexity of management practices, and management place, all of them aspects involved in the definition of their importance to the biocultural heritage of plant species. The scores of the first principal component obtained in each PCA were considered as biocultural importance index by type of use, since these values are linear combinations that integrating information of the variables, species with positive and highest values were considered more important [15, 40]. The most important variables and how they interact was identified by the correlation values between variables and the first two components [41]. We also identified how species are grouped according with all the variables studied by representing the cloud of species in terms of the two first components [41]. These PCAs were made in JMP 8. statistical software [42].

The cognitive importance was estimated through free listing data with the index of Sutrop (*S*) with the formula S = F/(N mP), where F represents the frequency of the species, N the total number of interviewed people per use category, and mP is the medium position in which the term or species was named [43]. We calculated this index with the software FLAME v1.0 [44]. A zero value was assigned to all species that were not listed by consultants [43]. When an informant said that he/she does not know any plant for a given use or when he/she said that all plants could be used for the requested use, we excluded the list of the analysis.

The consumption of products was estimated as the percentage of households that consumed each plant species throughout the year, based on data documented with surveys conducted in 2012.

The ecological importance of species was estimated through the ecological importance value index EIVI = (Relative frequency + Relative abundance + Relative biomass)/3, calculated by each plant species per sampled site [45]. The floristic composition of homegardens was similarly used to calculate ecological importance.

The complexity of management practices was calculated by the sum of numerical values of management practices. Values were assigned based on the typology proposed by Blancas et al. [11] as follows: a) gathering, simple or planned extraction strategies = 1; b) tolerance or let standing of plants = 2; c) enhancement by promoting abundance of useful plant species or phenotypes = 3; d) protection of desirable plants = 4; e) transplanting entire individuals = 5; f) propagation as seed sowing and vegetative propagation = 6. In addition, we assigned values of 0.5 to simple foraging by domestic animals, and uproot or deliberate removal individuals of the species in question. Values of each practices was summarized per plant species. The places of management were categorized in natural populations plants distribution sites (*in situ* = 1) and sites out of their natural distribution (*ex situ* = 2) [15, 16].

## Results

### Subsistence strategies

**Fig. 2:**
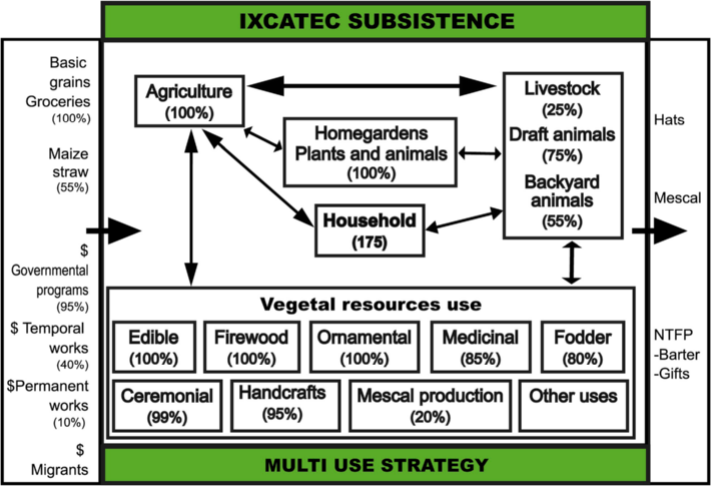
General pattern of multiple use strategy of natural resources for subsistence in the community of Santa María Ixcatlán

**Fig. 3:**
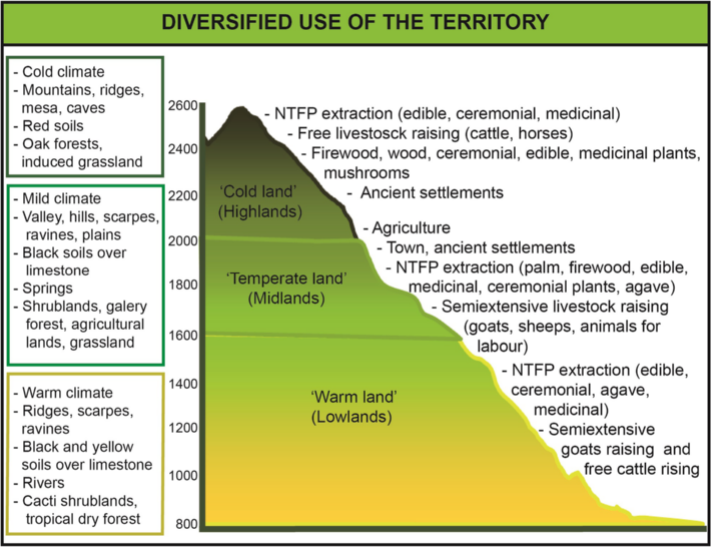
Characteristics of landscapes, general environmental units recognized by people in the territory of Santa María Ixcatlán and plant resources use

**Fig. 4:**
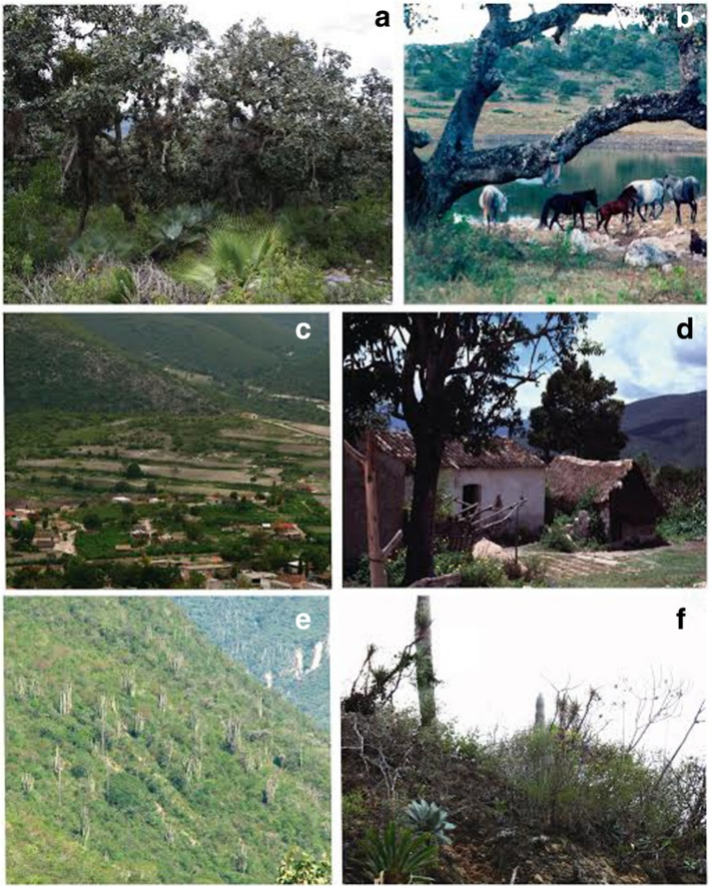
View of environmental units. **a**) *Quercus liebmanni* and *Quercus laeta* forest; **b**) Dam “La Laguna”, grassland and oak forest remnant; **c**) Homegardens, agricultural fields, palm scrublands and mexical in the southwest side of town; **d**) View of a homegarden and a traditional house with roof of palm leaves; **e**) *Pseudomytrocereus fulviceps* shrubland; **f**) *Tillansia grandis* and *Agave potatorum* in *Cephalocereus colummna-trajanni* shrubland

Households are basic units making decisions on economic activities and forest resource management (Fig. 2). Agriculture is the main activity of all households, but maize and beans produced are insufficient to satisfy their annual requirements (Table 1). Multiple-cropping agriculture in the rainy season is carried out in terrains of 1 to 2 ha located around the town (95 % of households), and in homegardens (0.25 to 0.5 ha, managed by 30 % of households) (Figs. 1, 3 and 4). Prayers and rituals drawing or putting crosses made with plants, offering alcoholic beverages to the earth, among other practices, are common during agricultural labours, seed selection and storage, sowing and harvest, as individual farmer or collective petitions for a good rainy season.

**Table 1:** Average and standard deviation of the amounts of maize and beans consumed, produced and productivity (kg/ha) achieved by people of Santa María Ixcatlán, Oaxaca for the periods of the years 1999-2000 and 2011-2012

	Maize		Bean	
	1999–2000	2011–2012	1999–2000	2011–2012
Consumption per year (kg)	766.38 ± 94.34	701.7 ± 73.6	155.6 ± 19.4	112.2 ± 23
Production by household (kg)	285.5 ± 79.9	129.7 ± 62.6	76.2 ± 26.9	48 ± 18.6
Productivity (kg/ha)	289 ± 70.5	82.1 ± 46.7	43.9 ± 10	28.6 ± 9.4
Community deficit (T)	82.7	100	13.7	11.2

Data according to surveys realized to 21 households in 2000 and 20 households in 2012. Values are means and standard errors

All people interviewed referred to difficulties in agriculture, mainly due to a low soil fertility and water scarcity. However, people deal with these problems in homegardens and agricultural fields by adding domestic animals manure, oak forest humus, ash, firewood debris and organic waste; agrochemicals are not used at all. In homegardens, recycling water and spatial arrangement of plants according with their water requirements are common. In agricultural fields, terraces and live fences are common for preventing soil erosion, as well as some dams for the accumulation of soil and moisture (Fig. 4).

Animal husbandry is practiced by almost all households as a saving for emergencies, animal power for agricultural and for gathering activities, only 5 % of households commercialize animals in regional markets (Fig. 2). Nearly 55 % of households raise animals in backyards (1–7 chickens, 1–9 turkeys or 1–4 pigs), 75 % nurture draft animals (1–5 donkeys-mules or 1–4 horses), and 25 % raise livestock (5–80 cows, 10–16 sheeps or 5–70 goats) (Fig. 2). Animals feeding bases on domestic sub-products, maize straw, herbs managed in homegardens and agricultural fields, and foraging in communal lands (Figs. 3 and 4).

Gathering and management of native and introduced plants for direct consumption is practiced by all households (Figs. 2 and 3). Plants provide all the firewood and fodder needed and great part of food, medicines, materials for construction, tools, and other goods. Other important plants are ceremonial and ornamental, which are gathered and managed for direct use or as gifts to relatives (Fig. 2).

Few plant resources or their products are destined to economic interchange, the most important are *Brahea dulcis* and *Agave potatorum* (Fig. 2). The weaving of hats with *Brahea dulcis* leaves is carried out by nearly 84 % of the households, while 10 % are specialized in handcrafting baskets, covers for bottles and other products. Hats are interchanged almost every day for maize, food or money in local stores. From 2011 to 2015 the price of each hat was 0.16 US dollars (based on an interchange rate of $20.00 Mexican pesos by one American dollar), while in 2000 it was $0.12. A household weave on average 28.9 ± 3.65 hats per week, and each hat requires 4.1 young leaves, which means approximately one million of leaves used in the whole community per year. Leaves extraction is carried out mainly in palm scrublands, where *Brahea dulcis* is promoted, protected and tolerated in areas of agricultural fields, but it is widely distributed throughout the whole territory (Figs. 3 and 4). For extracting palm leaves, people cut the young leaves without damaging the apical meristem and avoid gathering leaves during the new moon, otherwise they consider the growth of new leaves can be delayed. Harvesting palm leaves for direct use and local interchange is allowed but sale to regional sellers is forbidden. Palm is considered staple plant as people said “palms are our life because with palm leaves we make hats and we can get all we need to live”.

Approximately 20 % of households prepare mescal with *Agave potatorum* once to 10 times per year (4.8 ± 1.49) (Fig. 2). For 2012 we estimated that the whole community produced 192 mescal batches, using 91.14 ± 9.78 agaves per batch, in total nearly 17,500 agaves per year, whereas for the year 2000 we estimated the use of 4,900 individuals. The price of one litre of mescal was $2.5 US dollars in 2000 and from $6 to $9 in 2011 to 2015. Although *Agave potatorum* is widely distributed in temperate and warm parts of the territory of the community (Figs. 3 and 4), the mescal producers said that they have to go progressively farer to extract agaves and they even complement their needs buying agaves to neighbouring communities; sometimes they complement their batches with the wild *Agave vivipara* extracted in the warm land of the territory. Agave extraction is allowed for all community members; however, the relation between mescal producers and communal authorities has become tense in the last years, since federal environmental authorities are trying to regulate this activity in the region. Since 2011 some mescal producers started to enhance the availability of agaves near their houses or agricultural fields by spreading seeds or cultivating them in homegardens and green houses. Some mescal producers have participated in exchanges of experiences for agave management with other communities, and governmental programs have promoted some actions as reforestations and the construction of a communitarian greenhouse that stared to produce agave plants in 2015.

The activities described are supported by using different environments and sites of the territory (Figs. 3 and 4). The whole territory is of common use, but knowledge about distribution, abundance and quality of plant resources are recognized as basic issues to access to any locality and its resources. The subsistence strategy is complemented by economic subsidies from governmental programs for elderly, child scholarships, creole seeds conservation, and agriculture and stockbreeding development (Fig. 2). In 2000 assistance program started to support the 45 % of households, by 2012 nearly 95 % of the households received monetary incomes from those programs. In almost a half of the households at least one member has temporal or occasional employments at town that allow them to get additional monetary incomes (Fig. 2). Although irregularly, some migrants support their families to pay communal fees for celebrations, maintaining religious monuments and building public infrastructure (Fig. 2).

### Plants use

We inventoried 780 vascular plants species belonging to 119 botanical families; 589 of them are native to Ixcatlán, and the other 191 have been introduced from other parts of Mexico and the world (Appendix). In order to satisfy their broad spectrum of needs people make use of 627 plants species with one to 27 use categories (Table 2), 267 species have one use and 360 have between 2 and 11 different use types.

**Table 2:** Use categories of Santa María Ixcatlán plant species. Data according to 62 people interviewed in 130 work sessions

Use	Native	Introduced	Total
Fodder	238	30	268
Ornamental	160	110	270^a^
Medicinal	166	53	219
Edible	72	66	138
Ceremonial	73	55	128
Firewood	44	2	46
Utensils	29	4	33
Living fences	24	6	30
Timber products and construction	27	2	29
Shade	12	11	23
Food aditive (flavor)	9	6	15
Handcrafts	11	1	11
Insects repellent	8	0	8
Soil control	6	2	8
Animals medicine	1	1	6
Facilitator^b^	3	2	5
Toys	5	5	5
Alcoholic beverages	2	1	3
Cosmetic	2	1	3
Soap	2	1	3
Paint	3	0	3
Weather predictors	2	0	2
Aromatizing	1	0	1
Tannin source	1	0	1
Water attracter	1	0	1
Glues	1	0	1
Poisons	1	0	1
Unknown	150	3	153
TOTAL	589	191	780

a = 132 species are considered “luxury of houses”, 80 as “luxury of the mountain”, and 59 as “luxury of houses and mountain”; b = Plants used as stake, hosts and nurse plant

#### Fodder

A total of 268 plant species are consumed by domestic animals (Table 2, Appendix). 238 species being native to Ixcatlán and 165 of them have other uses mainly as edible, medicinal or as ornamental plants. Of the 30 introduced species 15 are propagated, and some of them are highly valued (Appendix). *Zea mays* is the most valuable specie as fodder, its stubble is used by the 80 % of households and during periods of scarcity, 87 % of the households have to buy it to regional sellers (Fig. 2, Appendix). Other important introduced plants are *Avena fatua* and *Hordeum vulgare* which are cultivated specifically for this use.

#### Ornamental

Ixcatlán people name as “luxury” (‘lujo’ in Spanish) the plant species that embellish or adornment houses, homegardens, agricultural fields and landscapes, in the two last cases these plants are called “mountain luxury”. High variation was documented about which plants are considered as luxury, as most consultants said “it is something that depends on the appreciation of beauty of things by each person”. People consider that luxury plants embellish the house, calls friendship, invites people to come into the house, allows to strength the heart or spirit and it is motive of proud for the owner. The importance of maintaining these plants varies among people, but generally are appreciated because in addition to the quality of embellish, these plants provide shade, good sites for resting and well-being or are used as fodder, edible and medicine. Nearly 270 species were recognized for its quality of embellish, 160 of them are native to Ixcatlán, 37 of them are not used in other form. 19 luxury plant species are transplanted from forest to houses or are propagated through sexual or asexual propagules. Introduced plants are highly valued (Table 2, Appendix), and are common gift of outsiders that visit the town, or these are obtained through governmental programs or by interchanging palm leaves with outside sellers.

#### Medicinal

We documented 219 species used as medicine (Table 2), 61 of them exclusively used with this purpose, the rest have other uses mainly fodder, edible or are considered as “luxury plants". The medicinal plants commonly are used to treat stomach-ache, cold, fever, ear pain, sprains, and cultural illnesses like “sustos” (shocks caused by impressions), “aires” (malaise caused by uncomfortable situations) and “alferecia” (weakness, loss of appetite and irritability in children). Although knowledge about plants used in childbirth is extensive, few young women recognize to use them. In 2000, all people said to use medicinal plants, but in 2012, 15 % of people interviewed said they only use allopathic therapies and the rest said to combine traditional and institutional medicine. Of the 53 introduced species some are highly valued for their medicinal use (Table 2, Appendix) and are cultivated to have them available as it is the cases of *Matricaria chamomilla*, *Tanacetum parthenium* and *Artemisia ludoviciana.*

#### Edible

We documented 138 plant species used as food, 99 of them have other uses, mainly as fodder, medicinal and ornamental (Appendix, Table 2). Nearly 50 species complement the diet of people which is based on maize tortillas, beans and chili sauces; 66 introduced edible species are cultivated, as it is the cases of maize, beans, vegetables, condiments and fruits (Appendix). These plants are available in the local stores but people say “the little that we harvest is a saving, these plants are things that we do not have to buy”. Other reasons for cultivating are quality; people argued that vegetables locally produced are of better quality than others from outside particularly *Coriandrum sativum* and *Solanum lycopersicum*, they consider that local products have better taste, smell and texture.

#### Ceremonial

A total of 128 plant species are used to offer them to Catholic Saints in altars at homes, hermitages, thumbs, and the church. Some are used in ceremonies and processions (Table 2, Appendix); 117of them have other uses, 95 are used as ornamental or luxury (Table 2). The introduced plants are highly appreciated (Appendix), and particularly cultivated for their flowers, like *Tagetes erecta* used by 95 % of households during the great feast of the Day of the Dead (Appendix). People recognize several varieties according to the size, colour and form of flowers, and it is common to store seeds of their favourite variants to be propagated in the next cycle. Local interchange of ceremonial plants flowers is common among households as gifts or trade, especially of introduced species as *Tagetes erecta*, *Zantedeschia aethiopica*, *Leucanthemum maximum*, between others.

#### Firewood

We recorded 48 species used as firewood (Table 2), 44 of them are native species, and 46 have other uses. These are the main source of cooking energy (only 35 % of households have gas stoves, but all use firewood for cook “maize tortillas”), and is the unique fuel to mescal production and for baking bread. In the year 2000, consumption of firewood per household was of 143.4 ± 11.3 kg/week, and in 2012 it was 108.8 ± 12 kg/week, a decrease apparently due to a governmental program for installing efficient stoves. For mescal production the consumption increased from 16.2 ton in 2000 to 63.36 ton in 2012; nearly 52 % of these quantities is from alive oaks, which is considered the appropriate wood for baking the agave stems in the process of mescal production.

### Plant management

Nearly 82 % of all plants species recorded (636 spp.) are recognized to be under interventions by humans or foraged by domestic animals (Appendix); 424 of them are managed through at least two different practice types and 401 species are under practices directed to maintain or increase their availability.

Gathering is the most common practice for obtaining products of native plants and it is the only practice for 83 species (Table 3). This practice was documented among wild and introduced species, some of which have become naturalized (Appendix). We recorded 251 native and introduced species having special protection (Table 3). In homegardens and agricultural fields protection comprises actions like irrigation, exclusion from herbivorous and competitors, nursing, adding of livestock manure, protection against frost, weeding, pruning, and providing or removing shade. In communal lands, protection of native plants is conducted by avoiding pastoral routes in sites where people know valuable plants occur. Also, the Communitarian Assembly construct regulations for protecting some species, based on principles of favoring direct consumption by local people, forbidding extraction for commercialization and cutting of alive trees. However these regulations as practices directed to prevent unnecessary damage not always are followed.

**Table 3:** Plants management practices realized substitute "realized" by "carried out" in Santa María Ixcatlán

Management practice	Native	Introduced	Total
Gathering	281	18	299
Foraging	223	20	243
Tolerance	152	54	206
Protection	91	160	251
Trasplanting	71	68	139
Uproot	63	13	76
Propagation	33	122	155
Enhancement	9	25	34
Unknown	143	1	144

Data according to 62 people interviewed in 130 work sessions

In total, 206 species are tolerated during clearing vegetation in homegardens and agricultural fields. The main reason is its utility, but 23 species that are not used are tolerated since people said that “plants could be useful in the future”, and “do not interfere with the development of other plants” or because “plants have the right to live” and “are part of nature”.

Propagation of 155 species is carried out by seeds, bulbs, corms, rhizomes, tubers, pseudo-bulbs, bulbils, plantlets, shoots, cladodes and sticks; 33 of them are native wild species used mainly as ornamental. Complete individuals of 139 species are transplanted, 71 of them from wild populations in forests to homegardens and agricultural fields. Occasionally, some epiphytic bromeliads and orchid species are relocated from one branch or tree to other, when their host’s branches are cutting to allow their survival.

The abundance of 26 species or some variants is promoted by tolerating them until seed production, and in some cases seeds are collected, stored and then sown or dispersed; 76 species (63 of them native) are constantly uprooted in agricultural fields and homegardens (Table 3), some of them are also under practices to maintain them and ensure their availability.

### Biocultural importance

#### Fodder

**Fig. 5:**
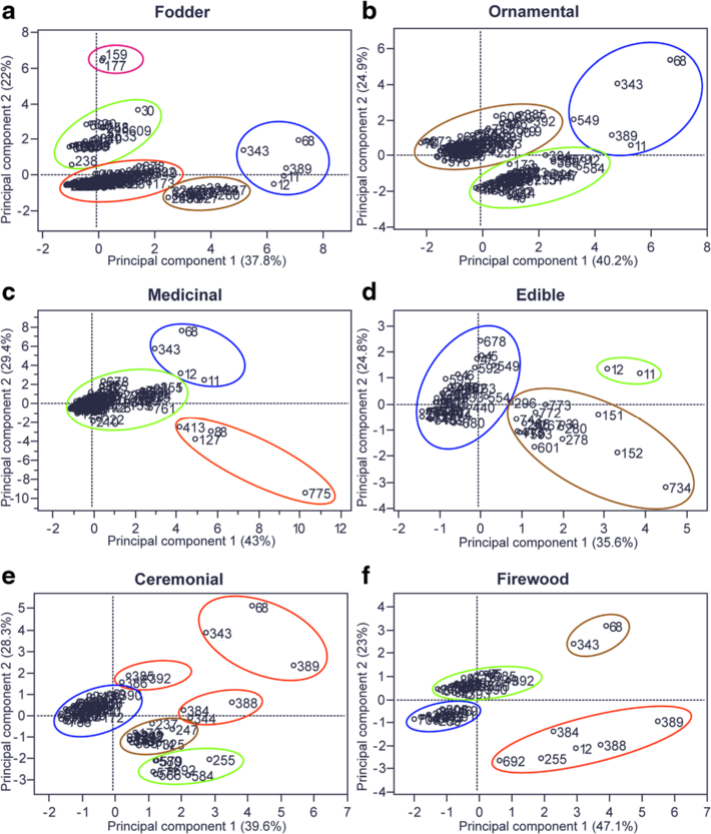
Spatial arrangement of species used as **a**) edible, **b**) medicinal, **c**) firewood, **d**) fodder, **e**) ceremonial and **f**) ornamental, according to the Principal Component Analysis PCA performed with cultural, ecological and management variables. 11 = *Agave potatorum*, 12 *= Agave salmiana* subsp. *tehuacanensis*, 30 = *Amaranthus hybridus*, 68 = *Brahea dulcis*, 88 = *Ageratina mairetiana*, 127 = *Grindelia inuloides*, 151 = *Porophyllum linaria*, 152 = *Porophyllum ruderale*, 159 *= Simsia lagascaeformis*, 177 = *Tithonia tubaeformis*, 255 = *Bursera biflora*, 238 = *Hechtia oaxacana*,237 = *Catopsis compacta,* 247 = *Tillandsia usneoides,* 325 = *Sedum dendroideum*, 278 = *Opuntia lasiacantha*, 296 = *Dysphania ambrosioides*, 343 = *Juniperus flaccida*, 384 = *Quercus acutifolia*, 388 = *Quercus laeta*, 389 = *Quercus liebmannii*, 392 = *Quercus urbanii*, 413 = *Clinopodium mexicanum*, 533 = *Anoda cristata*, 549 = *Morus celtidifolia*, 554 = *Dasylirion serratifolium*, 579 = *Laelia albida*, 580 = *Laelia anceps*, 584 = *Euchile karwinskii*, 601 = *Peperomia quadrifolia*, 682 = *Lindleya mespiliodes*, 692 = *Chiococca alba*, 722 = *Lamourouxia dasyantha,* 734 = *Capsicum annuum*, 743 = *Physalis philadelphica*, 761 = *Turnera difussa*, 775 = *Lippia oaxacana*. For all species identity see ID correspondence on Table 5 of Appendix

Variation in biocultural importance of 238 fodder native species is mainly explained by management type and number of uses (38 % of variation in the first principal component), and cognitive prominence and consumption (22 % of variation in the second principal component; Table 4). Species with the highest biocultural importance (blue circle in Fig. 5a) are subject to several management practices, but its use as fodder is low with the exception of *Quercus liebmani* whose acorns are gathered and stored for feeding pigs, and inflorescences of *Agave* spp. that are occasionally consumed by cattle. *Simsia lagascaeformis* and *Tithonia tubaeformis* (pink circle in Fig. 5a) are the species with highest cognitive value, and are tolerated in homegardens or agricultural fields, where these are also uprooted to control their abundance. Similar situation occurs with *Amaranthus hybridus, Mirabilis xalapa, Sicyos laciniatus* and grass species (green circle in Fig. 5a).

**Table 4:** Contribution of socio-ecological factors to explain the variation of native plant species biocultural importance

Use type	Fodder		Ornamental		Medicinal		Edible		Ceremonial		Firewood	
Factor	PC1	PC2	PC1	PC2	PC1	PC2	PC1	PC2	PC1	PC2	PC1	PC2
Cognitive importance	−0.09	**0.78**	0.55	0.31	0.72	**−0.58**	0.44	−0.18	0.54	0.24	0.69	−0.17
Consump-tion	0.04	**0.77**	0.55	0.12	0.63	**−0.64**	0.39	−0.32	0.35	**−0.63**	0.33	**0.67**
Number of uses	**0.76**	0.16	**0.74**	0.47	0.52	**0.69**	0.47	**0.73**	**0.65**	**0.61**	0.75	0.29
Ecological importance	0.48	0.21	0.53	**0.52**	0.31	**0.65**	0.32	**0.82**	0.51	**0.68**	0.61	0.57
Management complexity	**0.93**	−0.01	**0.81**	**−0.52**	**0.82**	0.33	**0.93**	−0.13	**0.89**	−0.29	**0.9**	−0.24
Management site	**0.76**	−0.22	0.59	**−0.76**	**0.8**	−0.01	**0.78**	−0.36	**0.69**	**−0.58**	0.69	**−0.66**

Data are correlation values between variables and the first two components of Principal Components Analysis PCAs. Values in bold have high influence in principal components, therefore in the classification of biocultural importance too

Legumes, oak acorns, herb species and grasses are the main fodder for cattle, goats and sheep. Management practices to ensure their availability are poor or absent (orange and brown circles in Fig. 5a). *Tillandsia gymnobotrya* and *Hechtia oaxacana* are highly valued as fodder, substituting maize stubble (green circle in Fig. 5a). Shepherds drop the epiphytic plants for cattle and goats, and nearly 30 % of households gather and carry them to town for feeding donkeys and horses, extracting 800 to 1920 individuals per year.

#### Ornamental plants

Biocultural importance of 160 native ornamental plants is explained mainly by their management complexity and number of uses (40 % of the variation explained by the first principal component), and ecological importance and management (25 % of variation explained by the second principal component) (Table 4). The most important plant species (*Brahea dulcis*, *Juniperus flaccida*, *Quercus liebmanni*, *Morus celtidifolia* and *Agave potatorum*), with exception of *Morus celtidifolia* are considered “luxury of the mountain”, all of them are highly valued because of their multiple uses, and have high ecological importance (blue circle in Fig. 5b).

Oaks, grasses and numerous plant species producing beautiful flowers are appreciated to embellish the wilderness and some of them are maintained for this appraisal on agricultural fields or protected against livestock, as it is the case of the terrestrial orchids (*Cyrtopodium macrobulbon* and *Govenia lagenophora*), among others (brown circle in Fig. 5b).

Some valuable “luxury of the mountain” plants, are carried to homegardens; for instance, *Euchile karwinskii,* several spherical and barrel cacti species (*Mammillaria* spp.*, Coryphantha retusa,* and *Ferocactus* spp.), Crassulaceae species, *Tillandsia* spp., among others. These plants are propagated and maintained for embellishing the house and 42 species are used for ceremonial purposes too (green circle in Fig. 5b).

#### Medicinal plants

The biocultural importance of the 166 native medicinal plant species is explained mainly by their complexity and site of management, and their cognitive prominence in the first principal component (43 % of variation). Number of uses, ecological importance, consumption and cognitive importance are important in the second principal component (29 % of variation) (Table 4). In general, native plants with the highest biocultural importance like *Lippia oaxacana*, *Ageratina mairetiana*, *Grindelia inuloides* and *Clinopodium mexicanum* have few uses, high cognitive prominence and low ecological importance (orange circle in Fig. 5c). These plants are mainly gathered and stored to ensure their availability when it could be necessary. Some people have propagated these plants but said that “they are experimenting” but “quality of plants growing in nature is better than the cultivated ones”.

There is another group of plants like *Agave* spp., *Juniperus flaccida* and *Brahea dulcis*, which have high ecological importance, are subject to complex management and used with numerous purposes, and occasionally used as medicine (blue circle in Fig. 5c). The rest of the species (green circle in Fig. 5c) are occasionally consumed, collected when they are needed, and some of them are also valued for other types of use.

#### Edible

Principal components analysis shows that biocultural importance of the 72 native plants is explained mainly by management practices complexity and management site (*ex situ* or *in situ*) in the first principal component (36 % of variation), and ecological importance and number of uses in the second principal component (25 % of variation) (Table 4). Native plants with higher biocultural importance are those with greater management complexity, consumed by more families and have few uses, regardless of their ecological importance (brown circle in Fig. 5d).

One of the most important plant species is *Capsicum annuum*, consumed by all households, mainly getting it by interchange, but it is also cultivated in homegardens but the wild variety is rarely gathered. Species like *Porophyllum ruderale*, *Porophyllum linaria*, *Amaranthus hybridus*, *Opuntia lasiacantha,* and *Dysphania ambrosiodes* are consumed by nearly all households and their contribution to diet is greatly important. For instance, the green *Amaranthus hybridus* is consumed on average 14.4 ± 2.4 times per year from June to September, almost always together with *Porophyllum linaria*; *Dysphania ambrosiodes* is cooked with beans and consumed every day by all households. These species are subject to management in agricultural fields and cultivated in homegardens to ensure their availability and to have them close and in case of scarcity are getting in the stores. *Physalis philadelphica* is consumed in sauces almost always raw to allow its seeds to germinate after dispersed when washing dishes in homegardens, where plants of this species are tolerated, transplanted and protected.

Other species are obtained by gathering (blue circle in Fig. 5d). Some of the most valuable (e. g. *Dasylirion serratifolium* and *Peperomia cuadrifolia*) are consumed by nearly all households and commonly are shared with relatives, especially elders who are unable to get them by themselves. Some people have tried to propagate them in homegardens but they said that their experiments were unsuccessful because they obtain low production, it was difficult to maintain them, and require long time to harvest their products. *Agave* species are grouped (green circle in Fig. 5d), have high biocultural values, are intensely managed, abundant and highly valued for multiple purposes, although the consumption of its flowers as food is currently uncommon.

#### Ceremonial plants

Variation in biocultural importance of the 73 native species is mainly explained by management complexity and number of uses in the first principal component (40 % of variation), ecological importance, consumption and number of uses (28 % of variation explained by the second principal component; Table 4). The species with the highest biocultural value were those more intensely managed and valued for other uses (orange circles in Fig. 5e), for instance oaks that are part of the game of “El palo” played in the celebration of the Day of the Dead, when teams of young men go to the forest to cut whole dead trees and carry them on to the town to be fired in front of the church. Other examples are *Brahea dulcis* leaves, which are used to weave shoes for deceased people and *Juniperus flaccida* whose resin is used when *Bursera* resin is scarce or unavailable.

The most cognitively salient species are appreciated for their flowers smell and beauty (green circle in Fig. 5e), which receive management practices and are extensively used regardless of their low ecological importance*.* In the extraction of orchid flowers people take care of leaving some bulbs, and after their ceremonial use, their bulbils are transplanted in homegradens as it occurs in the case of *Euchile karwinskii. Laelia albida* is cultivated in 65 % of homegardens and *Laelia anceps* in 35 % of them, this management is motivated by the appreciation of their beauty and scarcity in forests. Resin of *Bursera biflora* is particularly appreciated and used in a high number of rituals, this tree species is protected *in situ,* cannot be tamed or even damaged for extracting its resin and most people use only the resin of those trees naturally injured by insects located in warm lands to assure the resin quality (Fig. 3). Other species like *Chiococca alba, Rhynchostele maculata* and *Epidendrum radioferens* are highly valued and frequently used species but rarely transplanted into homegardens, in part because people consider they are abundant, but in part because of the difficulties for their propagation. Some species are used to embellish the “Nativity scenes” (*Mammilaria* spp., *Catopsis compacta*, *Tillandsia* spp.) are transplanted in homegardens after their use (brown circle in Fig. 5e). Most of ceremonial species are only gathered as it is the case of *Lamourouxia dasyantha* (blue circle in Fig. 5e) and in many cases are shared with relatives, especially old people*.*

#### Firewood

Principal components analysis shows that biocultural importance of plants used as firewood is mainly explained by the complexity of their management in the first principal component (47 % of variation), and consumption and ecological importance in the second component (23 % of variation) (Table 4). Species used as firewood with the highest biocultural importance are oaks *Quercus* spp. (orange circle in Fig. 5f), which are consumed by all households, and have the highest cognitive prominence. Oaks are tolerated and protected in agricultural fields, and sometimes people transplanted and take care of them in their houses as ornamental plants. In this group, *Agave salmiana* subsp. *tehuacanensis* is valued as good firewood, but its use is uncommon since people prefer to use its dry stalk for house construction. Two important species used as firewood are *Brahea dulcis* and *Juniperus flaccida*, which are intensely managed in agricultural fields and homegardens, have high ecological importance, are frequently used, and are highly culturally valued because of their multiple uses (brown circle in Fig. 5f).

The remaining species receive poor management (green and blue circles in Fig. 5f) and differ in their consumption, cognitive prominence and ecological importance. Some of these species have high biocultural value (*Quercus urbanii*, *Quercus castanea*, *Quercus conspersa*, *Rhus chondroloma*, *Rhus standleyi*, and *Morus celtidifolia*; green circle in Fig. 5f).

Although of the most valuable species for all interviewees are *Quercus* spp., *Arbutus xalapensis and Juniperus flaccida*, the “charges” (measurement unit which is the amount of material that a donkey is able to carry) composition highly varied among households, oaks being on average $\left(\overline{\mathrm{X}}=79\kern0.5em \%\right)$, the rest are at least 30 species of shrubs managed in agricultural fields and homegardens being *Dodonaea viscosa*, *Acacia* spp., *Comarostaphylis polyfolia*, *Eysenhardtia polystachya*, and *Garrya ovata*, among the most common species.

## Discussion and conclusions

### Subsistence strategy

The multiple use of resources that including a great variety of ecosystems and resources and characterizing the Ixcatec subsistence are expressions of common patterns of interactions between humans and plants found among indigenous peoples of Mesoamerica [1, 3, 39, 46–49]. Such pattern is particularly important in a region like the Tehuacan Valley where the scarcity and uncertainty of rainfall and agricultural yield are also characteristic [17, 33, 39, 50]. Interchange of natural resources in the regional markets for obtaining staple food and other goods is clearly a strategy to face problems of availability of resources since pre-Columbian times [51]. For instance, commercialization and barter of local products like palm leaves, hats, mescal, and domestic animals, is a common strategy in numerous Mesoamerican communities [52–54] and many rural regions in the world to deal with the uncertainty [55].

Other activities like commerce and income subsidized by governmental programs, are part of the process of adaptation that may contribute to face eventual environmental and social adversities, similarly as recently documented among Mayan communities in southern Mexico [53]. The assistance support programmes from Government are progressively more important in the local subsistence strategies, but also, these programmes represent risks for the systems of management of natural resources, as it has been documented for programmes supporting agriculture, which promote the removal of trees and shrubs in agricultural land, thus affecting the maintenance of agroforestry systems [8, 21]. Seasonal employments allow solving some problems [17], but also these may cause the regardless or abandonment of traditional activities, the loss of TEK and, in some cases, the abandonment of the community.

### Management diversity

The widely management practices set and other cultural and social strategies documented have allowed to maintain plant species that sustain the multiuse subsistence strategies as it has been reported at regional level [11, 56, 57]

At regional level, gathering and foraging of plant resources by humans and their domestic animals are the most common and simple form of interaction between social and ecological systems [56], but for most useful species recorded people carry out practices directed to maintain and ensure their future availability [11], and a broad variety of strategies are being carried out for such a purpose [17]. These general trends were observed in Santa María Ixcatlán, is practiced in an even higher percentage of plant species (nearly 65 %), which is an expression of the particularly deep of TEK developed by the Ixcatec.

Management practices such as tolerance, enhancing, protection and cultivation (by sowing, planting or transplanting) look for ensuring availability of plant resources and controlling its uncertainty, are primary mechanism in the domestication process for some species [10, 58]. It has allowed through selection of particular individual (phenotypes) and germplasm to start cultivation, maintaining and continuing processes of domestication. These processes were evident in the staple crops, as well as in wild and semi-domesticated *Physalis philadelphica*, *Tagetes erecta* and *Cosmos bipinnatus* in which selection to satisfy particular flavours, colours, and size, among others characteristics is carried out by people.

The socio-cultural strategies documented in all types of use as it is the mobility in resource gathering of valuable species, the diversification of resources to satisfy a need, and the substitution of one species with another or with other materials, have been recognized as buffer mechanisms to uncertainty [17, 59]. Other important strategies based on social interactions as was the interchange of plants as gifts and interchange of information about management techniques, allow important diffusion of experiences among households and communities and are important mechanisms of social cohesion, an important issue to maintain traditional institutions [17, 60]. Strategies associated to governance as it is the case of regulations are being effective for conserving some species. This is for instance the case of *Litsea glaucescens* and several oak species *Quercus* spp., whose populations are conserved in Ixcatlán through local regulations that only allow the extraction for direct consumption by househlds, but in other villages of the region have been severely affected and became extinct [15, 16]. However, in other species regulations have been ineffective for controlling new intensities of extraction required because of socio-economic needs. This is clearly the case of *Agave potatorum* in which the increasing demand of mescal has been for the moment higher than the capacity for collective regulations and technical responses.

Other interactions like removal (uprooting), opposed to maintenance, shows the complexity of interactions between humans and plants and the importance of detailed knowledge that people may have to take into account to make a decision based in the balance of the negative effects and utility that these species could provide [15]. For instance, in some cases like *Thitonia tubaeiformis, Amaranthus hybridus* and other weeds, which are valuable plants, people control its abundance inside of the agricultural field at begging the cycle in order to prevent competition with maize, but at the same time protect them in the borders to prevent fodder scarcity just in case that maize straw become scarce or to ensure the availability of greens.

The management practices have involved the transformation of ecosystems through intentional or incidental changes in the composition and structure of vegetation, the modification of relief, hydrological systems and biogeochemical processes in soils [61]. Concrete examples of this process are the creation and maintaining of secondary vegetation as induced grasslands and palm scrubland, changes in vegetation structure in forest zones where grazing routes are, erosive process in current and abandoned agricultural fields, and engineering works to retain soil and water for agriculture and livestock (Figs. 1, 2 and 3). Homegardens, crop fields and pasturelands distributed in the three types of environments recognized by the Ixcatec within their territory (Fig. 3), have originated a great variety of landscape units where management of wild and domesticated plant species take place, conforming forest, agroforestry, agro-silvo pastoral, and silvo-pastoral systems [62, 63]. In these systems people maintain a high level of biodiversity; for instance, on average people of Ixcatlán maintain 29 woody native species in their agricultural plots [22]. These systems are biocultural expressions and areas continually generating new biocultural diversity through also continual observation and experimenting management techniques [8, 64]. In the palm scrublands, for instance, which are highly important for the Ixcatec, people have shaped their conformation managing *Brahea dulcis* in order to increase its availability in agricultural and fallow plots, as well as in homegardens. This practice has happened most probably since pre-Columbian times, since this species is important for Ixcatec people [51, 52].

The role of plant species in the Ixcatec subsistence and in the interactions of humans to conserve plant resources may define particularities of their own culture [3, 65]. Management of some plant species is closely related with the form of preparation of food stoves, as it was described for *Physalis philadelphica*. Relation of the Ixcatec with the palm *Brahea dulcis* is particularly significant, this species is part of almost all activities in their daily life, and it has been considered as an indissoluble element of Ixcatec culture [32, 33, 51, 52, 66, 67].

The high levels of diversity and interactions documented in Ixcatlán compared with the regional flora (30 % of the total regional flora, 36 % of all useful plants recorded in the region, and 66 % of managed species identified in the Tehuacán Valley) [11, 24, 25], confirm the importance of the Ixcatec biocultural heritage and the character of the Tehuacan Valley as a priority biocultural region of Mexico [3].

Our research and sampling effort is one of the highest carried out by ethnoecological studies in the Tehuacán Valley [11, 15–17, 56, 68–71]. This fact confirms that it is still needed continuing efforts to documenting TEK, biocultural processes of diversification and their connection with management innovation and domestication. In this region, archaeological records in caves has been source of information about biocultural construction since prehistory, whereas local studies should continue documenting one of the areas with highest richness of ethnobotanical knowledge of Mexico and a place where ongoing processes for sustainable resource management and local processes of domestication are taking place.

### Biocultural importance

The integration of socio-cultural and ecological variables for understanding the importance of plant species, follows the proposal by Castaneda and Stepp [72] for estimating ethnoecological importance. Our evaluation found that variables associated to management complexity are in general those more contributing to explain the variation in the first principal component of the six use categories analysed. This fact suggests that management is representative of the socio-ecological factors interacting and mutually influencing their properties [73]. In other words, studying management of natural resources is a good methodological basis for understanding socio-ecological systems and construction of biocultural heritage.

*Brahea dulcis*, *Juniperus flaccida*, and *Agave salmiana* subsp. *tehuacanensis* have particularly high biocultural importance values in almost all use types analysed. This fact is because of their multipurpose use, their cultural and ecological importance and their intensive management. The positive relation between cultural and ecological importance might be explained through the hypothesis of ecological appearance [18, 74, 75], but we rather propose that the ecological importance currently observed is in part a result of ancient ecosystem management directed to increase their availability. The high resistance to disturbance, reproductive capacity of these species, among other ecological factors have favoured the enhancing of their abundance.

The relation between ecological and cultural importance varied in the different use types analysed. Among plants used as ceremonial and medicinal, the species with higher cognitive prominence and consumption have low availability, and their management is mainly through socio-cultural strategies, directed to ensure their availability, as the harvest technics to ensure their survival after the harvest, but not necessarily are directed to increase their abundance.

The number of uses was an important factor in edible, medicinal, fodder, ceremonial and ornamental plants; however, among medicinal plants, the species with higher cognitive prominence were those with few uses, in other words their properties determining them specialized medicinal plants, which is apparently related with their quality as resource [76].

Highly cognitive valued species not always are the most consumed or managed. For instance, species highly valued as ceremonial, like orchids have a low consumption because the difficulty to obtain them or be manipulated to increase their availability. These results and those found by several authors studying factors influencing management of edible plants [15, 77, 78], indicate that management motives may be variable not only related with cultural importance and scarcity, which suggests the importance of continuing research in this line.

## Conclusion

### Management factors and motives

A case that allows observing how people dynamically construct processes of decision making about management is *Agave potatorum*, in which the perception of risk of disappearing of the resource is the main factor detonating management actions, as documented for other plant resources of the Tehuacán Valley [15]. The strategies developed depend on TEK of both species and ecosystems [17], but there are external factors influencing experimenting innovation in management actions, as illustrated in the cases of several species of *Agave* [40, 79], in which markets have influenced increasing of extraction and pressures on agave populations and new management techniques [16, 17, 40]. This case illustrates that crises detonate innovation, activating processes of experimenting, monitoring, adapting, testing and interchanging local and external experiences, as well as enhancing processes of social organization, collaboration with governmental and academic sectors, learning and adaptation, in which the communitarian platforms of dialogue are crucial for facing risks and uncertainty [80, 81].

In other cases, the uncertainty in the availability of highly valued resources are motives for managing other species with redundant use and are able to substitute particular desirable resources, as are the cases of *Tithonia tubaeformis* and *Simsia lagascaeformis* whose abundance is promoted in controlled ways before the uncertainty of the main fodder of the study area (maize stubble). Such a complex decision making has important consequences in households’ economy [82] and biodiversity conservation in agroforestry systems [21, 22, 83].

Uncertainty operates associated to several factors, and ensuring the products quality is another management motive. People prefer consuming their own crops, which are considered of better quality over those commercialized in stores. Practices to assure the quality not are exclusively on crop plants, others like *Bursera biflora* have specialized resin extraction techniques that take advantage of natural processes assuring the resin quality avoiding injure the trees, instead of cutting trunks, a common practice in other localities [84]. Moreover, the perception of quality loss discourages *ex situ* management, in addition to energy investment and difficulties involved in maintaining these species outside their environments, as was noted in *Bursera biflora* and medicinal plants.

The aesthetical sense, expressed by people that consider that plants embellish the spaces where they occur, as Cook noted [33] in mid 20th century, appears to be an important motive that determining the permanence of numerous native species in homegardens and crop fields as forests conservation. This motive has been reported by other authors in agroforestry systems of the region [21, 22], and our study suggests its high importance because of the high number of species considered as house or “mountain luxury”, which receive some type of management practices.

Ethical principles like the fact that people recognize that plants are living beings with a right to exist, that plants should not be damaged because of whim, are ethical principles that motive management practices as tolerance. Also the including of several species in belief systems and matching cycles of plant management with the rituals calendar, suggest that although the Ixcatec *kosmos* is permeated by Catholic thinking, it maintains features with other Mesoamerican views of the world reported by other authors [20, 85].

Curiosity was mentioned to be involved in all management practices in response to motives such as uncertainty in plant resources’ availability or aesthetical needs. It enhances testing new techniques or new species or be persistent when reproductive requirements make difficult the plants propagation.

Deepen the study of motivations and socio-economic and cultural factors that influence plant management allow understanding the processes of decision making construction and biocultural legacy. Such studies could provide unique opportunities for strengthening conservation strategies of sustainable forms of management of resources and ecosystems.

## Abbreviations

TEK, traditional ecological knowledge; UNAM, Universidad Nacional Autónoma de México; USA, United States of America

## Figures and Tables

**Fig. 6:**
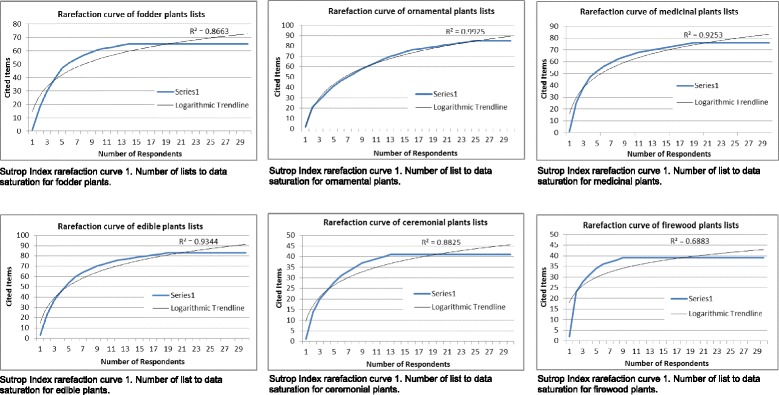
Rarefaction curves of the Sutrop Index S

## Appendix

Plant species of Santa María Ixcatlán. Species, number of uses, management, socio-cultural and ecological aspects; rarefaction curves of S Index, Ixcatec participants details, and botanical experts.

**Table 5:** Species, Spanish common names, number of uses, percentage of families that consume it; cognitive prominence values expressed as S = Sutrop relative prominence index^2^ and biocultural importance expressed as first component value of the principal component analysis by use type (edible, medicinal, firewood, fodder, ceremonial and ornamental; distribution on vegetal types, importance ecological index value (EIVI); specie origin region, ecological status, management practices and management site with respect to species wild populations

ID	Family	Specie	Voucher number ^a^	Common name	Number of uses	Consumption by use (Households %)	Fodder		Ornamental		Medicinal		Edible		Ceremonial		Firewood		Vegetation type^h^	EIVI (ecological importance value index)	Origin^i^	Ecological status^j^	Management practices^k^	Management site^l^
							Sutrop Index value^b^	PC value	Sutrop Index value^c^	PC value	Sutrop Index value^d^	PC1 value	Sutrop Index value^e^	PC1 value	Sutrop Index value^f^	PC value	Sutrop Index value^g^	PC value						
1	Acanthaceae	*Carlowrightia neesiana* (Schauer ex Nees) T.F.Daniel	SRL-1385				0		0		0		0		0		0		SB	0	Ixc	W		
2	Acanthaceae	*Justicia candicans* (Nees) L.D.Benson	SRL-1395				0		0		0		0		0		0		CaCe	0	Ixc	W		
3	Acanthaceae	*Justicia gonzalezii* (Greenm.) Henr. & Hiriart	SRL-1333, SRL-1362				0		0		0		0		0		0		CaCe	0	Ixc	W		
4	Acanthaceae	*Justicia spicigera* Schltdl	SRL-92, SRL-188, ERL-41, ERL-58, ERL-216, ERL-224	Tintonil	1		0		0		0.0101		0		0		0		Sol	0.000153	TCV	W	P, Prp	*ex situ*
5	Acanthaceae	*Ruellia lactea* Cav.	Photo record				0		0		0		0		0		0		BG, Pal	0	Ixc	W	T	*in situ*
20	Aizoaceae	*Aptenia cordifolia* (L.f.) Schwantes	ERL-46		1	Ornamental = 6	0		0		0		0		0		0		Sol	0.000026	EAAA	W	P, Prp	*ex situ*
21	Aizoaceae	*Carpobrotus* sp.	Photo record		1	Ornamental = 6	0		0		0		0		0		0		Sol	0.000026	EAAA	W	P, Prp	*ex situ*
22	Aizoaceae	*Mesembryanthemum* sp.	ERL-213		1	Ornamental = 6	0		0		0		0		0		0		Sol	0.000026	EAAA	W	P, Prp	*ex situ*
28	Alstromeriaceae	*Bomarea hirtella* (Kunth) Herb.	RLF-290				0		0		0		0		0		0		Iz	0	Ixc	W		
29	Amaranthaceae	*Alternanthera caracasana* Kunth	ERL-21, SRL-93	Maravilla	2		0	-0.216	0		0	-0.5049	0		0		0		Bal, Sol	0.000153	Ixc	R-W	F, G, T, Ur	*in situ*
30	Amaranthaceae	*Amaranthus hybridus* L.	SRL-79, SRL-80, SRL-1122, SRL-1141, ERL-74, ERL-102	Quelite tintonil	3	Fodder = 60, edible = 95	0.0207	1.4999	0		0	0.6125	0.2516	2.025	0		0		Bal, Sol, TS	0.006548	Ixc	R-W	E, F, G, P, T, Ur	*in situ*
31	Amaranthaceae	*Beta vulgaris* L.	Photo record	Betabel, acelga	1		0		0		0		0.0218		0		0		Sol	0.000051	EAAA	D	P, Prp	*ex situ*
33	Amaranthaceae	*Celosia argentea* L.	Photo record	Moco de pavo	2	Ornamental = 18, ceremonial = 30	0		0		0		0		0.0296		0		Sol	0.000077	TCV	D	E, P, Prp	*ex situ*
34	Amaranthaceae	*Gomphrena serrata* L.	RLF-60, RLF-242, SRL-90, SRL-378, SRL-1175	Gallitos	2		0	-0.3826	0		0	-0.6606	0		0		0		Bal, BEA, BN, Iz, Me, Palm	0.008464	Ixc	W	F, G	*in situ*
35	Amaranthaceae	*Iresine schaffneri* S.Watson	RLF-320		1		0	-1.0555	0		0		0		0		0		Iz	0.000784	Ixc	W	F	*in situ*
36	Amaranthaceae	*Iresine* sp.	SRL-1488				0		0		0		0		0		0		Me	0	Ixc	W		
26	Amaryllidaceae	*Agapanthus africanus* (L.) Hoffmanns.	Photo record	Pando morado	2	Ornamental = 18	0		0.0016		0		0		0		0		Pal, Sol	0.000077	EAAA	D	P, Prp	*ex situ*
23	Amaryllidaceae	*Allium cepa* L.	ERL-177	Cebolla	1	Edible = 100	0		0		0		0		0		0		Sol	0.000051	EAAA	D	P, Prp	*ex situ*
24	Amaryllidaceae	*Allium sativum* L.	Photo record	Ajo	2	Edible = 100	0		0		0.0016		0		0		0		Sol	0.000153	EAAA	D	P, Prp	*ex situ*
37	Amaryllidaceae	*Crinum × powellii* Hort.	ERL-237	Azucena blanca	2	Ornamental = 35, ceremonial = 11	0		0		0		0		0.0588		0		Pal, Sol	0.000153	EAAA	D	P, Prp	*ex situ*
38	Amaryllidaceae	*Hippeastrum puniceum* (Lam.) Voss Herb.	Photo record	Azucena roja	2	Ornamental = 29, ceremonial = 11	0		0.0168		0		0		0.0056		0		Pal, Sol	0.000128	AC	D	P, Prp	*ex situ*
39	Amaryllidaceae	*Hymenocallis harrisiana* Herb.	Photo record		1		0		0.0042	-1.4444	0		0		0		0		Pal	0	Ixc	W	T	*in situ*
40	Amaryllidaceae	*Sprekelia formosissima* (L.) Herb.	Photo record	Azucena roja	1	Ornamental = 6	0		0.0078	1.0545	0		0		0		0		Me, Sol	0.000026	Ixc	W	E, P, Prp, Ti	*ex situ, in situ*
41	Amaryllidaceae	*Zephyranthes minuta* (Kunth) D.Dietr.Herb.	Photo record		1		0		0.0037	-2.0057	0		0		0		0		Me	0	Ixc	W		
42	Anacardiaceae	*Actinocheita potentillifolia* (Turcz.) Bullock	RLF-109, RLF-274, SRL-1183, SRL-1368	Tetlate	2	Firewood = 100	0		0		0	-0.1037	0		0		0.0092	-0.5723	CaCe, Me, Iz, Palm	0.003085	Ixc	W	G, Prp	*in situ*
43	Anacardiaceae	*Cyrtocarpa procera* Kunth	SRL-1358	Chupandio	1	Edible = 20	0		0		0		0	-1.3811	0		0		CaCe	0	Ixc	W	G	*in situ*
44	Anacardiaceae	*Pistacia mexicana* Kunth	RLF-326, SRL-1211, SRL-1340, SRL-1523	Socoya	7		0.0161	1.24	0		0.0069	0.4921	0		0		0		BG, CaCe, Iz, SB, Pal, Sol	0.000026	Ixc	W	F, G, T	*in situ*
45	Anacardiaceae	*Rhus chondroloma* Standl.	RLF-282, SRL-1222, SRL-1460	Zumaque	7	Firewood = 100	0	1.6290	0.0147	0.9307	0.002	0.6407	0	0.06	0		0.03	0.2684	BEA, BEC, Me, Pal, SB, TS	0.013869	Ixc	W	F, G, T	*in situ*
46	Anacardiaceae	*Rhus standleyi* F.A.Barkley	RLF-59, RLF-255, SRL-269, SRL-472, SRL-1248, SRL-1470	Encino chaparro, zomaque grueso	3	Firewood = 100	0		0		0	-0.043	0	-0.5044	0		0.03	-0.422	BEA, BEC, Iz, Me, Pal, Palm, Sol, TS	0.023686	Ixc	W	G, T	*in situ*
47	Anacardiaceae	*Rhus virens* Lindl. ex A.Gray	RLF-58, RLF-219, SRL-275, SRL-468, SRL-1218	Zumaque	6	Firewood = 100	0	1.4214	0.0147	0.7703	0.002	0.5159	0	-0.0476	0		0.03	0.134	BEA, BN, Iz, Me, BB , TS	0.017724	Ixc	W	F, G, T	*in situ*
48	Anacardiaceae	*Schinus molle* L.	SRL-19, ERL-164	Pirul	4	Ornamental = 6	0		0.0098		0.0083		0		0		0		Sol	0.000026	AC	W	E, P, T, Ti	*ex situ*
49	Annonaceae	*Annona cherimola* Mill.	Photo record	Chirimoya	1		0		0		0		0.0088		0		0		Sol	0.000026	AC	D	P, Prp	*ex situ*
52	Apiaceae	*Ammi majus* L.	SRL-24, ERL-13, ERL-81, Erl-131, ERL-184	Encaje	2	Ornamental = 47, ceremonial = 20	0		0		0		0		0.0469		0		Sol	0.000205	EAAA	D	E, P, Prp, T, Ti	*ex situ*
53	Apiaceae	*Apium leptophyllum* (Pers.) F.Muell. ex Benth.	SRL-1525				0		0		0		0		0		0		Bal, BG, Sol	0	Ixc	W	T	*in situ*
54	Apiaceae	*Coriandrum sativum* L.	ERL-40, ERL-135, ERL-236	Cilandro	2	Edible = 100	0		0		0		0.0610		0		0		Sol	0.000205	EAAA	D	E, P, Prp, T, Ti	*ex situ*
55	Apiaceae	*Daucus carota* L.	Photo record	Zanahoria	1	Edible = 100	0		0		0		0.0075		0		0		Sol	0	EAAA	D	P, Prp	*ex situ*
56	Apiaceae	*Eryngium bonplandii* F.Delaroche	RLF-6, SRL-132, SRL-384, SRL-1247	Ojo de gallo	1		0		0		0	-0.9929	0		0		0		BEA, Paz	0.003360	Ixc	W	G	*in situ*
57	Apiaceae	*Eryngium comosum* F.Delaroche	RLF-127	espinuda	1		0		0		0	-1.0487	0		0		0		Me	0	Ixc	W	G	*in situ*
58	Apiaceae	*Eryngium pectinatum* C.Presl ex DC.	RLF-52, SRL-315		1		0		0		0	-1.0295	0		0		0		BEA, BEC	0.001155	Ixc	W	G	*in situ*
59	Apiaceae	*Foeniculum vulgare* Mill.	SRL-72, ERL-229	Hinojo	2		0		0		0.0119		0.0066		0		0		Sol	0.000051	EAAA	W	P, Prp	*ex situ*
60	Apiaceae	*Petroselinum crispum* (Mill.) Fuss	ERL-72	Perejil	2		0		0		0		0.0263		0		0		Sol	0.000128	EAAA	D	P, Prp	*ex situ*
61	Apiaceae		RLF-165		1		0		0		0		0		0		0		NE, TS	0	Nat-Uk	W	F, T, Ur	*ex situ*
75	Apocynaceae	*Asclepias curassavica* L.	ERL-242		1	Ornamental = 6	0		0	0.0738	0		0		0		0		Sol	0.000026	Ixc	W	P, Prp	*ex situ, in situ*
76	Apocynaceae	*Asclepias linaria* Cav.	RLF-35, SRL-131	Romero cimarrón	1		0		0	-2.1063	0		0		0		0		BEA	0	Ixc	W		
64	Apocynaceae	*Cascabela thevetia* (L.) Lippold	SRL-1336		1		0		0		0	-1.0487	0		0		0		CaCe	0	Ixc	W	G	*in situ*
78	Apocynaceae	*Funastrum elegans* (Decne.) Schltr.	SRL-443, SRL-1153, SRL-1544		1		0	-1.0765	0		0		0		0		0		BEA, Sol	0	Ixc	W	F	*in situ*
79	Apocynaceae	*Huernia macrocarpa* Schweinf. ex K.Schum.	Photo record	Órgano de Tehuacán	1	Ornamental = 6	0		0		0		0		0		0		Sol	0.000026	EAAA	W	P, Prp	*ex situ*
77	Apocynaceae	*Matelea purpusii* Woodson	SRL-1123	Tecacholo	2		0		0		0.0148	0.087	0.0022	-0.5798	0		0		BEA, Pal, Sol	0	Ixc	W	G, P, T	*in situ*
80	Apocynaceae	*Metastelma* sp.	RLF-321				0		0		0		0		0		0		Iz	0	Ixc	W		
62	Apocynaceae	*Nerium oleander* L.	ERL-103, ERL-123, SRL-178	Adelfa, laurel	2	Ornamental = 35, ceremonial = 14	0		0		0		0		0.004		0		Sol	0.000153	EAAA	D	P, Prp, Ti	*ex situ*
63	Apocynaceae	*Plumeria rubra* L.	Photo record	Cacalosuchil	2	Ornamental = 12	0		0.0156	1.0147	0		0		0.007	0.608	0		CaMy, Sol	0.000051	Ixc	W	G, P, Prp	*ex situ, in situ*
81	Apocynaceae		SRL-397	Tecacholo corriente			0		0		0		0		0		0		BEA	0	Ixc	W		
65	Araceae	*Zantedeschia aethiopica* (L.) Spreng.	SRL-220, ERL-203	Cartucho	2	Ornamental = 53, ceremonial = 17	0		0.0252		0		0		0.12		0		Pal, Sol	0.000230	EAAA	D	P, Prp, Ti	*ex situ*
66	Araliaceae	*Aralia humilis* Cav.	SRL-1482, SRL-1507	Mata gallina	3	Ornamental = 6	0		0	-0.3989	0		0		0		0		BEA, Sol	0.000026	Ixc	W	G, P, T	*in situ*
67	Araliaceae	*Schefflera* sp.	Photo record		1	Ornamental = 6	0		0		0		0		0		0		Sol	0.000026	EAAA	W	P, Ti	*ex situ*
68	Arecaceae	*Brahea dulcis* (Kunth) Mart.	RLF-155, RLF-191,SRL-462, SRL-463, SRL-1192, SRL-1193	Palma criolla	11	Ornamental = 35, ceremonial = 1, firewood = 100, 1ornamental = 95	0.0092	7.1968	0.0241	6.7574	0.0035	4.3551	0.0015	3.3156	0	4.1723	0.0086	3.952	BEA, BEC, BG, BN, Iz, Me, Pal, Palm, Sol, TS	0.105714	Ixc	W	E, F, G, P, T, Ti	*in situ*
69	Arecaceae	*Brahea dulcis x B. calcarea* Mart. x Liebm.	SRL-1229	Palma media sierra	6	1Ornamental = 95	0		0.0049	0.1754	0		0		0	-0.1118	0	-0.4762	BEA	0	Ixc	W	G, P	*in situ*
70	Arecaceae	*Brahea calcarea* Liebm.	SRL-219, SRL-461, SRL-1194	Palma blanca	4		0		0.0042	0.8205	0		0		0		0		BEA, Me, Sol	0	Ixc	W	G, P, Ti	*ex situ, in situ*
71	Arecaceae	*Phoenix canariensis* Chabaud	Photo record	Palma	2	Ornamental = 18, ceremonial = 1	0		0		0		0		0		0		Sol	0.000077	EAAA	W	P, Prp, T, Ti	*ex situ*
72	Arecaceae	*Washingtonia filifera* (Linden ex André) H.Wendl. ex de Bary	Photo record	Palma	1	Ornamental = 12	0		0		0		0		0		0		Sol	0.000051	AC	W	P, Prp, T, Ti	*ex situ*
73	Arecaceae		ERL-50	Palmera	1	Ornamental = 6	0		0		0		0		0		0		Sol	0.000026	EAAA	W	P, T, Ti	*ex situ*
74	Aristolochiaceae	*Aristolochia teretiflora* Pfeifer	SRL-1130	Orejita de ratón	2		0		0		0.0123	-0.364	0		0		0		Sol, TS	0	Ixc	W	G, T, Ur	*in situ*
6	Asparagaceae	*Agave americana* L.	Photo record	Maguey de pulque, Maguey de listón	4	Ornamental = 47, 18 = 30	0		0		0.0038		0		0		0		Sol, TS	0.000205	Mex	D	P, Prp, Ti	*ex situ*
9	Asparagaceae	*Agave applanata* Lem. ex Jacobi	Photo record	Maguey cenizo	1		0		0		0		0		0		0		Me, Pal, TS	0	Ixc	W	G, T	*in situ*
10	Asparagaceae	*Agave kerchovei* Lem.	Photo record	Maguey rabo de león	3	Edible = 20	0.0020	-0.2532	0		0		0.0148	-0.8621	0		0		Iz, Pal	0.001780	Ixc	W	F, G	*in situ*
11	Asparagaceae	*Agave potatorum* Zucc.	RLF-285, SRL-403, SRL-1209	Maguey papalomé	8	Fodder = 5, ornamental = 29, medicinal = 5, edible = 25, 18 = 20	0.0068	6.6941	0.046	5.3787	0.0388	5.4489	0.0717	3.9275	0		0		BEA, Iz, Me, Pal, Palm, SB, Sol, TS	0.020100	Ixc	W	E, F, G, P, Prp, T, Ti	*ex situ, in situ*
12	Asparagaceae	*Agave salmiana* Otto ex Salm-Dyck subsp. *tehuacanensis* (Karw. ex Salm-Dyck) García-Mend.	Photo record	Maguey cimarrón	10	Ornamental = 12	0.0022	6.3299	0.0098	3.672	0.0085	4.315	0	3.1267	0		0.0104	3.0362	BEA, BN, Pal, Palm, Sol, TS	0.009780	Ixc	W	F, G, P, Prp, T, Ti	*ex situ, in situ*
13	Asparagaceae	*Agave scaposa* Gentry	Photo record	Maguey potrero	3		0		0		0.0074	2.0018	0		0		0		BEM, Sol	0.000026	Ixc	W	G, P, Ti	*ex situ, in situ*
14	Asparagaceae	*Agave stricta* Salm-Dyck	SRL-1520		1		0		0	-0.0825	0		0		0		0		Me, Sol	0	Ixc	W	G, P, Ti	*ex situ, in situ*
15	Asparagaceae	*Agave titanota* Gentry	SRL-404	Maguey tieso	2		0	-0.6097	0		0		0	-1.1696	0		0		Iz	0	Ixc	W	F, G	*in situ*
16	Asparagaceae	*Agave triangularis* Jacobi	SRL-437	Maguey rabo de león, maguey tieso	3		0	-0.2987	0		0		0	-1.0057	0		0		Iz	0	Ixc	W	F, G	*in situ*
17	Asparagaceae	*Agave tequilana* F.A.C.Weber	Photo record	Agave azul	1	Ornamental = 6	0		0		0		0		0		0		Pal, Sol	0.000026	Mex	D	P, Prp, Ti	*ex situ*
8	Asparagaceae	*Agave vivipara* L.	SRL-235, SRL-1353, SRL-1389	Maguey espadín	5	Ornamental = 6	0		0.0147	1.6977	0.0021	2.4585	0		0		0		CaCe, Iz, Pal, SB, Sol, Ts	0.002851	Ixc	D, W	G, P, Prp	*ex situ, in situ*
553	Asparagaceae	*Beaucarnea stricta* Lem.	RLF-149	Sotol	2	Ceremonial = 1	0		0		0		0		0	-0.75	0		Iz	0.012638	Ixc	W	G, P	*in situ*
554	Asparagaceae	*Dasylirion serratifolium* (Karw. ex Schult. & Schult.f.) Zucc.	RLF-156, SRL-420, SRL-1473, SRL-1521	Cucharilla, manita	5	Edible = 95, ceremonial = 5	0.0019	0.3359	0		0		0.1098	0.1909	0	-0.6392	0		BG, Me	0.000547	Ixc	W	F, G	*in situ*
50	Asparagaceae	*Echeandia paniculata* Rose	SRL-442, SRL-1114	Cebolla de cacalote	3		0		0		0	-0.6167	0	-1.0101	0		0		BEA, Iz, Me	0.003272	Ixc	W	G	*in situ*
51	Asparagaceae	*Echeandia* sp.	SRL-319	Pasto	1		0	-1.0765	0		0		0		0		0		BEA, BEC	0	Ixc	W	F	*in situ*
25	Asparagaceae	*Milla biflora* Cav.	SRL-1537	Huele de noche			0		0		0		0		0		0		Me, Palm, TS	0	Ixc	W	T	*in situ*
555	Asparagaceae	*Nolina longifolia* (Karw. ex Schult. & Schult.f.) Hemsl.	SRL-228	Sotol	3		0		0		0		0		0	-1.1913	0		BEA, Me	0	Ixc	W	G	*in situ*
19	Asparagaceae	*Yucca periculosa* Baker	SRL-1505	Tohuizote	4		0		0		0		0		0		0		AA	0	Ixc	W	G	*in situ*
18	Asparagaceae	*Yucca gigantea* Lem.	SRL-1532	Huizote, pita, tehuizote	2	Ornamental = 12	0		0		0		0.0066		0		0		Sol	0.000051	Mex	W	P, Prp	*ex situ*
215	Balsaminaceae	*Impatiens walleriana* Hook.f.	Photo record	Belén	1	Ornamental = 12	0		0		0		0		0		0		Sol	0.000051	EAAA	W	P, Prp	*ex situ*
216	Basellaceae	*Anredera cordifolia* (Ten.) Steenis	ERL-119		1		0		0		0		0		0		0		Sol	0	EAAA	W	P, Prp	*ex situ*
217	Berberidaceae	*Berberis pallida* Hartw. ex Benth.	SRL-216, SRL-217, SRL-401, SRL-1235, SRL-1399, SRL-1449	Palo tostado	2	Firewood = 100	0	-0.5351	0		0		0		0		0.0086	-1.2037	BEA, Iz, Me, Palm	0.002781	Ixc	W	F, G	*in situ*
218	Berberidaceae	*Berberis* sp.	SRL-1428				0		0		0		0		0		0		BEM	0	Ixc	W		
219	Bignoniaceae	*Jacaranda mimosifolia* D.Don	ERL-226	Jacaranda	3	Ornamental = 12	0		0.0074		0		0		0		0		Sol	0.000051	AC	D	P, Ti	*ex situ*
220	Bignoniaceae	*Podranea ricasoliana* (Tanfani) Sprague	ERL-252	Flor tronador	2	Ornamental = 6	0		0		0		0		0		0		Sol	0.000026	EAAA	W	P, Ti	*ex situ*
221	Bignoniaceae	*Tecoma stans* (L.) Juss. ex Kunth	RLF-13, RLF-56, RLF-249, SRL-438, SRL-465, SRL-1307	Tronadora	2		0	-0.3922	0		0.0013	-0.6459	0		0		0		BEA, BN, Iz, Me	0.008107	Ixc	W	F, G	*in situ*
222	Boraginaceae	*Antiphytum caespitosum* I.M.Johnst.	RLF-125, SRL-99, SRL-1400, SRL-1466	Semonilla	1	Medicinal = 10	0		0		0.0317	0.3143	0		0		0		BN, Me, Palm	0.007127	Ixc	R-W, W	G	*in situ*
223	Boraginaceae	*Borago officinalis* L.	SRL-52	Gordolobo	1		0		0		0.0046		0		0		0		Sol	0	EAAA	W	P, Prp	*ex situ*
224	Boraginaceae	*Cordia curassavica* (Jacq.) Roem. & Schult.	SRL-1392		1		0	-1.0765	0		0		0		0		0		CaCe	0	Ixc	W	F	*in situ*
401	Boraginaceae	*Nama dichotoma* (Ruiz & Pav.) Choisy	SRL-98, SRL-1182		1		0	-1.0765	0		0		0		0		0		BN, Palm	0	Ixc	W	F	*in situ*
402	Boraginaceae	*Nama* sp.	SRL-166				0		0		0		0		0		0		Pal	0	Ixc	W	T	*in situ*
403	Boraginaceae	*Wigandia urens* (Ruiz & Pav.) Kunth	SRL-1352	Chichicasle de tierra caliente			0		0		0		0		0		0		CaCe	0	Ixc	W		
225	Brassicaceae	*Brassica oleracea* L.	Photo record	Brócoli, Col	1		0		0		0		0.0075		0		0		Sol	0.000026	EAAA	D	P, Prp	*ex situ*
226	Brassicaceae	*Brassica rapa* L.	SRL-1536	Mostaza	2		0.0065		0		0		0.0038		0		0		Bal, Sol, TS	0.002183	Nat-EAAA	R-W	G, T, Ur	*ex situ*
229	Brassicaceae	*Capsella bursa-pastoris* (L.) Medik.	SRl-182, SRL-1324	Lentejilla	1		0		0		0		0		0		0		Bal, Sol	0	Nat-EAAA	R-W	G, T, Ur	*ex situ*
230	Brassicaceae	*Descurainia virletii* (E.Fourn.) O.E.Schulz	SRL-35	Mostaza	2		0	-0.6097	0		0		0	-1.1696	0		0		Bal, Sol	0	Ixc	R-W	F, G	*in situ*
227	Brassicaceae	*Eruca vesicaria* (L.) Cav.	RLF-309, SRL-39, SRL-1131	Jaramón	2	Fodder = 40	0.0323		0		0		0		0		0		Bal, Sol, TS	0.000026	Nat-EAAA	R-W	F, G, T, Ur	*ex situ*
231	Brassicaceae	*Lepidium virginicum* L.	ERL-109, RLF-70, RLF-103, RLF-179, SRL-1320	Lentejilla	3	Ornamental = 35	0	0.6404	0	0.4307	0.0097	0.2534	0		0		0		Bal, BEA, Sol	0.000153	Ixc	R-W	F, G, P, T	*in situ*
232	Brassicaceae	*Matthiola incana* (L.) R.Br.	ERL-20, ERL-170	Alelía	2	Ornamental = 18, ceremonial = 10	0		0.0042		0		0		0.0261		0		Sol	0.000077	EAAA	D	P, Prp	*ex situ*
234	Brassicaceae	*Nasturtium officinale* R.Br.	SRL-199	Berro	2	Edible = 15	0		0		0		0.0132		0		0		VR	0	Nat-EAAA	R-W	G	*ex situ*
233	Brassicaceae	*Raphanus sativus* L.	SRL-44, ERL-136, ERL-162, ERL-179	Rábano	2		0		0		0		0.0445		0		0		Sol	0.000153	EAAA	D	P, Prp	*ex situ*
235	Brassicaceae		SRL-1319				0		0		0		0		0		0		Bal	0	Nat-Uk	R-W	T	*ex situ*
236	Bromeliaceae	*Ananas comosus* (L.) Merr.	Photo record	Piña	2	Edible = 10	0		0		0.0014		0		0		0		Sol	0.000026	AC	D	P, Prp	*ex situ*
237	Bromeliaceae	*Catopsis compacta* Mez	RLF-335, SRL1253	Soluche de jarrita	5	Ornamental = 6, ceremonial = 22	0	3.5020	0	1.1246	0	2.2591	0		0	1.1707	0		BEA, Iz, Sol	0.000026	Ixc	W	G, P, Ti	*ex situ, in situ*
238	Bromeliaceae	*Hechtia oaxacana* Burt-Utley, Utley & García-Mend.	SRL-405, SRL-1524	Lechugilla	1	Fodder = 10	0.0384	-0.866	0		0		0		0		0		BG, Iz, Me	0.002571	Ixc	W	F, G	*in situ*
239	Bromeliaceae	*Hechtia* sp.	SRL-1393	Lechugilla de terreno caliente			0		0		0		0		0		0		CaCe	0	Ixc	W		
240	Bromeliaceae	*Tillandsia acyrostachys* E.Morren ex Baker	SRL-1492		2	Ceremonial = 2	0		0.0221	-0.4059	0		0		0	-0.9895	0		Me	0	Ixc	W	Ti	*in situ*
241	Bromeliaceae	*Tillandsia bourgaei* Baker	SRL-1197	Soluche blanco	3		0	-0.3766	0.0221	-0.5262	0		0	-1.0578	0		0		BEA	0	Ixc	W	G	*in situ*
242	Bromeliaceae	*Tillandsia grandis* Schltdl.	SRL-1472	Jarrilla	3	Ornamental = 6, ceremonial = 5	0.0290	2.8484	0.0027	0.6724	0		0		0.0093	0.7779	0		CaCe, Me, Sol	0.000026	Ixc	W	G, P, Ti	*ex situ, in situ*
243	Bromeliaceae	*Tillandsia gymnobotrya* Baker	SRL-1201, SRL-1435	Soluche blanco, soluche de flor colorada	5	Fodder = 30, ceremonial = 2	0.0827	0.2377	0.0221	-0.0008	0	-0.2934	0.0044	-0.6966	0	-0.7305	0		BEM	0	Ixc	W	G	*in situ*
244	Bromeliaceae	*Tillandsia juncea* (Ruiz & Pav.) Poir.	RLF-81, SRL-1246, SRL-1254	Soluche	3	Ceremonial = 2	0	-0.3767	0.0221	-0.5262	0		0		0	-1.1767	0		BEA, Sol		Ixc	W	G	*in situ*
245	Bromeliaceae	*Tillandsia macdougallii* L.B.Sm.	RLF-84, SRL-224, SRL-1242, SRL-1250	Soluche	3	Ornamental = 6	0	2.8801	0.0221	1.203	0		0	1.116	0		0		BEA, Pal, Sol, VR	0.000026	Ixc	W	G, P, Ti	*ex situ, in situ*
246	Bromeliaceae	*Tillandsia recurvata* (L.) L.	SRL-211	Soluchito	3	Ornamental = 6	0.0081	-0.0731	0.0221	-0.1783	0	-0.4357	0		0		0		Palm, Sol	0.000026	Ixc	R-W	G, T	*in situ*
247	Bromeliaceae	*Tillandsia usneoides* (L.) L.	SRL-138, SRL-1245	Apasle	5	Ornamental = 29, ceremonial = 2	0.0144	4.4242	0.009	2.5721	0		0		0	1.7881	0		BEA, BEM, Pal, Sol	0.000128	Ixc	W	G, P, Prp, Ti	*ex situ, in situ*
248	Bromeliaceae	*Tillandsia* sp.	SRL-1244	Soluche	2	Ornamental = 6	0	2.5691	0.0221	0.9401	0		0		0		0		BEA, Sol	0.000026	Ixc	W	G, P, Ti	*ex situ, in situ*
249	Bromeliaceae	*Tillandsia* sp.	SRL-1252	Soluche cimarrón, soluche ixtludo	3	Ceremonial = 2	0	2.879	0.0221	1.0465	0		0		0	0.7241	0		BEA, Pal	0	Ixc	W	G, P, Ti	*ex situ, in situ*
250	Bromeliaceae	*Tillandsia* sp.	SRL-1243	Soluche	4	Ceremonial = 2	0	-0.0656	0.0221	-0.2635	0		0	-0.894	0	-0.9536	0		BEA	0	Ixc	W	G	*in situ*
251	Buddlejaceae	*Buddleja parviflora* Kunth	ERL-197, SRL-371, SRL-1207, SRL-1522	Lengua de vaca, tepozán	3	Firewood = 100	0		0		0.0025	-0.1805	0		0		0.0071	-0.6566	BEA, BG, Palm, Sol	0.001533	Ixc	W	G, T	*in situ*
252	Buddlejaceae	*Buddleja* sp.	RLF-83, SRL-30		1		0	-1.0519	0		0		0		0		0		BEA, BN, Sol	0.000917	Ixc	W	F	*in situ*
253	Buddlejaceae	*Buddleja* sp.	SRL-118				0		0		0		0		0		0		BEA	0	Ixc	W		
254	Buddlejaceae	*Buddleja* sp.	RLF-218, RLF-284	Tepozán			0		0		0		0		0		0		Iz	0	Ixc	W		
255	Burseraceae	*Bursera biflora* (Rose) Standl.	RJS-11, RLF-122, SRL-1219	Copal colorado, copal amarillo, copal criollo	7	Ceremonial = 99	0		0	1.805	0	3.1524	0		0.0278	2.8995	0.0036	1.9672	Iz, Me, SB	0	Ixc	W	G, P, Prp, Ti	*ex situ, in situ*
256	Burseraceae	*Bursera fagaroides* (Kunth) Engl.	SRL-349	Copalillo	3		0.0046	-0.2997	0		0		0		0	-1.1371	0.0036	-1.4632	Me	0.000149	Ixc	W	F, G	*in situ*
257	Burseraceae	*Bursera galeottiana* Engl.	RLF-323	Cuajilote			0		0		0		0		0		0		Iz	0	Ixc	W		
258	Burseraceae	*Bursera morelensis* Ramírez	SRL-1345				0		0		0		0		0		0		CaCe	0	Ixc	W		
259	Burseraceae	*Bursera pontiveteris* Rzed., Calderón & Medina	SRL-1271	Copalillo blanco	2		0		0		0		0		0	-1.0042	0.0036	-1.2693	Me	0	Ixc	W	G, P	*in situ*
260	Burseraceae	*Bursera schlechtendalii* Engl.	SRL-1367	Aceitillo	2		0.0027	-0.7685	0		0	-0.9186	0		0		0		CaCe	0	Ixc	W	F	*in situ*
261	Burseraceae	*Bursera submoniliformis* Engl.	SRL-1341, SRL-1346	Copalillo blanco			0		0		0		0		0		0		CaCe	0	Ixc	W		
262	Cactaceae	*Acanthocereus subinermis* Britton & Rose	Photo record	Nopalito de cruz	1		0		0		0		0		0		0		Sol	0.000026	Mex	W	P, Prp	*ex situ*
263	Cactaceae	*Cephalocereus columna-trajani* (Karw. ex Pfeiff.) K.Schum.	Photo record	Cardón pachón, soldadillo	1		0		0	-0.1783	0		0		0		0		CaCe, Sol	0	Ixc	W	P, Ti	*ex situ, in situ*
264	Cactaceae	*Coryphantha retusa* (Pfeiff) Britton & Rose	Photo record	Bizniaga	1	Ornamental = 12	0		0.0074	0.3458	0		0		0		0		Me, Palm, Sol	0.000433	Ixc	W	P, Ti	*ex situ, in situ*
265	Cactaceae	*Escontria chiotilla* (A.A.Weber ex K.Schum.) Rose	Photo record	Jiotilla	1		0		0		0		0.0018		0		0		TS	0	TCV	D	Prp	*ex situ*
266	Cactaceae	*Ferocactus macrodiscus* (Mart.) Britton & Rose	SRL-402	Bizniaga	3	Ornamental = 6	0.0161	2.7969	0.0074	0.7647	0		0.0033	1.0957	0		0		Paz, Sol	0.000484	Ixc	W	F, P, Ti	*ex situ, in situ*
267	Cactaceae	*Ferocactus recurvus* (Mill.) Borg	SRL-1419	Bizniaga grande	3	Ornamental = 12	0.0161	3.2785	0.0074	1.2215	0		0.0033	1.4159	0		0		Paz, Sol, TS	0.001008	Ixc	W	F, P, T, Ti	*ex situ, in situ*
268	Cactaceae	*Hylocereus undatus* (Haw.) Britton & Rose	Photo record	Pitahaya	2	Ornamental = 35	0		0		0		0.0016		0		0		Sol	0.000153	Mex	D	P, Prp	*ex situ*
270	Cactaceae	*Mammillaria carnea* Zucc. ex Pfeiff.	SRL-387	Biznaga			0		0		0		0		0		0		CaCe	0	Ixc	W		
271	Cactaceae	*Mammillaria haageana* Pfeiff.	SRL-387, SRL-1480	Bizniaga chiquita	2	Ornamental = 18, ceremonial = 2	0		0.0074	0.8648	0		0		0	0.4819	0		BEA, Iz, Me, Palm, Sol	0.004228	Ixc	W	P, Ti	*ex situ, in situ*
272	Cactaceae	*Mammillaria sphacelata* Mart.	Photo record	Biznaga	2	Ceremonial = 2	0		0.0074	0.6323	0		0		0	0.719	0		BEA, BN, Me, Pal, Sol, TS	0.005848	Ixc	R-W, W	P, T, Ti	*ex situ, in situ*
273	Cactaceae	*Mammillaria* sp.	Photo record	Biznaga	1		0		0.0074	-1.9051	0		0		0		0		CaMy	0	Ixc	W		
274	Cactaceae	*Mammillaria* sp.	Photo record	Bizniaga	1	Ornamental = 6	0		0.0074	0.1793	0		0		0		0		NE, Sol	0.000026	Ixc	W	P, Ti	*ex situ, in situ*
269	Cactaceae	*Marginatocereus marginatus* (DC.) Backeb.	SRL-237	Órgano	5	Ornamental = 59, ceremonial = 40	0		0		0		0		0		0		AA, Sol	0.000256	TCV	W	P, Prp, T, Ti	*ex situ*
275	Cactaceae	*Opuntia depressa* Rose	SRL-238	Nopal de coyote	3		0.007	0.0052	0		0.0139	-0.1499	0		0		0		BEA,TS	0	Ixc	W	F, G, T	*in situ*
276	Cactaceae	*Opuntia ficus-indica* (L.) Mill.	Photo record	Nopal de castilla, nopal pelón	2	Edible = 100	0		0		0		0.0536		0		0		Sol	0.000281	Mex	D	P, Prp	*ex situ*
277	Cactaceae	*Opuntia huajuapensis* Bravo	SRL-239	Nopal	3		0.0072	1.1617	0		0		0		0		0		BEA, BEC, BN, Iz, Me, Palm, Paz, TS	0.014065	Ixc	R-W, W	F, G, T, Ti	*in situ*
278	Cactaceae	*Opuntia lasiacantha* Pfeiff.	SRL-477	Nopal pachón	2	Edible = 100	0		0		0		0.0433	2.0372	0		0		Sol, TS	0.000179	Ixc	R-W, W	P, Prp, T, Ti	*ex situ, in situ*
279	Cactaceae	*Opuntia* sp.	SRL-236	Nopal amarillo	3		0.0072		0		0		0		0		0		Sol	0	TCV	W	F, P, Prp, T, Ti	*ex situ*
280	Cactaceae	*Opuntia* sp.	Photo record	Nopal de coyote, nopal tuna roja	3		0.0072	4.1969	0		0		0	2.0015	0		0		Palm, Sol	0.000026	Ixc	W	F, Prp, T, Ti	*ex situ, in situ*
281	Cactaceae	*Opuntia* sp.	Photo record	Nopal de sacristán	2		0.0072	1.0967	0		0		0		0		0		Palm, TS	0	Ixc	W	F, P, T, Ti	*in situ*
282	Cactaceae	*Pachycereus weberi* (J.M. Coult.) Backeb.	Photo record	Cardón verde			0		0		0		0		0		0		SB	0	Ixc	W		
283	Cactaceae	*Pseudomitrocereus fulviceps* (F.A.C.Weber ex K.Schum.) Bravo & Buxb.	SRL-1451, SRL-1501	Cardón			0		0		0		0		0		0		CaMy	0	Ixc	W		
284	Cactaceae		SRL-1452				0		0		0		0		0		0		CaMy	0	Ixc	W		
285	Calochortaceae	*Calochortus barbatus* (Kunth) J.H.Painter	SRL-1204				0		0		0		0		0		0		BEA	0	Ixc	W		
286	Campanulaceae	*Diastatea micrantha* (Kunth) McVaugh	SRL-156, SRL-157		2		0	-0.7655	0	-1.4404	0		0		0		0		BEA, Me, Pal	0	Ixc	W	F	*in situ*
763	Cannabaceae	*Celtis caudata* Planch.	ERL-79, ERL-155, ERL-194, ERL-222, SRL-1475	Malintze, moralillo	5		0.0161	1.0117	0		0		0	-0.2066	0		0		Me, Sol	0.000179	Ixc	W	P, T	*in situ*
287	Cannaceae	*Canna indica* L.	SRL-57, ERL-43, ERL-217	Platanillo	2	Ornamental = 35, ceremonial = 10	0		0		0		0		0		0		Sol	0.000153	TCV	W	P, Prp	*ex situ*
288	Capparaceae	*Capparis pringlei* Briq.	SRL-1354				0		0		0		0		0		0		CaCe	0	Ixc	W		
213	Caprifoliaceae	*Scabiosa atropurpurea* L.	ERL-23, ERL-239	Barín	2	Ornamental = 6	0		0		0		0		0		0		Sol	0.000026	EAAA	W	P, Prp	*ex situ*
767	Caprifoliaceae	*Valeriana* sp.	RLF-28, RLF-199, SRL-1300		1		0	-1.0758	0		0		0		0		0		VR	0.000026	Ixc	W	F	*in situ*
289	Caricaceae	*Carica papaya* L.	Photo record	Papaya	1	Edible = 5	0		0		0		0.0053		0		0		Sol	0.000051	Mex	D	P, Prp	*ex situ*
290	Caryophyllaceae	*Dianthus caryophyllus* L.	Photo record	Clavel	2		0		0.0118		0		0		0.0147		0		Sol	0	EAAA	D	P, Prp	*ex situ*
291	Casuarinaceae	*Casuarina equisetifolia* L.	SRL-41	Ocote corriente, pino	4	Ornamental = 35	0		0.0588		0.0015		0		0		0.0095		Sol	0.000153	EAAA	W	P, Ti	*ex situ*
292	Celastraceae	*Acanthothamnus aphyllus* (Schltdl.) Standl.	SRL-1504				0		0		0		0		0		0		Me	0	Ixc	W		
293	Celastraceae	*Cassine xylocarpa* Vent.	SRL-1334				0		0		0		0		0		0		CaCe	0	Ixc	W		
294	Chenopodiaceae	*Chenopodium berlandieri* Moq.	SRL-1139	Quelite de manteca, flor de huizontle	2	Edible = 15	0.0046	-0.3023	0		0		0.0222	-0.788	0		0		Sol	0.000026	Ixc	R-W	F, G, T	*in situ*
295	Chenopodiaceae	*Chenopodium murale* L.	RLF-184, SRL-194, SRL-1121, SRL-1140, SRL-1321	Quelite de guajolote	3	Fodder = 10, medicinal = 40	0.0054		0		0		0.0081		0		0		Bal, Sol	0.000128	Nat-EAAA	R-W	F, G, T, Ur	*ex situ*
296	Chenopodiaceae	*Dysphania ambrosioides* (L.) Mosyakin & Clemants	ERL-32, ERL-33, ERL-168, RLF-89, SRL-1136	Epazote	4	Edible = 100	0		0		0.0123	1.3678	0.0237	0.7706	0		0		Bal, Sol	0.000179	Ixc	R-W	E, P, Prp, T	*in situ*
297	Chenopodiaceae	*Spinacia oleracea* L.	Photo record	Espinaca	1		0		0		0		0.0053		0		0		Sol	0	EAAA	D	P, Prp	*ex situ*
298	Cistaceae	*Helianthemum* sp.	RLF-17				0		0		0		0		0		0		BEA	0	Ixc	W		
299	Commelinaceae	*Commelina erecta* L.	RLF-19, RLF-73, SRL-159		2		0	-0.6240	0	-1.3004	0		0		0		0		BEA, Pal	0.005276	Ixc	R-W, W	F	*in situ*
300	Commelinaceae	*Commelina* sp.	SRL-48		1		0		0		0		0		0		0		Sol	0	EAAA	W	P, Prp	*ex situ*
301	Commelinaceae	*Gibasis consobrina* D.R.Hunt	RLF-190, SRL-430	Milpa, lengua de cucho			0		0		0		0		0		0		BG, Iz	0	Ixc	W		
302	Commelinaceae	*Gibasis geniculata* (Jacq.) Rohweder	ERL-44		1	Ornamental = 6	0		0		0		0		0		0		Sol	0.000026	TCV	W	P, Prp	*ex situ*
303	Commelinaceae	*Tradescantia crassifolia* Cav.	SRL-149		1		0		0		0	-1.0487	0		0		0		Me	0	Ixc	W	G	*in situ*
304	Commelinaceae	*Tripogandra purpurascens* (S.Schauer) Handlos	RLF-15		1		0	-0.8960	0		0		0		0		0		BEA, Me	0.000920	Ixc	W	F, G	*in situ*
84	Compositae	*Acourtia scapiformis* (Bacig.) B.L.Turner	SRL-163		1		0		0	-2.1063	0		0		0		0		BEA	0	Ixc	W		
85	Compositae	*Acourtia* sp.	SRL-215,SRL-1468	Oreja de conejo			0		0		0		0		0		0		Me	0.004331	Ixc	W		
86	Compositae	*Adenophyllum glandulosum* (Cav.) Strother	SRL-1264		1		0	-1.0766	0		0		0		0		0		Me	0	Ixc	W	F	*in situ*
87	Compositae	*Ageratina espinosarum* (A.Gray) R.M.King & H.Rob.	RLF-36, SRL-114, SRL-291, SRL-325, SRL-363, SRL-1279		2		0	0.0391	0		0	-0.347	0		0		0		BEA, BEC, BG, BN, Iz, Me, Pal, Palm, Sol, TS	0.009661	Ixc	R-W, W	F, G, T, Ur	*in situ*
88	Compositae	*Ageratina mairetiana* (DC.) R.M.King & H.Rob.	SRL-186, SRL-390	Hierba de ángel	3	Ornamental = 6, medicinal = 15	0	3.3978	0	0.9653	0.15	5.7983	0		0		0		BEA, Pal, Sol	0.004801	Ixc	R-W, W	F, P, T, Ti	*ex situ, in situ*
89	Compositae	*Ageratina tomentella* (Schrad.) R.M.King & H.Rob.	RLF-217, SRL-119, SRL-289, SRL-335, SRL-391, SRL-1191, SRL-1398, SRL-1406		1		0	-0.7561	0		0		0		0		0		BEA, BG, BN, Iz, Me, Palm	0.011943	Ixc	R-W, W	F	*in situ*
90	Compositae	*Ageratina* sp.	RLF-116, SRL-74	Hierba de ángel	3		0	-0.2987	0	-1.0819	0	-0.6123	0		0		0		Me, Sol	0	Ixc	W	F, G	*in situ*
91	Compositae	*Ageratina* sp.	RLF-4, SRL-153, SRL-287	Niebla	2		0	-0.6843	0	-1.36	0		0		0		0		BEA, BN, Me	0.003029	Ixc	W	F	*in situ*
92	Compositae	*Ageratina* sp.	SRL-208	Oreganillo	1		0		0		0	-0.8137	0		0		0		Pal, Sol	0	Ixc	W	G, T	*in situ*
93	Compositae	*Ageratum tehuacanum* R.M.King & H.Rob.	RLF-26, SRL-113	Cara blanca	1		0	-1.0765	0		0		0		0		0		BEA, BN	0	Ixc	W	F	*in situ*
94	Compositae	*Ambrosia psilostachya* DC.	RLF-9		1	Medicinal = 5	0		0		0	-0.5778	0		0		0		BEA, BN, Me, Paz	0.006536	Ixc	R-W, W	G	*in situ*
97	Compositae	*Archibaccharis serratifolia* (Kunth) S. F. Blake	RLF-257, SRL-267, SRL-292, SRL-1241		1		0	-0.9975	0		0		0		0		0		BEA, BEC, BN, Iz	0.002943	Ixc	R-W, W	F	*in situ*
95	Compositae	*Artemisia ludoviciana* Nutt.	Photo record	Ajenjo, estafiate, hierba maistra	1	Medicinal = 10	0		0		0.0591		0		0		0		Sol	0.000026	TCV	W	P, Prp	*ex situ*
214	Compositae	*Baccharis conferta* Kunth	Photo record		1		0		0		0		0		0		0		Sol, TS	0.008509	Ixc	W	T	*in situ*
98	Compositae	*Baccharis salicina* Torr. & A.Gray	SRL-1151	Chamizo	1		0		0		0	-1.0487	0		0		0		BEA	0	Ixc	R-W, W	G	*in situ*
99	Compositae	*Barkleyanthus salicifolius* (Kunth) H.Rob. & Brettell	SRL-190, SRL-1531, ERL-27, ERL-83, ERL-190, ERL-218	Somiate	6	Ornamental = 65, firewood = 100	0.0323	0.9183	0	1.6175	0.0291	0.6711	0		0		0.0082	-0.2179	BG, Pal, Palm, Sol	0.000281	Ixc	R-W, W	F, G, T	*in situ*
100	Compositae	*Bidens bigelovii* A.Gray	RLF-140, RLF-196	Cahual cimarrón	1		0	-1.0765	0		0		0		0		0		VR	0	Ixc	W	F	*in situ*
101	Compositae	*Bidens pilosa* L.	SRL-4, SRL-1285	Oaxaqueña	2	Fodder = 40	0	-0.0737	0		0	-0.485	0		0		0		BG, Pal, Sol, TS	0.001353	Ixc	R-W, W	F, G, T, Ur	*in situ*
102	Compositae	*Bidens* sp.	RLF-221, SRL-316, SRL-395, SRL-1288		1		0	-0.6451	0		0		0		0		0		BEA, BEC, BG, Iz, Pal	0.016081	Ixc	W	F	*in situ*
103	Compositae	*Brickellia veronicifolia* (Kunth) A.Gray	RLF-11, RLF-203, RLF-206, SRL-293, SRL-361, SRL-1276, ERL-101	Oreganillo, orejita de ratón	3		0	0.4263	0		0	-0.1215	0		0		0		BEA, BN, Iz, Me, Pal, Palm, Sol, TS	0.015409	Ixc	R-W, W	F, G, T	*in situ*
104	Compositae	*Brickellia* sp.	SRL-1418		1		0	-1.0765	0		0		0		0		0		Paz	0	Ixc	R-W, W	F	*in situ*
105	Compositae	*Calendula officinalis* L.	SRL-49, ERL-22, ERL-24	Mercader amarillo	3	Ornamental = 18	0		0		0		0		0		0		Sol	0.000077	EAAA	D	E, P, Prp, T, Ti	*ex situ*
106	Compositae	*Carminatia alvarezii* Rzed. & Calderón	RLF-186, SRL-127, SRL-1308	Oaxaqueña	1		0	-1.0765	0		0		0		0		0		BEA, Iz, Me	0	Ixc	W	F	*in situ*
107	Compositae	*Chrysactinia mexicana* A.Gray	RLF-154, SRL-1163	Hierba de San Nicolás	1		0		0		0.0167	-0.3058	0		0		0		Palm	0	Ixc	W	G, P	*in situ*
109	Compositae	*Chrysanthemum morifolium* Ramat.	ERL-143, ERL-163, ERL-230	Cresentena, nora, teresita	2	Ornamental = 53, ceremonial = 29	0		0		0		0		0.1021		0		Sol	0.000230	EAAA	D	E, P, Prp, Ti	*ex situ*
110	Compositae	*Cirsium mexicanum* DC.	SRL-435	Lechuga cimarrón	2		0	-0.6097	0		0		0	-1.1696	0		0		BG, Pal	0	Ixc	W	F, G	*in situ*
111	Compositae	*Cirsium* sp.	SRL-400, SRL-1427	Espino del diablo, chicalote de monte	1		0	-1.0765	0		0		0		0		0		BEA, BEM	0	Ixc	W	F	*in situ*
112	Compositae	*Coreopsis* sp.	SRL-314		1		0	0.0527	0		0		0		0		0		BEA, BEC, BN, Me, Palm, Paz	0.042091	Ixc	R-W, W	F	*in situ*
113	Compositae	*Cosmos bipinnatus* Cav.	ERL-4, ERL-166, ERL-167, SRL-45, SRL-47	Jazmín	2	Ornamental = 24, ceremonial = 8	0		0		0		0		0		0		Sol	0.000102	Nat-Mex	W	E, P, Prp, T	*ex situ*
114	Compositae	*Dahlia apiculata* (Sherff) P.D.Sorensen	RLF-259, SRL-91, SRL-1199, ERL-133, ERL-148	Dalia corriente, ticurrichi	2	Ornamental = 12	0		0	1.0674	0		0		0	1.1027	0		BEA, BEM, Pal, Sol	0.000051	Ixc	W	G, P, Prp, Ti	*ex situ, in situ*
115	Compositae	*Dahlia coccinea* Cav.	RLF-96, RLF-260, SRL-423, SRL-1160, SRL-1186	Dalia	2		0		0	0.7547	0		0		0	1.1017	0		BEA, BEM, BG, Me, Pal, Sol	0	Ixc	W	G, P, Prp, Ti	*ex situ, in situ*
116	Compositae	*Dahlia* sp.	ERL-132, ERL-251, ERL-253, ERL-452	Dalia	2	Ceremonial = 8	0		0.015		0		0		0.0093		0		Sol	0	TCV	D	P, Prp	*ex situ*
117	Compositae	*Desmanthodium* sp.	SRL-270				0		0		0		0		0		0		BEA	0.006577	Ixc	W		
118	Compositae	*Dyssodia papposa* (Vent.) Hitchc.	RLF-240, SRL-5, SRL-410, SRL-1290	Cempasuchito	1		0	-1.0765	0		0		0		0		0		Iz, Pal, Sol	0	Ixc	R-W	F	*in situ*
119	Compositae	*Dyssodia* sp.	RLF-24, SRL-121, SRL-379		1		0		0	-2.1063	0		0		0		0		BEA	0	Ixc	W		
120	Compositae	*Erigeron karvinskianus* DC.	RLF-270		1		0	-1.0765	0		0		0		0		0		BEA	0	Ixc	W	F	*in situ*
121	Compositae	*Erigeron* sp.	SRL-409				0		0		0		0		0		0		Bal	0	Ixc	W	T	*in situ*
122	Compositae	*Flaveria trinervia* (Spreng.) C.Mohr	SRL-16	Romero cimarrón			0		0		0		0		0		0		Bal, Sol	0	Ixc	R-W	T	*in situ*
123	Compositae	*Galinsoga parviflora* Cav.	RLF-269, SRL-1176		1		0	-1.0765	0		0		0		0		0		BEA, Palm	0	Ixc	W	F	*in situ*
108	Compositae	*Glebionis coronaria* (L.) Cass. ex Spach	SRL-10, ERL-16, ERL-17, ERL-34, ERL-35, ERL-255, ERL-256	Linda	2	Ornamental = 29, ceremonial = 29	0		0.0131		0		0		0.0023		0		Sol	0.000128	EAAA	D	E, P, Prp, T	*ex situ*
124	Compositae	*Gnaphalium* sp.	RLF-188		1		0		0	-2.1063	0		0		0		0		Iz	0	Ixc	R-W, W		
125	Compositae	*Gnaphalium* sp.	SRL-297		1	Medicinal = 5	0		0		0	-0.6864	0		0		0		Paz	0	Ixc	R-W, W	G	*in situ*
126	Compositae	*Gochnatia hypoleuca* (DC.) A.Gray	SRL-1464				0		0		0		0		0		0		Me	0	Ixc	W		
127	Compositae	*Grindelia inuloides* Willd.	RLF-14, SRL-66, SRL-107, SRL-278, SRL-295, SRL-365, SRL-1547	Árnica	1	Medicinal = 25	0		0		0.0938	5.0025	0		0		0		BEA, BN, Pal, Palm, Paz, Sol	0.002068	Ixc	W	G, P, Prp	*ex situ, in situ*
128	Compositae	*Gymnosperma glutinosum* (Spreng.) Less.	RLF-72, RLF-121, SRL-75, SRL-290, SRL-1117, SRL-1287, ERL-25	Cerilla, popote	2	Medicinal = 5	0.0036	0.2317	0		0.0309	0.6436	0		0		0		Bal, BEA, BN, Iz, Me, Pal, Palm, Sol, TS	0.016987	Ixc	R-W, W	F, G, T, Ur	*in situ*
129	Compositae	*Helenium mexicanum* Kunth	RLF-25, SRL-1116, SRL-1134	Chiche de perro	2		0		0		0	-0.8599	0		0		0		BEA, Palm	0	Ixc	W	G	*in situ*
130	Compositae	*Helianthus annuus* L.	Photo record	Girasol	2	Ornamental = 6	0		0		0		0		0		0		Sol	0.000026	Mex	D	P, Prp	*ex situ*
132	Compositae	*Lactuca sativa* L.	Photo record	Lechuga	1		0		0		0		0.0175		0		0		Sol	0.000026	EAAA	D	P, Prp	*ex situ*
131	Compositae	*Launaea intybacea* (Jacq.) Beauverd	SRL-69	Mostaza	1		0		0		0		0		0		0		Bal, Sol	0	Nat-EAAA	R-W	F	*ex situ*
133	Compositae	*Leucanthemum maximum* (Ramond) DC.	ERL-138	Margarita, margaritón	2	Ornamental = 24, ceremonial = 8	0		0.0095		0		0		0.0417		0		Pal, Sol	0.000102	EAAA	D	P, Prp	*ex situ*
134	Compositae	*Matricaria chamomilla* L.	SRL-175	Manzanilla	1	Medicinal = 55	0		0		0.0868		0		0		0		Sol	0.000051	EAAA	D, R-W	E, P, Prp, T, Ti	*ex situ*
135	Compositae	*Melampodium divaricatum* (Rich. ex Rich.) DC.	RLF-205	Chimalacate	2		0	-0.7656	0	-1.4404	0		0		0		0		Iz	0	Ixc	W	F	*in situ*
136	Compositae	*Melampodium longifolium* Cerv. ex Cav.	SRL-129, RLF-261		1		0		0	-1.5115	0		0		0		0		BEA, Pal	0	Ixc	W	T, Ur	*in situ*
137	Compositae	*Melampodium* sp.	RLF-220		1		0		0	-2.1063	0		0		0		0		Iz	0	Ixc	W		
138	Compositae	*Montanoa tomentosa* Cerv.	RLF-300, SRL-2	Oaxaqueña	1		0		0		0	-1.0367	0		0		0		Iz, Sol	0.000728	Ixc	R-W, W	G	*in situ*
139	Compositae	*Montanoa* sp.	RLF-299		1		0	-1.0354	0		0		0		0		0		Iz	0.001532	Ixc	W	F	*in situ*
150	Compositae	*Neurolaena lobata* (L.) R.Br. ex Cass.	SRL-198	Naranjillo	2		0		0		0	-0.8599	0		0	-1.4144	0		VR	0	Ixc	W	G	*in situ*
140	Compositae	*Parthenium bipinnatifidum* (Ortega) Rollins	ERL-9, RLF-87, RLF-178, SRL-34, SRL-82, SRL-445, SRL-1325	Hierba cenizo	2		0	-0.2194	0		0	-0.507	0		0		0		Sol	0.000026	Ixc	R-W	F, G, T, Ur	*in situ*
141	Compositae	*Parthenium tomentosum* DC.	SRL-1213, SRL-1375	Palo prieto	2		0		0		0	-0.86	0		0		0	-1.7316	CaCe, SB	0	Ixc	W	G	*in situ*
142	Compositae	*Parthenium* sp.	RLF-198				0		0		0		0		0		0		Iz	0	Ixc	W		
143	Compositae	*Perymenium discolor* Schrad.	SRL-277, SRL-1266		1		0	-0.2154	0		0		0		0		0		BEA, BEC, BN, Me, Palm, Paz, TS	0.017574	Ixc	W	F, T, Ur	*in situ*
144	Compositae	*Perymenium mendezii var. angustifolium* (Brandegee) J.J.Fay	RLF-110, SRL-351	Cahual delgado	1		0	-1.0332	0		0		0		0		0		Me	0.001615	Ixc	W	F	*in situ*
145	Compositae	*Perymenium* sp.	RLF-251	Cahual	2		0	-0.6097	0		0	-0.8011	0		0		0		Iz	0	Ixc	W	F, G	*in situ*
146	Compositae	*Philactis zinnioides* Schrad.	RLF-322				0		0		0		0		0		0		Iz	0	Ixc	W		
147	Compositae	*Pinaropappus roseus* (Less.) Less.	RJS-8, SRL-407, SRL-1526	Chipule	1		0		0		0.0119	-0.8163	0		0		0		Bal, BG, Iz, Paz	0.002255	Ixc	W	G	*in situ*
148	Compositae	*Piqueria trinervia* Cav.	RLF-8		2		0	-0.6097	0		0	-0.8011	0		0		0		BEA	0	Ixc	W	F, G	*in situ*
151	Compositae	*Porophyllum linaria* (Cav.) DC.	RLF-18, SRL-158, SRL-357, SRL-1150, ERL-141	Pepitza	4	Edible = 95	0		0.0098	2.0349	0	3.1943	0.0784	2.8958	0		0		BEA, BN, Me, Palm, Paz, Sol, TS	0.011119	Ixc	R-W, W	G, P, Prp, T, Ti	*ex situ, in situ*
153	Compositae	*Porophyllum punctatum* (Mill.) S.F.Blake	SRL-207	Papaloquelite	1		0		0		0		0		0		0		Sol	0	TCV	W	P, Ti	*ex situ*
152	Compositae	*Porophyllum ruderale* subsp. *macrocephalum* (DC.) R.R.Johnson	RLF-318, SRL-1539	Papaloquelite	2	Edible = 90	0		0		0		0.1613	3.3603	0		0		Me, Sol	0.000625	Ixc	W	E, G, P, Prp, T, Ti	*ex situ, in situ*
154	Compositae	*Psacalium amplifolium* (DC.) H.Rob. & Brettell	RLF-39, SRL-266				0		0		0		0		0		0		BEA, BEC	0.002022	Ixc	W		
155	Compositae	*Psacalium paucicapitatum* (B.L.Rob. & Greenm.) H.Rob. & Brettell	RLF-193, SRL-1159	Hierba de camote de venado	1		0		0		0.0069	1.6353	0		0		0		BEA, Iz	0.001101	Ixc	W	G, P, Ti	*ex situ, in situ*
156	Compositae	*Psacalium* sp.	RLF-40	Malangar chico, hierba de cruz			0		0		0		0		0		0		BEA	0	Ixc	W		
157	Compositae	*Roldana oaxacana* (Hemsl.) H.Rob. & Brettell	SRL-1411				0		0		0		0		0		0		BEA	0	Ixc	W		
189	Compositae	*Roldana ehrenbergiana* (Klatt) H.Rob. & Brettell	SRL-1152	Hierba de perro	2		0		0		0	-0.8599	0		0		0		BEA	0	Ixc	W	G	*in situ*
158	Compositae	*Sanvitalia procumbens* Lam.	RLF-42, SRL-12, SRL-1179	Ojo de gallo	2		0	-0.1373	0		0	-0.4562	0		0		0		Me, Palm, Sol, TS	0.003088	Ixc	R-W, W	F, G, T, Ur	*in situ*
149	Compositae	*Senecio praecox* (Cav.) DC.	ERL-191, SRL-1487	Consuelda, pata de león	3	Ornamental = 12	0		0.0059	0.9165	0.0093	2.0335	0		0		0		Me, Sol	0.000051	Ixc	W	G, P, Ti	*ex situ, in situ*
159	Compositae	*Simsia lagascaeformis* A.Gray	RLF-310, RLF-297, SRL-28, SRL-37	Cahual de burro, cahual	2	Fodder = 80, ornamental = 35	0.1501	0.2470	0	0.235	0		0		0		0		Palm, Sol, TS	0.015309	Ixc	W	F, G, T, Ur	*in situ*
160	Compositae	*Simsia sanguinea* A.Gray	RLF-55, SRL-112		1		0	-1.0765	0		0		0		0		0		BEA, BN	0	Ixc	W	F	*in situ*
161	Compositae	*Simsia* sp.	RLF-80	Cahual chiquito	1		0	-1.0765	0		0		0		0		0		BEA	0	Ixc	W	F	*in situ*
162	Compositae	*Sonchus oleraceus* (L.) L.	ERL-10, SRL-1126	Chicoria	1		0		0		0		0		0		0		Sol	0.000102	Nat-EAAA	R-W	G, T, Ur	*ex situ*
168	Compositae	*Stevia caracasana* DC.	RLF-211, SRL-1289, SRL-1293, SRL-1402		2	Ceremonial = 8	0		0	-1.201	0		0		0	-1.1394	0		Iz, Pal, Palm	0	Ixc	W	G, T	*in situ*
163	Compositae	*Stevia lucida* Lag.	SRL-332, SRL-339	Chamalacate	2		0	-0.2391	0		0		0		0	-1.1086	0		BN, Iz, Me, Palm, TS	0.005100	Ixc	R-W, W	F, T, Ur	*in situ*
164	Compositae	*Stevia serrata* Cav.	SRL-298				0		0		0		0		0		0		Paz	0.000463	Ixc	W		
165	Compositae	*Stevia* sp.	RLF-2, SRL-282, SRL-288, SRL-313				0		0		0		0		0		0		BEA, BN	0.002541	Ixc	W		
166	Compositae	*Stevia* sp.	RLF-170, RLF-183, SRL-32, SRL-97, SRL-1281	Cahual delgado	2		0	-0.2980	0		0	-0.5662	0		0		0		BN, Pal, Sol, TS	0	Ixc	W	F, G, T	*in situ*
167	Compositae	*Stevia* sp.	RLF-276	Cahual prieto	1		0		0		0	-1.0487	0		0		0		BEA, Pal	0	Ixc	W	G	*in situ*
169	Compositae	*Stevia* sp.	SRL-1262		2		0		0		0	-0.8599	0		0	-1.4144	0		Me	0	Ixc	W	G	*in situ*
170	Compositae	*Stevia* sp.	SRL-1295		2		0	-0.2980	0		0	-0.5662	0		0		0		Pal	0	Ixc	W	F, G	*in situ*
96	Compositae	*Symphyotrichum novi-belgii* (L.) G.L.Nesom	SRL-56, ERL-66, ERL-86, ERL-154, ERL-225	Esther	2	Ornamental = 47, ceremonial = 14	0		0.0017		0		0		0		0		Sol	0.000205	AC	R-W, W	E, P, Prp, T, Ti	*ex situ*
171	Compositae	*Tagetes erecta* L.	ERL-12, ERL-62, ERL-117, ERL-118, ERL-134, ERL-149, ERL-151, ERL-152, ERL-159, SRL-7, SRL-408, SRL-1142	Cempasuchí	3	Ornamental = 71, ceremonial = 99	0		0.0189		0.0026		0		0.3832		0		Sol, TS	0.000307	TCV	D	E, P, Prp, T, Ti	*ex situ*
172	Compositae	*Tagetes lucida* Cav.	RLF-3, SRL-377, SRL-1232, SRL-1426	Pericón	4	Ceremonial = 50	0		0.0241	-0.1211	0.0523	0.4295	0.0053	-0.8056	0.0069	-0.4661	0		BEA, Paz	0.003298	Ixc	W	G	*in situ*
173	Compositae	*Tagetes lunulata* Ortega	ERL-137	Cempasuchí chiquito	3	Ornamental = 29, ceremonial = 40	0.0027	1.8836	0	1.0404	0		0		0.0069	0.6516	0		Sol	0.000128	Ixc	R-W, W	E, F, P, Prp, T	*in situ*
174	Compositae	*Tanacetum parthenium* (L.) Sch.Bip.	ERL-77, ERL-153, ERL-178, ERL-202, SRL-64	Santa María	3	Ornamental = 53, ceremonial = 10	0		0		0.0646		0		0		0		Sol	0.000230	EAAA	W	E, P, Prp, Ti	*ex situ*
175	Compositae	*Taraxacum campylodes* G.E.Haglund	ERL-106, SRL-89	Achicoria	3	Ornamental = 18	0		0		0.0046		0.0033		0		0		Sol	0.000077	Nat-EAAA	R-W	G, T, Ur	*ex situ*
176	Compositae	*Tithonia rotundifolia* (Mill.) S.F.Blake	ERL-1, ERL-42, ERL-76, ERL-157, ERL-169	Cahual rojo	3	Ornamental = 65, ceremonial = 10	0		0.0062		0		0		0		0		Sol	0.000281	TCV	W	E, F, P, Prp	*ex situ*
177	Compositae	*Tithonia tubaeformis* (Jacq.) Cass.	RLF-177, SRL-126; RLF-57, SRL-1144	Cahual	3	Fodder = 80, ornamental = 41	0.1501	0.1872	0.002	0.3403	0		0		0		0.0026	-1.1591	BEA, Iz, Me, Sol, TS	0.001488	Ixc	R-W, W	F, G, T, Ur	*in situ*
178	Compositae	*Tridax coronopifolia* (Kunth) Hemsl.	SRL-104		1		0		0		0	-1.0487	0		0		0		BN	0	Ixc	R-W, W	G	*in situ*
179	Compositae	*Verbesina gracilipes* B.L.Rob.	SRL-392	Chimalacate	2		0	-0.6097	0		0		0		0		0.0029	-1.668	BEA	0	Ixc	W	F, G	*in situ*
180	Compositae	*Vernonia karvinskiana* DC.	RLF-187, RLF-210		1		0		0	-2.1063	0		0		0		0		Iz	0	Ixc	W		
181	Compositae	*Viguiera cordata* (Hook. & Arn.) D'Arcy	SRL-95	Cahual menudito, cahual prieto	1		0	-1.0765	0		0		0		0		0		Bal	0	Ixc	R-W, W	F	*in situ*
182	Compositae	*Viguiera dentata* (Cav.) Spreng.	RLF-227, SRL-164, SRL-1277, SRL-1302	Chimalacate	5		0	0.7128	0		0	0.0591	0	-0.4176	0	-0.4375	0		BEA, BEC, BG, Iz, Me, Pal, Sol, TS	0	Ixc	R-W, W	F, G, T, Ur	*in situ*
183	Compositae	*Viguiera grammatoglossa* DC.	RLF-233, RLF-298, SRL-347, SRL-1286	Cahual prieto	2		0	-0.2201	0		0	-0.5074	0		0		0		BG, Iz, Me, Pal, Palm, TS	0	Ixc	R-W, W	F, G, T, Ur	*in situ*
184	Compositae	*Viguiera purpusii* Brandegee	RLF-248	Cahual cimarrón	1		0	-1.0765	0		0		0		0		0		Iz	0	Ixc	R-W, W	F	*in situ*
185	Compositae	*Zaluzania* sp.	RLF-238, SRL-1387	Cahualito	1		0	-1.0765	0		0		0		0		0		Iz, SB	0	Ixc	W	F	*in situ*
186	Compositae	*Zinnia elegans* L.	ERL-156	Gallito	2	Ornamental = 6	0		0		0		0		0		0		Sol	0.000026	Mex	D	P, Prp	*ex situ*
187	Compositae	*Zinnia peruviana* (L.) L.	RLF-12, RLF-234, SRL-367, SRL-1173, SRL-1261, SRL-1317	Gallito	3		0	0.3455	0		0	-0.161	0		0	-0.6963	0		BEA, BN, Iz, Me, Palm, TS	0.009492	Ixc	R-W, W	F, G, T, Ur	*in situ*
188	Compositae		SRL-1421				0		0		0		0		0		0		BEA, BEM	0	Ixc	W		
191	Compositae		SRL-1465				0		0		0		0		0		0		Me	0	Ixc	W		
192	Compositae		SRL-1422				0		0		0		0		0		0		BEM	0	Ixc	W		
193	Compositae		SRL-1527				0		0		0		0		0		0		BG	0	Ixc	W		
194	Compositae		SRL-1214	Jazmincillo, cahual blanco	1		0		0		0		0		0	-1.6375	0		SB	0	Ixc	W	G	*in situ*
195	Compositae		SRL-1236		1		0	-1.0765	0		0		0		0		0		BEA	0	Ixc	W	F	*in situ*
196	Compositae		SRL-1442, SRL-1530				0		0		0		0		0		0		BG, VR	0	Ixc	W		
197	Compositae		SRL-1372		1		0		0		0	-1.0487	0		0		0		CaCe	0	Ixc	W	G	*in situ*
198	Compositae		SRL-1445				0		0		0		0		0		0		VR	0	Ixc	W		
199	Compositae		SRL-1355				0		0		0		0		0		0		CaCe	0	Ixc	W		
200	Compositae		SRL-1381	Cahual de hembra			0		0		0		0		0		0		SB	0	Ixc	W		
201	Compositae		SRL-1407		1		0	-1.0765	0		0		0		0		0		BEA	0	Ixc	W	F	*in situ*
202	Compositae		SRL-1224	Cahual	1		0		0	-2.1063	0		0		0		0		SB	0	Ixc	W		
203	Compositae		SRL-1205				0		0		0		0		0		0		Paz	0	Ixc	W		
204	Compositae		SRL-1335				0		0		0		0		0		0		CaCe	0	Ixc	W		
205	Compositae		SRL-1360				0		0		0		0		0		0		CaCe	0	Ixc	W		
206	Compositae		SRL-1337				0		0		0		0		0		0		CaCe	0	Ixc	W		
207	Compositae		SRL-1383				0		0		0		0		0		0		SB	0	Ixc	W		
208	Compositae		SRL-1377				0		0		0		0		0		0		Me	0	Ixc	W		
209	Compositae		ERL-121, SRL-1275	Cahual prieto	1		0		0		0	-0.8133	0		0		0		Pal, Sol, VR	0.000026	Ixc	W	G, T	*in situ*
210	Compositae		SRL-1478	Hierba de ángel, oaxaqueña	1	Medicinal = 15	0		0		0	0.0384	0		0		0		BEA	0	Ixc	W	G	*in situ*
211	Compositae		SRL-1339	Cempasuchí de molito de campo	1		0		0		0	-1.0487	0		0		0		CaCe	0	Ixc	W	G	*in situ*
305	Convolvulaceae	*Cuscuta* sp.	RLF-264, RLF-315, SRL-447				0		0		0		0		0		0		BEA, BN, Sol, TS	0.000758	Ixc	R-W	Ur	*in situ*
306	Convolvulaceae	*Cuscuta* sp.	SRL-1540, SRL-1545				0		0		0		0		0		0		Sol	0	Ixc	R-W	Ur	*in situ*
307	Convolvulaceae	*Dichondra argentea* Humb. & Bonpl. ex Wild.	RLF-71, SRL-134, SRL-167	Orejita de ratón	1		0		0		0	-0.7399	0		0		0		BEA, BEC, BN, Me, Palm	0.018603	Ixc	W	G	*in situ*
309	Convolvulaceae	*Ipomoea conzattii* Greenm.	SRL-1491, SRL-1510	Jícama de cerro	2		0	-0.6097	0		0		0.0042	-1.1374	0		0		CaMy, Me	0	Ixc	W	F, G	*in situ*
310	Convolvulaceae	*Ipomoea elongata* Choisy	RLF-130, RLF-192, SRL-327, SRL-1203	Manto de la virgen del campo	1		0		0	-2.1063	0		0		0		0		BEA, Iz, Paz	0	Ixc	R-W, W		
311	Convolvulaceae	*Ipomoea pauciflora* M.Martens & Galeotti	SRL-1366				0		0		0		0		0		0		CaCe	0	Ixc	W		
308	Convolvulaceae	*Ipomoea aff. populina* House	SRL-1306	Jícama	2		0	-0.6097	0		0		0.0042	-1.1374	0		0		Me	0	Ixc	W	F, G	*in situ*
312	Convolvulaceae	*Ipomoea purpurea* (L.) Roth	ERL-14, RLF-44, RLF-45, SRL-145, SRL-448	Quiebra platos	1		0		0		0	-0.7546	0		0		0		BEA, Me, Paz, Sol, TS	0.000026	Ixc	R-W	G, T, Ur	*in situ*
313	Convolvulaceae	*Ipomoea ternifolia* Cav.	SRL-1363		1		0	-1.0765	0		0		0		0		0		CaCe	0	Ixc	W	F	*in situ*
314	Convolvulaceae	*Ipomoea tricolor* Cav	Photo record	Manto de la Virgen	1	Ornamental = 12	0		0.0147		0		0		0		0		Sol	0.000051	TCV	W	Prp	*ex situ*
315	Crassulaceae	*Aeonium arboreum* Webb & Berthel.	Photo record		1	Ornamental = 6	0		0		0		0		0		0		Sol	0.000026	EAAA	W	P, Prp	*ex situ*
316	Crassulaceae	*Bryophyllum delagoense* (Eckl. & Zeyh.) Druce	ERL-3, SRL-61	Viborita	2	Ornamental = 12	0		0		0		0		0		0		Sol	0.000051	EAAA	W	P, Prp, T	*ex situ*
317	Crassulaceae	*Echeveria gigantea* Rose & Purpus	SRL-1313	Siempreviva grande, lengua de vaca, oreja de toro	2	Ornamental = 18	0		0.0107	0.9419	0.0025	1.7348	0		0		0		MR, Sol	0.000077	Ixc	W	G, P, Ti	*ex situ, in situ*
318	Crassulaceae	*Echeveria nodulosa* (Baker) Otto	SRL-356, SRL-1187, SRL-1255, SRL-1436	Siempreviva chiquita	2		0		0.0033	0.2914	0	1.7058	0		0		0		BEA, Me, Iz, Palm, Sol	0.000823	Ixc	W	G, P, Ti	*ex situ, in situ*
319	Crassulaceae	*Echeveria pulvinata* Rose	Photo record	Siempreviva	2	Ornamental = 6, ceremonial = 1	0		0		0		0		0		0		Sol	0.000026	TCV	W	P, Prp	*ex situ*
320	Crassulaceae	*Echeveria* sp.	Photo record	Siempreviva	1	Ornamental = 6	0		0	-0.0219	0		0		0		0		NE, Sol	0.000026	Ixc	W	P, Ti	*ex situ, in situ*
321	Crassulaceae	*Echeveria* sp.	Photo record	Siempreviva	1		0		0	-0.1783	0		0		0		0		Sol, VN	0	Ixc	W	P, Ti	*ex situ, in situ*
322	Crassulaceae	*Kalanchoe blossfeldiana* Poelln.	ERL-96	Juanita	1	Ornamental = 6	0		0		0		0		0		0		Sol	0.000026	EAAA	W	P, Prp	*ex situ*
323	Crassulaceae	*Kalanchoe* sp.	ERL-26, ERL-183, SRL-1552	Oreja de elefante	2	Ornamental = 41, ceremonial = 14	0		0		0		0		0		0		Sol	0.000179	EAAA	W	P, Prp	*ex situ*
324	Crassulaceae	*Sedum allantoides* Rose	ERL-67, ERL-192	Dedito de Dios	2	Ornamental = 18	0		0		0		0		0		0		Sol	0.000077	TCV	W	P, Prp	*ex situ*
325	Crassulaceae	*Sedum dendroideum* Moc. & Sessé ex DC.	SRL-77, SRL-195, ERL-97, ERL-174	Siempreviva	3	Ornamental = 29, ceremonial = 14	0		0.0272	2.4485	0.0056	2.5616	0		0.0069	1.3626	0		NE, Sol	0.000128	Ixc	W	P, Prp, Ti	*ex situ, in situ*
326	Crassulaceae	*Sedum hemsleyanum* Rose	SRL-1311				0		0		0		0		0		0		MR	0	Ixc	W		
327	Crassulaceae	*Sedum liebmannianum* Hemsl.	ERL-57, ERL-68, SRL-147, SRL-373, SRL-1174	Siempreviva chiquita	2	Ornamental = 6	0	3.4262	0.0037	0.9638	0		0		0		0		BEA, BN, Me, Palm, Sol	0.000026	Ixc	W	F, P, Prp, Ti	*ex situ, in situ*
328	Crassulaceae	*Sedum stahlii* Solms	SRL-1554				0		0		0		0		0		0		Me	0	Ixc	W		
329	Crassulaceae	*Sedum palmeri* S.Watson	Photo record	Siempreviva	1	Ornamental = 6	0		0		0		0		0		0		Sol	0.000026	Mex	W	P, Prp	*ex situ*
330	Crassulaceae	*Sedum potosinum* Rose	Photo record		1	Ornamental = 12	0		0		0		0		0		0		Sol	0.000051	Mex	W	P, Prp	*ex situ*
331	Crassulaceae	*Villadia albiflora* (Hemsl.) Rose	SRL-1310	Borreguito			0		0		0		0		0		0		MR, Me	0	Ixc	W		
332	Crassulaceae	*Villadia guatemalensis* Rose	ERL-45, SRL-1312, SRL-1484	Colita de borrego	2	Ornamental = 6	0	3.4262	0	0.8632	0		0		0		0		Me, MR, Sol	0.000026	Ixc	W	F, P, Prp, Ti	*ex situ, in situ*
336	Cucurbitaceae	*Cucumis melo* L.	Photo record	Melón	1		0		0		0		0		0		0		Sol	0	EAAA	D	P, T	*ex situ*
333	Cucurbitaceae	*Cucurbita ficifolia* Bouché	Photo record	Chilacayota	1	Edible = 100	0		0		0		0		0		0		Sol	0.000256	Mex	D	E, P, Prp, Ti	*ex situ*
334	Cucurbitaceae	*Cucurbita pedatifolia* L.H.Bailey	ERL-120, RLF-268, SRL-1135	Calabacita amarga	3		0	0.0916	0		0	-0.3182	0		0		0		Bal, Pal, Sol	0.000026	Ixc	W	F, G, T, Ur	*in situ*
335	Cucurbitaceae	*Cucurbita pepo* L.	SRL-184	Calabaza	2	Edible = 100	0		0		0		0.0411		0		0		Sol, TS	0.000256	TCV	D	E, P, Prp, Ti	*ex situ*
337	Cucurbitaceae	*Cyclanthera dissecta* (Torr. & A.Gray) Arn.	SRL-151	Chayotito	2		0	-0.2201	0		0	-0.5074	0		0		0		Me, TS	0	Ixc	R-W	F, G, T, Ur	*in situ*
338	Cucurbitaceae	*Schizocarpum filiforme* Schrad.	SRL-1260	Chayotito	2		0	-0.2201	0		0	-0.5074	0		0		0		Sol, TS	0	Ixc	W	F, G, T, Ur	*in situ*
339	Cucurbitaceae	*Sechium edule* subsp. *edule* (Jacq.) Sw.	ERL-56, ERL-215	Chayote	1	Edible = 100	0		0		0		0.0128		0		0		Sol	0.000179	TCV	D	P, Prp	*ex situ*
340	Cucurbitaceae	*Sicyos laciniatus* L.	ERL-100, RLF-90, SRL-14	Chayotillo, pegajosa	2	Fodder = 40	0	-0.0182	0		0	-0.4506	0		0		0		Sol, TS	0.003422	Ixc	R-W	F, G, T, Ur	*in situ*
342	Cupressaceae	*Cupressus sempervirens* L.	Photo record	Ciprés	1	Ornamental = 24	0		0.0294		0		0		0		0		Sol	0.000102	EAAA	W	P, Ti	*ex situ*
341	Cupressaceae	*Cupressus lusitanica* var. *benthamii* (Endl.) Carrière	RLF-129, SRL-36	Nebro fino	3	Ornamental = 6	0		0		0		0		0		0		Sol	0.000026	TCV	W	P, Ti	*ex situ*
343	Cupressaceae	*Juniperus flaccida* Schltdl.	ERL-187, RLF-126, RLF-134, SRL-123, SRL-412, SRL-1119	Nebro	8	Ornamental = 35, firewood = 100	0.0054	5.2489	0.0147	4.8804	0	3.0378	0		0	2.7845	0.14	2.9782	BEA, BEC, BG, BN, Iz, Me, Pal, Palm, Sol, TS	0.085151	Ixc	W	F, G, P, T, Ti	*in situ*
344	Cupressaceae	*Taxodium huegelii* C.Lawson	SRL-210, SRL-434, SRL-1294	Sabino	5	Ornamental = 6	0		0	2.3689	0		0		0	2.3325	0		BG, Pal, Palm, Sol	0.018054	Ixc	W	G, P, Prp, T, Ti	*ex situ, in situ*
345	Cupressaceae	*Thuja occidentalis* L.	ERL-122	Tuja	1	Ornamental = 6	0		0		0		0		0		0		Sol	0.000026	AC	W	P, Ti	*ex situ*
347	Cyperaceae	*Bulbostylis juncoides* (Vahl) Kük. ex Herter	SRL-310	Pasto	1		0	-0.4243	0		0		0		0		0		Me, Palm, Paz, TS	0.009787	Ixc	R-W, W	F, T, Ur	*in situ*
348	Cyperaceae	*Carex* sp.	RLF-133	Pasto	2		0	-0.6097	0		0	-0.8011	0		0		0		Me	0	Ixc	W	F, G	*in situ*
349	Cyperaceae	*Cyperus aggregatus* (Willd.) Endl.	SRL-382	Pasto	1		0	-1.0538	0		0		0		0		0		Paz	0.000846	Ixc	W	F	*in situ*
351	Cyperaceae	*Cyperus spectabilis* Link	RLF-334	Pasto			0		0		0		0		0		0		Iz	0	Ixc	W		
352	Cyperaceae	*Eleocharis acicularis* (L.) Roem. & Schult.	RLF-138	Pasto de arroyo	1		0		0		0	-1.0487	0		0		0		VR	0	Ixc	W	G	*in situ*
353	Cyperaceae	*Eleocharis montevidensis* Kunth	SRL-197	Pasto de arroyo	1		0		0		0		0		0		0		VR	0	Ixc	W	G	*in situ*
346	Cyperaceae	*Fimbristylis mexicana* Palla	SRL-304	Pasto	1		0	-0.2720	0		0		0		0		0		Me, Palm, Paz, TS	0.015465	Ixc	W	F, T, Ur	*in situ*
354	Cyperaceae	*Fuirena simplex* Vahl	SRL-431	Pasto	1		0	-1.0765	0		0		0		0		0		BG, Pal	0	Ixc	W	F	*in situ*
350	Cyperaceae	*Pycreus niger* (Ruiz & Pav.) Cufod.	RLF-144	Pasto	1		0	-1.0765	0		0		0		0		0		Paz	0	Ixc	W	F	*in situ*
355	Cyperaceae	*Rhynchospora* sp.	RLF-145	Pasto fino	1		0	-1.0765	0		0		0		0		0		Paz	0	Ixc	W	F	*in situ*
356	Ebenaceae	*Diospyros oaxacana* Standl.	SRL-1446	Zapotito	2		0	-0.6097	0		0		0	-1.1696	0		0		VR	0	Ixc	W	F, G	*in situ*
357	Equisetaceae	*Equisetum* sp.	SRL-422				0		0		0		0		0		0		Bg	0	Ixc	W		
358	Ericaceae	*Arbutus xalapensis* Kunth	ERL-172, RLF-124, RLF-279, SRL-1477	Madroño, ollita	4	Ceremonial = 14, firewood = 100	0		0		0	-0.1056	0		0	-0.4749	0.12	-0.2619	BEA, BEC, BEM, BN, Me, TS	0.008534	Ixc	W	G, T	*in situ*
359	Ericaceae	*Comarostaphylis polifolia* (Kunth) Zucc. ex Klotzsch	RLF-118, SRL-130, SRL-250, SRL-1495	Palo prieto	3	Firewood = 100	0		0		0		0		0		0.025	-0.6676	BEA, BEC, BEM, BN, Me, Palm, TS	0.010056	Ixc	W	G, T	*in situ*
360	Euphorbiaceae	*Acalypha* aff. *purpurascens* Kunth	RLF-189, SRL-256				0		0		0		0		0		0		BEA, Iz, Pal	0.001362	Ixc	W	T, Ur	*in situ*
361	Euphorbiaceae	*Bernardia* sp.	SRL-1386				0		0		0		0		0		0		CaCe	0	Ixc	W		
362	Euphorbiaceae	*Cnidosculus tehuacanensis* Breckon	Photo record	Mala mujer	1		0		0		0.0043	-0.9341	0		0		0		Iz, Palm	0.002686	Ixc	W	G	*in situ*
363	Euphorbiaceae	*Croton* sp.	SRL-441				0		0		0		0		0		0		Iz	0	Ixc	W		
364	Euphorbiaceae	*Croton* sp.	SRL-1444				0		0		0		0		0		0		VR	0	Ixc	W		
365	Euphorbiaceae	*Euphorbia colletioides* Benth.	SRL-1359		1		0	-1.0765	0		0		0		0		0		CaCe	0	Ixc	W	F	*in situ*
366	Euphorbiaceae	*Euphorbia cyathophora* Murray	SRL-1369				0		0		0		0		0		0		CaCe	0	Ixc	W		
367	Euphorbiaceae	*Euphorbia cymbifera* (Schltdl.) V.W.Steinm.	SRL-1500				0		0		0		0		0		0		CaCe	0	Ixc	W		
368	Euphorbiaceae	*Euphorbia cyri* V.W.Steinm.	SRL-1128	Cordobán	2	Ornamental = 12	0		0		0		0		0		0		Sol	0.000051	TCV	W	P, Prp	*ex situ*
369	Euphorbiaceae	*Euphorbia dentata* Michx.	RLF-51, SRL-102, SRL-299, SRL-376	Lechillo, limil	1		0.0025	-0.1758	0		0		0		0		0		BEA, BEC, BN, Iz, Me, Palm, Paz, TS	0.019153	Ixc	W	F, T, Ur	*in situ*
370	Euphorbiaceae	*Euphorbia dioeca* Kunth	ERL-107, RLF-7, SRL-359	Celedonia	1		0		0		0	-0.7546	0		0		0		BEA, Sol	0.000026	Ixc	W	G, T, Ur	*in situ*
371	Euphorbiaceae	*Euphorbia graminea* Jacq.	RLF-288, RLF-311, SRL-317		1		0		0		0		0		0		0		BEA, BEC, BG, Iz, Palm, Sol, TS	0.010247	Ixc	W	G, T, Ur	*in situ*
372	Euphorbiaceae	*Euphorbia lactea* Haw.	Photo record		1		0		0		0		0		0		0		Sol	0	EAAA	W	P, Ti	*ex situ*
373	Euphorbiaceae	*Euphorbia macropus* (Klotzsch & Garcke) Boiss.	SRL-1120	Hierba de chicle	2		0		0		0	-0.8599	0	-1.2217	0		0		Palm	0	Ixc	W	G	*in situ*
374	Euphorbiaceae	*Euphorbia pulcherrima* Willd. ex Klotzsch	Photo record	Noche buena	2	Ornamental = 47, ceremonial = 11	0		0.1246		0		0		0.0444		0		Sol	0.000205	Mex	D	P, Prp	*ex situ*
375	Euphorbiaceae	*Euphorbia rossiana* Pax	SRL-1450				0		0		0		0		0		0		CaCe, Me	0	Ixc	W		
376	Euphorbiaceae	*Euphorbia* sp.	RLF-141	Mastrancito			0		0		0		0		0		0		VR	0	Ixc	W		
377	Euphorbiaceae	*Euphorbia* sp.	RLF-301, SRL-254				0		0		0		0		0		0		BEA, Iz	0.002724	Ixc	W		
378	Euphorbiaceae	*Euphorbia* sp.	RLF-119, RLF-152, RLF-167, SRL-283				0		0		0		0		0		0		BN, Me	0.001886	Ixc	W		
379	Euphorbiaceae	*Jatropha neopauciflora* Pax	SRL-1357	Sangre de grado, aceitillo	2		0	-0.6097	0		0	-0.8011	0		0		0		CaCe	0	Ixc	W	F, G	*in situ*
380	Euphorbiaceae	*Ricinus communis* L.	ERL-116, ERL-144, ERL-145, ERL-243, SRL-23, SRL-1129	Gría	5		0		0		0.0161		0		0		0		Bal, Sol	0.000205	Nat-EAAA	R-W	E, G, P, T, Ur	*ex situ*
381	Euphorbiaceae	*Sebastiania* aff. *pavoniana* (Müll.Arg.) Müll.Arg.	SRL-263	Hierba de venado	1		0	-1.0683	0		0		0		0		0		BEA, BN	0.000305	Ixc	W	F	*in situ*
382	Euphorbiaceae	*Tragia nepetifolia* Cav.	SRL-318				0		0		0		0		0		0		BEA, BEC	0.001155	Ixc	W		
383	Euphorbiaceae		RLF-252				0		0		0		0		0		0		Iz	0	Ixc	W		
384	Fagaceae	*Quercus acutifolia* Née	SRL-1226, SRL-1516	Encino colorado	7	Firewood = 100	0.0153	3.7957	0.0392	2.304	0.0101	2.6129	0		0	2.1047	0.2789	2.3609	BEM	0	Ixc	W	F, G, P, Ti	*ex situ, in situ*
385	Fagaceae	*Quercus castanea* Née	RLF-78, SRL-1233, SRL-1408, SRL-1425, SRL-1431	Encino prieto, encino blanco	7	Firewood = 100	0.0215	1.4099	0.0392	1.4528	0	0.4908	0		0	0.4695	0.1446	0.4208	BEA, BEM, BN, TS	0.018170	Ixc	W	F, G, T	*in situ*
386	Fagaceae	*Quercus conspersa* Benth.	SRL-1156	Encino colorado	7	Firewood = 100	0.0153	0.6176	0.0392	0.7792	0.0101	0.1196	0		0	0.2552	0.2789	0.2097	BEM	0	Ixc	W	F, G, P	*in situ*
393	Fagaceae	*Quercus x dysophylla* Benth.	SRL-1108	Encino de tesmole	3	Firewood = 100	0.0091	0.6263	0.0392	0.5657	0		0		0		0.0099	-0.3662	BEA, Palm, TS	0	Ixc	W	F, G, P,T	*in situ*
387	Fagaceae	*Quercus glaucoides* M. Martens & Galeotti	SRL-1109, SRL-1459, SRL-1486, SRL-1513	Encino chaparro	5	Firewood = 100	0.0161	0.3057	0.0686	1.3213	0		0		0		0.0155	-0.632	Me, Palm	0	Ixc	W	F, G	*in situ*
388	Fagaceae	*Quercus laeta* Liebm.	RLF-68, SRL-143, SRL-253, SRL-385, SRL-1230	Encino prieto, encino amarillo	6	Ornamental = 6, firewood = 100	0.0129	4.1162	0.0392	2.6146	0		0		0	3.5799	0.7699	3.806	BEA, BEC, Pal, Sol	0.003111	Ixc	W	F, G, P, Prp, T	*ex situ, in situ*
389	Fagaceae	*Quercus liebmannii* Oerst. ex Trel.	SRL-1107, SRL-1514	Encino amarillo	8	Fodder = 5, firewood = 100	0.0108	6.7493	0.0392	4.6656	0		0		0	5.4336	0.7699	5.5501	BEA, Me, Palm, TS	0.048434	Ixc	W	F, G, P, Prp, T, Ti	*ex situ, in situ*
390	Fagaceae	*Quercus obtusata* Bonpl.	SRL-1423	Encino prieto	6	Firewood = 100	0.0092	0.9366	0.0392	0.8996	0		0		0	0.0928	0.1446	0.1359	BEM	0	Ixc	W	F, G, P	*in situ*
391	Fagaceae	*Quercus polymorpha* Schltdl. & Cham.	SRL-1503	Encino prieto	5		0	0.6356	0.0392	0.6369	0		0		0	-0.5067	0	-0.8204	BG, Pal	0	Ixc	W	F, G, P	*in situ*
392	Fagaceae	*Quercus urbanii* Trel	RLF-161, SRL-252, SRL-475, SRL-1228	Encino cucharilla	6	Firewood = 100	0.0081	1.9079	0.0392	1.7423	0		0		0	0.9619	0.2136	0.9509	BEA, BEC, TS	0.024545	Ixc	W	F, G, P,T	*in situ*
395	Garryaceae	*Garrya ovata* Benth.	SRL-330, SRL-469	Hierba de ardilla	2	Firewood = 100	0.0323	-0.0578	0		0		0		0		0.0222	-0.8145	Me, TS	0.010266	Ixc	W	F, G, T	*in situ*
396	Geraniaceae	*Geranium* sp.	RLF-278, SRL-136				0		0		0		0		0		0		Pal, Palm	0	Ixc	W	T	*in situ*
397	Geraniaceae	*Pelargonium peltatum* (L.) L'Hér.	Photo record	Geranio, malva rosa	2	Ornamental = 6	0		0		0		0		0		0		Sol	0.000026	EAAA	D	P, Prp	*ex situ*
398	Geraniaceae	*Pelargonium zonale* (L.) L'Hér. ex Aiton	ERL-84, ERL-200	Geranio, malva rosa	2	Ornamental = 88, ceremonial = 43	0		0.0888		0		0		0.0386		0		Sol	0.000384	EAAA	D	P, Prp	*ex situ*
399	Geraniaceae		SRL-81				0		0		0		0		0		0		Sol	0	Ixc	W	T	*in situ*
400	Hydrangeaceae	*Hydrangea macrophylla* (Thunb.) Ser.	Photo record	Hortensia	2		0		0		0		0		0.0093		0		Sol	0	EAAA	D	P, Ti	*ex situ*
404	Hypoxidaceae	*Hypoxis* sp.	RLF-37, SRL-141	Pasto	2		0	-0.5563	0		0		0		0		0		BEA, BEC	0.001991	Ixc	W	F, G	*in situ*
405	Iridaceae	*Gladiolus hortulanus* L.H. Bailey	Photo record	Gladiolo	2	Ornamental = 41, ceremonial = 22	0		0		0		0		0.1512		0		Sol	0.000179	EAAA	D	P, Prp	*ex situ*
406	Iridaceae	*Iris × germanica* L.	SRL-225	Lirio corriente	2	Ornamental = 29	0		0		0		0		0		0		Pal, Sol	0.000128	EAAA	D	P, Prp	*ex situ*
407	Iridaceae	*Neomarica* sp.	Photo record	Lirio	2		0		0		0		0		0		0		Sol	0	AC	D	P, Prp	*ex situ*
408	Iridaceae	*Sisyrinchium tenuifolium* Humb. & Bonpl. ex Willd.	RLF-146, SRL-1548	Hierba de camino corriente	1		0	-0.9652	0		0		0		0		0		BEA, Iz	0.004148	Ixc	W	F	*in situ*
409	Iridaceae	*Tigridia illecebrosa* Cruden	RJS-10	Flor de gamito	2		0	-0.7655	0	-1.4404	0		0		0		0		Me	0	Ixc	W	F	*in situ*
410	Iridaceae	*Tigridia pavonia* (L.f.) DC.	RLF-201		1		0		0	-2.1063	0		0		0		0		Iz	0	Ixc	W		
669	Iteaceae	*Pterostemon rotundifolius* Ramírez	RLF-272, RLF-273, SRL-331	Encino redondo o chaparro	1	Firewood = 100	0		0		0		0		0		0.0036	-1.1501	BEA, BEC, BN, Me, Palm, TS	0.008338	Ixc	W	G, T	*in situ*
411	Juglandaceae	*Juglans regia* L.	ERL-193	Nuez	1		0		0		0		0		0		0		Sol	0.000026	EAAA	D	P, Prp	*ex situ*
412	Krameriaceae	*Krameria cytisoides* Cav.	RLF-97, SRL-251, SRL-1265, SRL-1376	Chayotillo de burro, borreguito	2		0	-0.5482	0		0.004	-0.6981	0		0		0		Me, Palm	0.002292	Ixc	W	F, G	*in situ*
413	Lamiaceae	*Clinopodium mexicanum* (Benth.) Govaerts	RLF-131, RLF-262, SRL-1190, SRL-1280, SRL-1403	Chipito	2	Medicinal = 5	0		0		0.1359	4.2857	0	0.9569	0		0		BEA, Me, Pal, Sol, VR	0.000350	Ixc	W	G, P, Ti	*ex situ, in situ*
414	Lamiaceae	*Hyptis* sp.	RLF-38				0		0		0		0		0		0		BEA	0	Ixc	W		
415	Lamiaceae	*Hyptis* sp.	SRL-209		1		0		0		0	-0.8137	0		0		0		Sol	0	Ixc	W	G, T	*in situ*
416	Lamiaceae	*Leonotis nepetifolia* (L.) R.Br.	SRL-1315		2	Ornamental = 6	0		0		0		0		0		0		Sol	0.000026	Nat-EAAA	R-W	E, P, T, Ur	*ex situ*
417	Lamiaceae	*Marrubium vulgare* L.	ERL-80, RLF-64, SRL-29, SRL-1146	Manrrubio	1	Medicinal = 10	0		0		0.056		0		0		0		Bal, Pal, Sol	0.000205	Nat-EAAA	R-W	G, T, Ur	*ex situ*
418	Lamiaceae	*Mentha × piperita* L.	ERL-19, ERL-61, ERL-95, SRL-70, SRL-1137	Hierba buena	3	Medicinal = 35	0		0		0.0296		0.0263		0		0		Sol	0.000358	EAAA	D	P, Prp	*ex situ*
419	Lamiaceae	*Ocimum basilicum* L.	ERL-186, ERL-211, SRl-176	Albhacar	2	Ornamental = 18	0		0.0294		0.0222		0		0		0		Sol	0.000077	EAAA	D	P, Prp, Ti	*ex situ*
420	Lamiaceae	*Origanum majorana* L.	ERL-15, ERL-53, ERL-85, ERL-142, SRL-73, SRL-206	Orégano	3	Medicinal = 5	0		0		0		0.0183		0		0		Sol	0.000307	EAAA	D	P, Prp	*ex situ*
421	Lamiaceae	*Plectranthus hadiensis* (Forssk.) Schweinf. ex Sprenger	ERL-212		1		0		0		0		0		0		0		Sol	0.000026	EAAA	W	P, Prp	*ex situ*
422	Lamiaceae	*Rosmarinus officinalis* L.	Photo record	Romero cimarrón	2		0		0		0.0093		0		0		0		Sol	0.000026	EAAA	W	P, Prp	*ex situ*
424	Lamiaceae	*Salvia aspera* M.Martens & Galeotti	SRL-345, SRL-1263	Oreganillo	1		0	-0.9559	0		0		0		0		0		BEA, Iz, Me, Palm	0.004494	Ixc	W	F	*in situ*
425	Lamiaceae	*Salvia candicans* M.Martens & Galeotti	SRL-155, SRL-1456		1		0		0		0	-1.0487	0		0		0		Me	0	Ixc	W	G	*in situ*
423	Lamiaceae	*Salvia circinnata* Cav.	RLF-215, SRL-1291		1		0		0		0	-1.0487	0		0		0		Iz, Palm	0	Ixc	W	G	*in situ*
426	Lamiaceae	*Salvia keerlii* Benth.	SRL-155, SRL-1456	Oreganillo			0		0		0		0		0		0		Palm	0	Ixc	W		
427	Lamiaceae	*Salvia oaxacana* Fernald	RLF-232, SRL-1161, SRL-1188	Mirto cimarrón	2		0	-0.6097	0		0	-0.8011	0		0		0		BEA	0	Ixc	W	F, G	*in situ*
428	Lamiaceae	*Salvia pannosa* Fernald	RLF-181				0		0		0		0		0		0		Me, TS	0	Ixc	W	T, Ur	*in situ*
429	Lamiaceae	*Salvia purpurea* Cav.	RLF-1, RLF-194, SRL-116, SRL-273, SRL-1195, SRL-1202, SRL-1397, SRL-1420	Terciopelo	3		0		0		0	-0.5649	0		0.0159	-1.0238	0		BEA, BEC, BN, Iz	0.006393	Ixc	W	G	*in situ*
430	Lamiaceae	*Salvia sessei* Benth.	RLF-33, RLF-195, SRL-1162	Oaxaqueña	1		0		0		0	-1.0487	0		0		0		BEA, BEM	0	Ixc	W	G	*in situ*
431	Lamiaceae	*Salvia thymoides* Benth.	RLF-245, SRL-1469	Oreganillo cenizo	1		0		0		0	-1.0487	0		0		0		Iz, Me	0	Ixc	W	G	*in situ*
432	Lamiaceae	*Salvia tiliifolia* Vahl	ERL-28-ERL-112, RLF-162, SRL-3	Chía	2		0		0		0	-0.5632	0		0		0		Bal, Sol, TS	0.000179	Ixc	W	G, T, Ur	*in situ*
433	Lamiaceae	*Salvia villosa* Fernald	SRL-285				0		0		0		0		0		0		BG, BN	0.001376	Ixc	W		
434	Lamiaceae	*Salvia* sp.	Photo record	Mirto	1		0		0		0.0035	-0.7569	0		0		0		Sol	0	Ixc	W	G, T	*in situ*
435	Lamiaceae	*Salvia* sp.	RLF-20				0		0		0		0		0		0		BEA	0	Ixc	W		
436	Lamiaceae	*Salvia* sp.	RLF-150				0		0		0		0		0		0		Paz	0	Ixc	W		
437	Lamiaceae	*Salvia* sp.	SRL-140	Marrubio macho	1		0		0		0	-1.0487	0		0		0		BEA	0	Ixc	W	G	*in situ*
438	Lamiaceae		SRL-1304		1		0	-1.0765	0		0		0		0		0		Me	0	Ixc	W	F	*in situ*
439	Lamiaceae		SRL-1448				0		0		0		0		0		0		VR	0	Ixc	W		
440	Lauraceae	*Litsea glaucescens* Kunth	SRL-1157, SRL-1515	Laurel	3	Ceremonial = 2	0		0		0		0.0263	-0.2314	0	-0.5565	0		BEA	0	Ixc	W	G, Prp	*in situ*
441	Lauraceae	*Persea americana* Mill.	ERL-52, ERL-65, RLF-106, SRL-432	Aguacate	2	Edible = 100	0		0		0.0013		0.0068		0		0		Pal, Sol, TS	0.000281	TCV	D	P, Prp, T, Ti	*ex situ*
442	Leguminosae	*Acacia cochliacantha* Willd.	SRL-1374	Guaje de espino	1		0	-1.0765	0		0		0		0		0		CaCe	0	Ixc	W	F	*in situ*
443	Leguminosae	*Acacia farnesiana* (L.) Willd.	Photo record	Espino	2		0.0086	-0.2900	0		0		0		0		0.0151	-1.4002	NE, TS	0.000647	Ixc	W	F, G, T	*in situ*
444	Leguminosae	*Acacia pennatula* (Schltdl. & Cham.) Benth.	SRL-1471	Espino	2		0.0076	0.0810	0		0		0		0		0.0151	-1.164	BEA, BEC, Iz, SB, TS	0.014436	Ixc	W	F, G, T	*in situ*
445	Leguminosae	*Acacia schaffneri* (S.Watson) F.J.Herm.	SRL-183,SRL-460	Espino	3		0.0068	0.0056	0		0		0		0		0.0151	-1.2108	Bal, Sol	0	Ixc	W	F, G, T	*in situ*
446	Leguminosae	*Acaciella tequilana* (S.Watson) Britton & Rose	RLF-53	Barba de chivo	1		0	-1.0765	0		0		0		0		0		BEA	0	Ixc	W	F	*in situ*
447	Leguminosae	*Bauhinia* sp.	SRL-160, SRL-1443				0		0		0		0		0		0		BEA, Pal	0	Ixc	W	T	*in situ*
448	Leguminosae	*Calliandra* sp.	SRL-276	Guaje de gamito	2	Edible = 6	0	-0.63	0		0		0	-1.3613	0		0		BEA, BEC, BG, BN, Me	0.005148	Ixc	W	F	*in situ*
449	Leguminosae	*Calliandra* sp.	Photo record	Crin de caballo			0		0		0		0		0		0		Me	0	Ixc	W		
450	Leguminosae	*Calliandropsis nervosus* (Britton & Rose) H.M.Hern. & P.	SRL-1511				0		0		0		0		0		0		CaMy	0	Ixc	W		
451	Leguminosae	*Canavalia villosa* Benth.	RLF-226, SRL-1439		1		0	-1.0765	0		0		0		0		0		BEA, Iz	0	Ixc	W	F	*in situ*
452	Leguminosae	*Cologania broussonetii* (Balb.) DC.	SRL-106		1		0	-1.0765	0		0		0		0		0		BN	0	Ixc	W	F	*in situ*
453	Leguminosae	*Cologania* sp.	RLF-153	Hierba de venado	1		0	-1.0765	0		0		0		0		0		Paz	0	Ixc	W	F	*in situ*
454	Leguminosae	*Cologania* sp.	SRL-324	Lentejilla corriente	1		0	-0.7835	0		0		0		0		0		BEA, BEC, Me	0.010922	Ixc	W	F	*in situ*
455	Leguminosae	*Crotalaria pumila* Ortega	SRL-103, SRL-364		2		0	-0.6097	0		0	-0.8011	0		0		0		BN, Palm	0	Ixc	W	F, G	*in situ*
456	Leguminosae	*Crotalaria* sp.	SRL-13				0		0		0		0		0		0		VR	0	Ixc	W		
457	Leguminosae	*Dalea bicolor* Willd.	SRL-1461				0		0		0		0		0		0		Me	0	Ixc	W		
458	Leguminosae	*Dalea carthagenensis* (Jacq.) J.F.Macbr.	RLF-115, RLF-168, RLF-222, SRL-154, SRL-417, SRL-1185, SRL-1299	Hierba de Obo	2		0	-0.2201	0		0.0096	-0.5388	0		0		0		BG, Iz, Me, TS	0	Ixc	W	F, G, T, Ur	*in situ*
459	Leguminosae	*Dalea hegewischiana* Steud.	SRL-1283				0		0		0		0		0		0		VR	0	Ixc	W		
460	Leguminosae	*Dalea tomentosa* (Cav.) Willd.	RLF-214, SRL-214		2		0	-0.5455	0		0	-0.7614	0		0		0		BN, Iz, Palm	0.002394	Ixc	W	F, G	*in situ*
461	Leguminosae	*Dalea* sp.	RLF-328		1		0	-1.0765	0		0		0		0		0		Iz	0	Ixc	W	F	*in situ*
462	Leguminosae	*Dalea* sp.	SRL-348		1		0		0		0		0	-1.382	0		0		Me	0.000310	Ixc	W	G	*in situ*
463	Leguminosae	*Dalea* sp.	SRL-111, SRL-168				0		0		0		0		0		0		BN, VR	0	Ixc	W		
465	Leguminosae	*Desmanthus virgatus* (L.) Willd.	SRL-368	Guajito de gabito	1		0		0		0		0	-1.3855	0		0		Palm	0	Ixc	W	G	*in situ*
464	Leguminosae	*Desmanthus* sp.	RLF-225	Tepeguaje cimarrón	2		0	-0.6097	0		0		0	-1.1696	0		0		Iz	0	Ixc	W	F, G	*in situ*
466	Leguminosae	*Desmodium axillare* (Sw.) DC.	RLF-74, SRL-101, SRL-286, SRL-425	Lentejilla corriente	1		0	-0.3076	0		0		0		0		0		BEA, BG, BN, Me, Palm, Paz, TS	0.014139	Ixc	W	F, T, Ur	*in situ*
467	Leguminosae	*Desmodium orbiculare* Schltdl.	RLF-216, SRL-1269	Papaloquelite de chivo	1		0.0036	-1.0538	0		0		0		0		0		BEA, Iz, Me	0.000993	Ixc	W	F	*in situ*
468	Leguminosae	*Desmodium subsessile* Schltdl.	RLF-114		1		0	-0.9207	0		0		0		0		0		Me	0	Ixc	R-W, W	F, G	*in situ*
469	Leguminosae	*Erythrina americana* Mill.	ERL-175, SRL-181, SRL-458	Hierba de pipi	5		0.0023		0		0.0025		0.0015		0		0		Sol	0.000026	Mex	W	F, P, Prp	*ex situ*
470	Leguminosae	*Eysenhardtia polystachya* (Ortega) Sarg.	RLF-253, SRL-346, SRL-476	Coatillo	5	Ornamental = 6, firewood = 100	0.0194	0.5698	0	-0.1759	0		0		0		0.0155	-0.3836	BG, Iz, Me, Palm, Sol	0.001263	Ixc	R-W, W	F, G, T	*in situ*
472	Leguminosae	*Harpalyce formosa* DC.	RLF-176, RLF-286, SRL-343	Guaje de caballo	1		0	-1.06	0		0		0		0		0		Iz, Me	0.000616	Ixc	R-W, W	F	*in situ*
473	Leguminosae	*Havardia* sp.	RLF-325				0		0		0		0		0		0		Iz	0	Ixc	R-W, W		
471	Leguminosae	*Hybosema ehrenbergii* (Schltdl.) Harms	RLF-123, SRL-259	Guajillo de chivo	1		0	-0.8214	0		0		0		0		0		BEA, BEC, BG, BN, Me, Palm	0.009509	Ixc	R-W, W	F	*in situ*
474	Leguminosae	*Lens culinaris* Medik.	Photo record	Lenteja	1	Edible = 100	0		0		0		0.0066		0		0		Sol	0.000026	EAAA	D	P, T	*ex situ*
475	Leguminosae	*Leucaena esculenta* (DC.) Benth.	ERL-31, ERL-87, ERL-110, RLF-107, RLF-174, SRL-1167, SRL-1216, SRL-1251, SRL-1343	Guaje colorado, guaje de caballo, guaje de rapia	5	Ornamental = 94, edible = 100, firewood = 100	0.0161		0		0		0.0716		0		0.0134		AA, Sol	0.000409	TCV	D	G, P, Prp, T, Ti	*ex situ*
476	Leguminosae	*Leucaena leucocephala* (Lam.) de Wit	ERL-88, ERL-209	Guaje de la cañada, guaje verde	1	Edible = 47	0		0		0		0		0		0		Sol	0.000205	TCV	D	P, Prp, T, Ti	*ex situ*
478	Leguminosae	*Leucaena* sp.	SRL-1158	Guaje de gamito	1	Edible = 6	0		0		0		0		0	-1.6375	0		BEA	0	Ixc	W	G	*in situ*
477	Leguminosae	*Lupinus leptophyllus* Cham. & Schltdl.	SRL-1410		1		0		0		0		0	-1.3842	0		0		BEA	0	Ixc	W	G	*in situ*
479	Leguminosae	*Macroptilium atropurpureum* (DC.) Urb.	SRL-426		1		0	-1.0539	0		0		0		0		0		BG, Palm, Paz	0.000841	Ixc	W	F	*in situ*
480	Leguminosae	*Macroptilium gibossifolium* (Ortega) A.Delgado	RLF-63, SRL-108		2		0	-0.7428	0		0		0		0		0		BEA, BN, Palm, Paz	0.000846	Ixc	W	F	*in situ*
481	Leguminosae	*Medicago lupulina* L.	SRL-192		1		0		0		0		0		0		0		Sol	0	Nat-EAAA	R-W	F	*ex situ*
482	Leguminosae	*Medicago polymorpha* L.	RLF-69, SRL-15, SRL-1328		1		0		0		0		0		0		0		Sol	0	EAAA	W	F	*ex situ*
483	Leguminosae	*Melilotus indicus* (L.) All.	SRL-88, SRL-120		1		0		0		0		0		0		0		Sol	0	Nat-EAAA	W	F	*ex situ*
484	Leguminosae	*Mimosa lacerata* Rose	RLF-283	Espino	1		0		0		0		0		0		0		Palm, TS	0	Ixc	W	T	*in situ*
485	Leguminosae	*Mimosa* sp.	RLF-85	Garabato, espino	1		0		0		0		0		0		0		Sol	0	Ixc	W	G, T	*in situ*
486	Leguminosae	*Nissolia* sp.	RLF-163				0		0		0		0		0		0		Palm, TS	0	Ixc	W	T, Ur	*in situ*
487	Leguminosae	*Parkinsonia praecox* (Ruiz & Pav.) Hawkins	SRL-1396	Palo verde			0		0		0		0		0		0		CaCe	0	Ixc	W		
488	Leguminosae	*Phaseolus coccineus* L.	ERL-7, ERL-161	Frijol ayocote	2	Edible = 12	0		0		0		0		0		0		Sol	0.000051	TCV	D	P, Prp	*ex situ*
489	Leguminosae	*Phaseolus vulgaris* L.	ERL-8, ERL-47, ERL-48, ERL-49, ERL-139, ERL-160, SRL-9	Frijol de tierra, frijol de milpa, bayo, amarillo, negro, enredador	2	Edible = 100	0.0352		0		0		0.0543		0		0		Sol, TS	0.000230	TCV	D	P, Prp, T	*ex situ*
490	Leguminosae	*Phaseolus* sp.	SRL-144		1		0	-1.0765	0		0		0		0		0		BEA	0	Ixc	W	F	*in situ*
491	Leguminosae	*Phaseolus* sp.	RLF-169				0		0		0		0		0		0		Palm, TS	0	Ixc	W	T, Ur	*in situ*
492	Leguminosae	*Phaseolus* sp.	SRL-1206	Ejote de venado	2		0	-0.6097	0		0		0	-1.1696	0		0		BEA	0	Ixc	W	F, G	*in situ*
493	Leguminosae	*Phaseolus* sp.	SRL-1231		1		0	-1.0765	0		0		0		0		0		BEA	0	Ixc	W	F	*in situ*
494	Leguminosae	*Piscidia grandifolia* (Donn.Sm.) I.M.Johnst.	SRL-1210		2		0		0		0	-0.8599	0		0		0		SB	0	Ixc	W	G	*in situ*
495	Leguminosae	*Pisum sativum* L.	Photo record	Alberjón	1		0		0		0		0.0219		0		0		Sol	0	EAAA	D	P, T	*ex situ*
496	Leguminosae	*Prosopis laevigata* (Willd.) M.C.Johnst.	SRL-1388	Mezquite	5		0	0.4025	0		0.0035	-0.1182	0.0016	-0.6126	0		0	-1.016	Pal, SB, Sol	0.000051	Ixc	W	F, G, T	*in situ*
497	Leguminosae	*Rhynchosia pringlei* Rose	RLF-247, SRL-1440	Hierba de venado	1		0	-1.0765	0		0		0		0		0		BEA, Iz	0	Ixc	W	F	*in situ*
498	Leguminosae	*Rhynchosia senna* Hook.	SRL-284, SRL-366		1		0	-1.0598	0		0		0		0		0		BN, Palm	0.000623	Ixc	W	F	*in situ*
499	Leguminosae	*Senna guatemalensis* (Donn.Sm.) H.S.Irwin & Barneby	RLF-246, RLF-295		3	Ceremonial = 1	0	-0.2593	0		0	-0.588	0		0	-1.1062	0		Iz	0.001468	Ixc	W	F, G	*in situ*
500	Leguminosae	*Senna holwayana* (Rose) H.S.Irwin & Barneby	ERL-223, RLF-75, RLF-230, SRL-1437	Mostaza corriente	2	Ornamental = 6	0	-0.4532	0	-1.0925	0		0		0		0		BEA, Iz, Sol	0.000026	Ixc	R-W, W	F, T	*in situ*
501	Leguminosae	*Teramnus labialis* (L.f.) Spreng.	SRL-396				0		0		0		0		0		0		BEA, Me	0.000420	Ixc	W		
502	Leguminosae	*Trifolium* sp.	SRL-375		2		0		0		0		0		0		0		BEA	0	Nat-Uk	W	F, G	*ex situ*
503	Leguminosae	*Vicia faba* L.	Photo record	Haba	1		0		0		0		0.0243		0		0		Sol, TS	0	EAAA	D	P, Prp	*ex situ*
504	Leguminosae	*Zornia reticulata* Sm.	SRL-300		2		0	-0.5973	0		0	-0.7935	0		0		0		Paz	0.000463	Ixc	W	F, G	*in situ*
505	Leguminosae		RLF-327, SRL-1227	Timbre	5		0.0029	0.3201	0		0		0		0		0.0026	-1.0672	BEA, BEM, Iz	0	Ixc	W	F, G	*in situ*
506	Leguminosae		SRL-1212	Tepeguaje	3		0	-0.4545	0		0	-0.7298	0		0		0		SB	0	Ixc	W	F	*in situ*
507	Leguminosae		SRL-1556				0		0		0		0		0		0		CaCe	0	Ixc	W		
508	Leguminosae		SRL-1538				0		0		0		0		0		0		Me	0	Ixc	W		
509	Leguminosae		SRL-1113	Guaje que come el venado			0		0		0		0		0		0		Me	0	Ixc	W		
510	Leguminosae		RJS-7		1		0	-1.0765	0		0		0		0		0		BEA	0	Ixc	W	F	*in situ*
511	Leguminosae		SRL-1166	Timbre	1		0		0	-0.0825	0		0		0		0		Sol	0	Ixc	W	Ti	*ex situ, in situ*
512	Leguminosae		SRL-1350				0		0		0		0		0		0		CaCe	0	Ixc	W		
513	Leguminosae		SRL-1370	Guaje de gamito			0		0		0		0		0		0		CaCe	0	Ixc	W		
514	Leguminosae		SRL-1371	Espino			0		0		0		0		0		0		CaCe	0	Ixc	W		
515	Leguminosae		SRL-1498				0		0		0		0		0		0		CaMy	0	Ixc	W		
516	Leguminosae		SRL-1217		2		0	-0.6097	0		0	-0.8011	0		0		0		SB	0	Ixc	W	F, G	*in situ*
517	Lentibulariaceae	*Pinguicula moranensis* Kunth	RLF-148, SRL-436, SRL-1553, SRL, 1496	Siempreviva			0		0		0		0		0		0		BG, Me, Palm	0.001489	Ixc	W		
518	Linaceae	*Linum scabrellum* Planch.	SRL-1462				0		0		0		0		0		0		Me	0	Ixc	W		
519	Linaceae	*Linum* sp.	RLF-175		2		0	-0.2201	0		0	-0.5074	0		0		0		Palm, TS	0	Ixc	W	F, G, T, Ur	*in situ*
520	Loasaceae	*Mentzelia hispida* Willd.	RLF-54, RLF-94, SRL-428	Pegajosa	1		0		0		0	-0.755	0		0		0		Bal, BEA, BG	0	Ixc	W	G, T, Ur	*in situ*
521	Loranthaceae	*Psittacanthus calyculatus* (DC.) G.Don	SRL-1502	Injerto	1		0	-0.7648	0		0		0		0		0		CaMy, Sol, TS	0	Ixc	W	Ur	*in situ*
522	Lythraceae	*Cuphea* sp.	RLF-100, RLF-143, RLF-172, SRL-20, SRL-350, SRL-1178		3		0	0.0939	0		0	-0.3167	0		0	-0.8814	0		Me, Sol, Palm, TS	0.000112	Ixc	W	F, G, T, Ur	*in situ*
523	Lythraceae	*Cuphea* sp.	SRL-25		1		0		0		0		0		0	-1.4276	0		BN, Palm, Sol	0.010633	Ixc	W	G	*in situ*
524	Lythraceae	*Cuphea* sp.	SRL-105, SRL-296		1		0		0		0		0		0	-1.563	0		BEA, BN, Paz	0.003757	Ixc	W	G	*in situ*
670	Lythraceae	*Punica granatum* L.	ERL-38, ERL-39, ERL-70, ERL-71, ERL-104, ERL-206, SRL-43	Granada	5	Ornamental = 71, edible = 10	0		0.0147		0		0.0129		0		0		Sol	0.000307	EAAA	D	E, P, Prp, T, Ti	*ex situ*
525	Malpighiaceae	*Bunchosia* sp.	SRL-451	Huevo de gato	2		0		0		0		0	-1.0654	0		0		Sol	0	Ixc	W	G, T	*in situ*
526	Malpighiaceae	*Bunchosia* sp.	SRL-1351				0		0		0		0		0		0		CaCe	0	Ixc	W		
527	Malpighiaceae	*Echinopterys eglandulosa* (A.Juss.) Small	SRL-1384				0		0		0		0		0		0		SB	0	Ixc	W		
528	Malpighiaceae	*Galphimia multicaulis* A.Juss.	RLF-65, RLF-293, SRL-1177	Flor de chivo	2		0	-0.5325	0		0		0		0	-1.3063	0		BEA, BEC, Iz, Me, Palm	0.002876	Ixc	W	F, G	*in situ*
529	Malpighiaceae	*Gaudichaudia galeottiana* (Nied.) Chodat	RLF-241		1		0		0		0	-1.0487	0		0		0		Iz	0	Ixc	W	G	*in situ*
530	Malpighiaceae	*Heteropterys brachiata* (L.) DC.	SRL-1342				0		0		0		0		0		0		CaCe	0	Ixc	W		
531	Malpighiaceae	*Malpighia galeottiana* A.Juss.	SRL-362, SRL-471, SRL-1272	Nanche	4	Edible = 10	0.0018	0.3567	0		0		0	-0.6124	0		0		Me, Palm, TS	0.001293	Ixc	W	F, G, T	*in situ*
532	Malvaceae	*Alcea rosea* L.	ERL-140, ERL-201, ERL-227, SRL-62, SRL-187	Flor de San José	2	Ornamental = 29	0		0.0042		0		0		0		0		Sol	0.000128	EAAA	D	P, Prp	*ex situ*
533	Malvaceae	*Anoda cristata* (L.) Schltdl.	RLF-67, RLF-277, SRL-6, SRL-446, SRL-1125	Quelite de malva, violeta	4	Fodder = 40, ornamental = 6, edible = 5	0	0.5126	0	-0.4235	0	-0.1293	0.0117	-0.4905	0		0		Bal, BEA, Pal, Sol, TS	0.000026	Ixc	R-W	F, G, T, Ur	*in situ*
534	Malvaceae	*Gossypium hirsutum* L.	Photo record	Algodón	1		0		0		0		0		0		0		Sol	0	TCV	D	P, Prp	*ex situ*
755	Malvaceae	*Hermannia inflata* Link & Otto	SRL-1301				0		0		0		0		0		0		Me	0	Ixc	W		
535	Malvaceae	*Hibiscus rosa-sinensis* L.	ERL-207	Tulipán	2	Ornamental = 6	0		0.0042		0		0		0		0		Sol	0.000026	EAAA	D	P, Ti	*ex situ*
536	Malvaceae	*Hibiscus* sp.	SRL-1474		1		0		0		0		0		0		0		Sol	0	EAAA	W	P, Ti	*ex situ*
537	Malvaceae	*Malva parviflora* L.	ERL-30, ERL-90, SRL-205, SRL-1124, SRL-1143	Malva	3	Fodder = 5, ornamental = 47, medicinal = 50	0.0194		0		0.0324		0		0		0		Bal, Sol, TS	0.000205	Nat-EAAA	R-W	F, G, T, Ur	*ex situ*
538	Malvaceae	*Malva sylvestris* L.	ERL-111, ERL-210	Malva rosa, malva de castilla	2	Ornamental = 12	0		0		0		0		0		0		Sol	0.000051	EAAA	W	P, Prp, Ti	*ex situ*
539	Malvaceae	*Sida* sp.	SRL-21		1		0	-1.0765	0		0		0		0		0		Bal, Sol	0	Ixc	W	F	*in situ*
540	Martyniaceae	*Proboscidea louisianica* (Mill.) Thell.	SRL-1318	Cuerno de toro	1		0		0		0		0.0038	-1.148	0		0		Bal, Palm, Sol, TS	0.000026	Ixc	W	G, T	*in situ*
541	Meliaceae	*Cedrela* sp.	ERL-60		1	Ornamental = 18	0		0		0		0		0		0		Sol	0.000077	Mex	W	P, Ti	*ex situ*
542	Meliaceae	*Melia azedarach* L.	ERL-2, SRL-53	Clavo, paraíso	2	Ornamental = 6	0		0		0		0		0		0		Sol	0.000026	EAAA	W	P, Ti	*ex situ*
543	Meteoriaceae	*Meteorium deppei* (Hornsch. ex Müll. Hal.) Mitt.	SRL-1432	Musgo	2	Ceremonial = 2	0		0.0165	0.6329	0		0		0	0.501	0		BEA, BM, Sol	0	Ixc	W	G, P, Ti	*ex situ, in situ*
544	Moraceae	*Ficus benjamina* L.	SRL-1170	Laurel de la India	2		0		0.0294		0		0		0		0		Sol	0	EAAA	W	P, Ti	*ex situ*
545	Moraceae	*Ficus carica* L.	ERL-125	Higo	1	Edible = 15	0		0		0		0.0219		0		0		Sol	0.000179	EAAA	D	P, Prp, Ti	*ex situ*
546	Moraceae	*Ficus crocata* (Miq.) Mart. ex Miq.	SRL-76, SRL-1171	Amate	3	Ornamental = 6	0		0.0049	-0.6478	0		0	-0.8491	0		0		Sol	0.000026	Ixc	W	G, T	*in situ*
547	Moraceae	*Ficus microcarpa* L. f.	ERL-115	Laurel	2	Ornamental = 18	0		0.0294		0		0		0		0		Sol	0.000077	EAAA	W	P, Ti	*ex situ*
548	Moraceae	*Ficus pertusa* L.f.	SRL-433				0		0		0		0		0		0		BG	0.001066	Ixc	W		
549	Moraceae	*Morus celtidifolia* Kunth	ERL-55, ERL-78, ERL-55, ERL-78, ERL-124, ERL-128, ERL-129, ERL-214, ERL-220, ERL-221, RLF-92, SRL-55, SRL-1517	Moral, morera	8	Ornamental = 88, firewood = 100	0.0116	1.9551	0.0118	3.3295	0		0.0096	0.3611	0		0.0161	0.4875	AA, Sol	0.000384	Ixc	W	P, T	*in situ*
550	Musaceae	*Musa × paradisiaca* L.	Photo record	Plátano	2	Ornamental = 12, edible = 100	0		0.0074		0		0.0132		0		0		Sol	0.000051	EAAA	D	P, Ti	*ex situ*
551	Myrtaceae	*Eucalyptus camaldulensis* Dehnh.	SRL-203	Eucalipto	2		0		0		0.0019		0		0		0		Pal	0	EAAA	W	Ti	*ex situ*
552	Myrtaceae	*Psidium guajava* L.	SRL-1528	Guayaba	1		0		0		0		0.0263		0		0		BG	0	Mex	D	T	*ex situ*
556	Nyctaginaceae	*Boerhavia anisophylla* Torr.	SRL-162, SRL-193, SRL-370, SRL-1184, SRL-1303		1		0	-0.5246	0		0		0		0		0		Bal, BEA, Me, Pal, Palm, Sol, TS	0.000241	Ixc	R-W, W	F, G, T, Ur	*in situ*
557	Nyctaginaceae	*Bougainvillea spectabilis* Willd.	SRL-33, SRL-191	Bugambilia	3	Ornamental = 18	0		0.0529		0		0		0.0025		0		Sol	0.000077	AC	D	P, Prp, Ti	*ex situ*
558	Nyctaginaceae	*Mirabilis jalapa* L.	ERL-29, ERL-99, SRL-11, SRL-421, SRL-1145	Hierba cuchi, maravilla	3	Fodder = 50, ornamental = 29	0	0.2319	0	-0.0608	0	-0.3165	0		0		0		Bal, BG, Sol	0.000128	Ixc	R-W	F, G, T, Ur	*in situ*
559	Oleaceae	*Forestiera rotundifolia* (Brandegee) Standl.	RLF-306, SRL-1259	Tlasisle	3		0.0025	0.0567	0		0		0		0		0	-1.218	BG, Me, TS	0.001730	Ixc	W	F, G, T	*in situ*
560	Oleaceae	*Fraxinus purpusii* Brandegee	SRL-341, SRL-1463, SRL-1512	Zapotillo, fresno	3	Firewood = 100	0.0076	-0.307	0		0		0		0		0.0137	-1.0373	BEM, Me	0	Ixc	W	F, G	*in situ*
561	Oleaceae	*Fraxinus uhdei* (Wenz.) Lingelsh.	SRL-1409	Fresno	1		0		0		0		0		0		0		BEA	0	Ixc	W	G	*in situ*
562	Oleaceae	*Ligustrum japonicum* Thunb.	ERL-105, ERL-238, SRL-59, SRL-453	Trueno	4	Ornamental = 18, ceremonial = 22	0		0.0235		0		0		0		0		Sol	0.000077	EAAA	W	P, Ti	*ex situ*
563	Onagraceae	*Fuchsia* sp.	SRL-386, SRL-393				0		0		0		0		0		0		BEA	0	Ixc	W		
564	Onagraceae	*Gaura coccinea* Nutt. ex Pursh	SRl-17, SRL-411	Gradiolita	2		0	-0.2194	0		0	-0.507	0		0		0		Bal, Sol	0.000026	Ixc	W	F, G, T, Ur	*in situ*
565	Onagraceae	*Lopezia racemosa* Cav.	ERL-114, SRL-1, SRL-94, SRL-1323		1		0		0		0		0		0		0		Bal, Sol, TS	0.006657	Ixc	R-W	G, T, Ur	*in situ*
566	Onagraceae	*Oenothera pubescens* Willd. ex Spreng.	RLF-76, RLF-113, SRL-22, SRL-40, SRL-150, SRL-213	Campanita grande	2	Ornamental = 12	0		0	-0.8404	0	-0.5653	0		0		0		Bal, BEA, Me, Sol	0.000051	Ixc	R-W, W	G, T, Ur	*in situ*
567	Onagraceae	*Oenothera rosea* L´Her. ex Aiton	SRL-1127, SRL-1322	Sanguinaria	2	Ornamental = 12	0		0	-0.8404	0	-0.5653	0		0		0		Bal, Sol	0.000051	Ixc	R-W, W	G, T, Ur	*in situ*
568	Orchidaceae	*Barkeria lindleyana* subsp. *vanneriana* (Rchb.f.) Thien	SRL-1509	Monjita de peña	2	Ceremonial = 8	0		0	0.1802	0		0		0	0.5508	0		CaMy	0	Ixc	W	G, P, Ti	*ex situ, in situ*
569	Orchidaceae	*Corallorhiza* sp.	RLF-207	Flor de jarrita			0		0		0		0		0		0		Iz	0	Ixc	W		
571	Orchidaceae	*Cyrtopodium macrobulbon* (Lex.) G.A.Romero & Carnevali	Photo record	Jarrito	2		0	-0.1422	0	-1.0573	0		0		0		0		Me	0	Ixc	W	F, P	*in situ*
572	Orchidaceae	*Dichromanthus cinnabarinus* (Lex.) Garay	RLF-223, RLF-289, SRL-1155, SRL-1172	Cola de león	3		0		0	-1.1298	0	-0.6711	0		0	-1.1913	0		BEA, Iz, Palm	0	Ixc	W	G	*in situ*
574	Orchidaceae	*Encyclia hanburyi* (Lindl.) Schltr.	SRL-1519	Monjita morada de campo	2		0		0.0074	0.3814	0		0		0	0.4864	0		Me, Sol	0	Ixc	W	G, P, Ti	*ex situ, in situ*
575	Orchidaceae	*Epidendrum lignosum* Lex.	RJS-9, RLF-50, SRL-139	Flor de cañada	1		0		0	-0.0825	0		0		0		0		BEA, Pal	0	Ixc	W	G, P, Ti	*ex situ, in situ*
576	Orchidaceae	*Epidendrum longipetalum* A.Rich. & Galeotti	RJS-6	Monjita moradita de varas	1		0		0	-1.2721	0		0		0		0		BEA	0	Ixc	W	Ti	*in situ*
577	Orchidaceae	*Epidendrum radioferens* (Ames, F.T.Hubb. & C.Schweinf.) Hágsater	RJS-3	Monjita colorada	2	Ornamental = 12, ceremonial = 85	0		0.0139	0.8741	0		0		0	1.1964	0		BEA, BEM, Pal, Sol	0.000051	Ixc	W	G, P, Ti	*ex situ, in situ*
584	Orchidaceae	*Euchile karwinskii* (Mart.) Christenson	RJS-1	Monjita amarilla	3	Ornamental = 47, ceremonial = 99	0		0.045	3.5005	0.0017	2.6178	0		0.0333	2.2487	0		BEA, Pal, Sol	0.000205	Ixc	W	G, P, Prp, Ti	*ex situ, in situ*
578	Orchidaceae	*Govenia lagenophora* Lindl.	SRL-1270	Jarrito	3		0	0.1688	0	-0.7946	0		0		0		0		Me	0	Ixc	W	F, P	*in situ*
573	Orchidaceae	*Homalopetalum kienastii* (Rchb.f.) Withner	SRL-1249		1		0		0	-0.1783	0		0		0		0		BEA	0	Ixc	W	P, Ti	*ex situ, in situ*
579	Orchidaceae	*Laelia albida* Bateman ex Lindl.	ERL-126	Monjita blanca	2	Ornamental = 59, ceremonial = 77	0		0.0433	3.0505	0		0		0.0524	1.2962	0		Pal, Sol, TS	0.000281	Ixc	W	P, Prp, Ti	*ex situ*
580	Orchidaceae	*Laelia anceps* Lindl.	SRL-1541	Monjita morada	2	Ornamental = 35, ceremonial = 77	0		0.0497	2.6014	0		0		0.0439	1.2722	0		AA, Pal, Sol	0.000153	Ixc	W	P, Prp, Ti	*ex situ*
581	Orchidaceae	*Malaxis unifolia* Michx.	SRL-1196				0		0		0		0		0		0		BEA	0	Ixc	W		
582	Orchidaceae	*Oncidium brachyandrum* Lindl.	RJS-5	Monjita pinta amarilla	1		0		0	-1.2721	0		0		0		0		BEA	0	Ixc	W	Ti	*in situ*
570	Orchidaceae	*Ponthieva mexicana* (A.Rich. & Galeotti) Salazar	RLF-256, RLF-267				0		0		0		0		0		0		BEA, Iz	0	Ixc	W		
583	Orchidaceae	*Prosthechea concolor* (Lex.) W.E.Higgins	RJS-2, SRL-1189	Monjita pintita chiquita	1		0		0	-0.1783	0		0		0		0		BEA, Pal	0	Ixc	W	P, Ti	*ex situ, in situ*
585	Orchidaceae	*Prosthechea vitellina* (Lindl.) W.E.Higgins	Photo record	Monjita	1		0		0	-1.2721	0		0		0		0		BEA	0	Ixc	W	Ti	*in situ*
586	Orchidaceae	*Rhynchostele maculata* (Lex.) Soto Arenas & Salazar	ERL-173, SRL-1476	Monjita pinta	2	Ornamental = 6, ceremonial = 92	0		0.0174	0.8134	0		0		0.0046	1.2724	0		BEA, BEM, Pal, Sol	0.000026	Ixc	W	G, P, Ti	*ex situ, in situ*
587	Orchidaceae	*Spiranthes* sp.	RLF-208	Monjita de peña	1		0		0		0		0		0	-1.6375	0		Iz	0	Ixc	W	G	*in situ*
588	Orchidaceae		Photo record	Monjita	1		0		0	-0.1783	0		0		0		0		BEA, Me, Pal	0	Ixc	W	P, Ti	*ex situ, in situ*
589	Orchidaceae		Photo record	Monjita	1		0		0	-0.1783	0		0		0		0		BEA, Me, Pal	0	Ixc	W	P, Ti	*ex situ, in situ*
590	Orchidaceae		Photo record	Monjita de camotito largo	1	Ornamental = 6	0		0	-0.0219	0		0		0		0		NE, Sol	0.000026	Ixc	W	P, Ti	*ex situ, in situ*
719	Orobanchaceae	*Buchnera pusilla* Kunth	RLF-235		1		0		0	-2.1063	0		0		0		0		Iz	0	Ixc	W		
720	Orobanchaceae	*Castilleja tenuifolia* M.Martens & Galeotti	SRL-117, SRL-223, SRL-329, SRL-1438, SRL-1485	Romero cimarrón	3		0	-0.1987	0		0	-0.5504	0		0	-1.0665	0		BEA, BN, Me, Palm	0.003728	Ixc	R-W, W	F, G	*in situ*
591	Orobanchaceae	*Conopholis alpina* Liebm.	SRL-218, SRL-1481	Flor de elote	2		0	-0.7655	0		0	-0.9186	0		0		0		BEA, Pal	0	Ixc	W	F	*in situ*
722	Orobanchaceae	*Lamourouxia dasyantha* (Cham. & Schltdl.) W.R.Ernst	SRL-1379, SRL-1429	Lisión	2	Ceremonial = 17	0		0.0059	-1.2315	0		0		0.0389	-1.1735	0		BEA, BEC, BEM, Me	0	Ixc	R-W, W	G	*in situ*
723	Orobanchaceae	*Lamourouxia viscosa* Kunth	RLF-209, SRL-372, SRL-1292	Moco de pavo, flor de miel	1		0		0		0		0		0	-1.4246	0		Iz, Pal, Palm	0.000396	Ixc	R-W, W	G, T	*in situ*
594	Oxalidaceae	*Oxalis corniculata* L.	SRL-1534	Coyule			0		0		0		0		0		0		Bal, Sol	0	Ixc	R-W	T	*in situ*
592	Oxalidaceae	*Oxalis aff. latifolia* Kunth	ERL-75, RLF-142, SRL-148	Coyule	2	Edible = 45	0	1.1914	0		0		0	-0.0837	0		0		Iz, Me, Sol, TS	0.038091	Ixc	W	F, P, T	*in situ*
593	Oxalidaceae	*Oxalis aff. nelsonii* (Small) R.Knuth	SRL-1273	Coyule	2	Edible = 45	0	2.8029	0		0		0.0066	1.1688	0		0		Iz, Sol	0.000026	Ixc	W	F, G, P, Prp	*ex situ, in situ*
595	Oxalidaceae	*Oxalis* sp.	RLF-139	Coyule delgado	2		0	0.095	0		0		0	-0.7869	0		0		BEA, BEC, BN, Me	0.026267	Ixc	W	F, G	*in situ*
596	Papaveracea	*Argemone mexicana* L.	ERL-244, RLF-180, SRL-455	Chicalote	3		0		0		0	-0.3555	0		0	-0.909	0		Bal, Pal, Sol, TS	0.001314	Ixc	R-W	G, T, Ur	*in situ*
597	Passifloraceae	*Passiflora bryonioides* Kunth	SRL-1148	Granadilla	1		0		0		0		0	-0.7604	0		0		Sol	0	Ixc	W	G, P, T	*in situ*
598	Passifloraceae	*Passiflora suberosa* L.	SRL-444, SRL-1164, SRL-1165		1		0		0		0	-0.8137	0		0		0		Sol	0	Ixc	W	G, T	*in situ*
761	Passifloraceae	*Turnera diffusa* Willd. ex Schult.	SRL-1220, SRL-1356, SRL-1467	Tamorreal	3	Medicinal = 5	0		0		0.037	2.85	0	1.1156	0		0		CaCe, SB, Sol	0	Ixc	W	G, P, Ti	*ex situ, in situ*
721	Phrymaceae	*Berendtiella levigata* (B.L.Rob. & Greenm.) Thieret	RLF-229	Hierba de pajarito	1		0	-1.0765	0		0		0		0		0		Iz	0	Ixc	W	F	*in situ*
599	Phytolaccaceae	*Phytolacca icosandra* L.	RLF-236		1		0		0		0	-1.0487	0		0		0		Iz	0	Ixc	R-W	G	*in situ*
600	Pinaceae	*Pinus* sp.	SRL-185	Pino, ocote	3	Ornamental = 47	0		0.0331		0		0		0		0		Palm, Sol	0.000205	Mex	W	P, Ti	*ex situ*
601	Piperaceae	*Peperomia quadrifolia* (L.) Kunth	ERL-146, SRL-1404, 1430	Verdolaga	1	Edible = 95	0		0		0		0.0697	1.3422	0		0		BEM	0	Ixc	W	G, P, Ti	*ex situ, in situ*
602	Piperaceae	*Peperomia* sp.	RJS-4		1		0	-1.0765	0		0		0		0		0		BEA	0	Ixc	W	F	*in situ*
603	Piperaceae	*Piper auritum* Kunth	ERL-59, SRL-67, SRL-418	Hierba santa	2		0		0		0		0.0103		0		0		Pal, Sol	0.000102	Mex	W	P, Prp, Ti	*ex situ*
717	Plantaginaceae	*Antirrhinum majus* L.	Photo record	Perrito	2	Ornamental = 12	0		0.0147		0		0		0		0		Sol	0.000051	EAAA	D	P, Prp	*ex situ*
718	Plantaginaceae	*Bacopa monnieri* (L.) Wettst.	SRL-301, SRL-1132	Verdolaga de agua	3	Edible = 5	0	-0.2864	0		0	-0.6047	0.0096	-0.9247	0		0		Paz, VR	0.000458	Ixc	W	F, G	*in situ*
724	Plantaginaceae	*Maurandya barclaiana* Lindl.	ERL-171		1	Ornamental = 18	0		0	-1.0904	0		0		0		0		Sol	0.000077	Ixc	R-W, W	T	*in situ*
725	Plantaginaceae	*Penstemon barbatus* (Cav.) Roth	RLF-23, RLF-49, SRL-133, SRL-464, SRL-1314	Bandera	2		0		0		0	-0.8535	0		0	-1.4068	0		BEA, Palm	0.000385	Ixc	R-W, W	G	*in situ*
726	Plantaginaceae	*Penstemon roseus* (Cerv. ex Sweet) G.Don	SRL-124, SRL-1405	Bandera	1		0	-1.0765	0		0		0		0		0		BEA	0	Ixc	R-W, W	F	*in situ*
604	Plantaginaceae	*Plantago major* L.	SRL-419				0		0		0		0		0		0		BG, VR	0	Nat-EAAA	W		
727	Plantaginaceae	*Russelia obtusata* S.F.Blake	RLF-263, SRL-234, SRL-342, SRL-424, SRL-1494	Bandera	1		0		0		0	-0.9867	0		0		0		BEA, BG, BN, Me	0.003738	Ixc	R-W, W	G	*in situ*
728	Plantaginaceae	*Veronica persica* Poir.	SRL-177, SRL-1327		1		0		0		0		0		0		0		Sol	0	EAAA	W	P, Prp	*ex situ*
729	Plantaginaceae		SRL-1198	Bandera	1		0		0		0	-1.0487	0		0		0		BEA	0	Ixc	W	G	*in situ*
605	Plumbaginaceae	*Plumbago pulchella* Boiss.	SRL-189, SRL-1278				0		0		0		0		0		0		BG, Pal	0	Ixc	R-W	T, Ur	*in situ*
606	Poaceae	*Aegopogon cenchroides* Humb. & Bonpl. ex Willd.	SRL-83	Pasto	2	Fodder = 20	0.1738	-0.3545	0.0074	-0.904	0		0		0		0		Bal	0	Ixc	W	F, G, T, Ur	*in situ*
607	Poaceae	*Aristida adscensionis* L.	RLF-239, SRL-354	Pasto	3		0.1738		0.0074		0		0		0		0		BN, Iz, Me	0.001851	Nat-EAAA	W	F, G	*ex situ*
608	Poaceae	*Aristida jorullensis* Kunth	SRL-142	Pasto de semilla	2		0.1738	-0.955	0.0074	-1.2392	0		0		0		0		BEA	0	Ixc	W	F	*in situ*
609	Poaceae	*Aristida schiedeana* Trin. & Rupr.	SRL-309	Pasto	2		0.1738	1.0277	0.0074	0.5759	0		0		0		0		BEA, BEC, BN, Me, Palm, Paz, TS	0.059386	Ixc	W	F, T, Ur	*in situ*
610	Poaceae	*Arundo donax* L.	ERL-147, SRL-429	Carrizo	4		0		0		0		0		0		0		BG, Pal, Sol, VR	0.001636	Nat-EAAA	W	F, P, Prp	*ex situ*
611	Poaceae	*Avena fatua* L.	SRL-1546	Avena	1	Fodder = 10	0.1041		0		0		0		0		0		Bal, Sol, TS	0	Nat-EAAA	D	F, P, Prp, T, Ur	*ex situ*
612	Poaceae	*Bouteloua curtipendula* (Michx.) Torr.	RLF-98, RLF-237, RLF-296	Pasto	2	Fodder = 20	0.1738	-0.3545	0.0074	-0.904	0		0		0		0		Bal, Iz, Sol	0	Ixc	W	F, G, T, Ur	*in situ*
614	Poaceae	*Chloris rufescens* Lag.	RLF-99	Pastón, cebadia, gabilla	2	Fodder = 20	0.1738	-0.3545	0.0074	-0.904	0		0		0		0		Bal	0	Ixc	W	F, G, T, Ur	*in situ*
615	Poaceae	*Chloris submutica* Kunth	SRL-38	Pastón	2	Fodder = 20	0.1738	-0.3545	0.0074	-0.904	0		0		0		0		Bal	0	Ixc	W	F, G, T, Ur	*in situ*
613	Poaceae	*Chondrosum simplex* (Lag.) Kunth	SRL-305	Pasto	2		0.1738	-0.8225	0.0074	-1.1081	0		0		0		0		Paz	0.004938	Ixc	W	F	*in situ*
616	Poaceae	*Cymbopogon citratus* (DC.) Stapf	Photo record	Té limón, té de pasto	1		0		0		0		0		0		0		Sol	0.000051	EAAA	W	P, Prp	*ex situ*
617	Poaceae	*Dactyloctenium aegyptium* (L.) Willd.	SRL-86	Pasto de semilla	2		0.1738		0.0074		0		0		0		0		Bal	0	Nat-EAAA	R-W	F	*ex situ*
618	Poaceae	*Digitaria bicornis* (Lam.) Roem. & Schult.	SRL-312	Pasto	2		0.1738		0.0074		0		0		0		0		Paz	0.000709	Nat-EAAA	R-W	F	*ex situ*
620	Poaceae	*Eragrostis intermedia* Hitchc.	RLF-164, SRL-306	Pasto	2	Fodder = 20	0.1738	-0.2115	0.0074	-0.7625	0		0		0		0		Paz, TS	0.005333	Ixc	W	F, G, T, Ur	*in situ*
621	Poaceae	*Eragrostis mexicana* (Hornem.) Link	SRL-84	Pasto	2	Fodder = 20	0.1738	-0.3545	0.0074	-0.904	0		0		0		0		Bal	0	Ixc	W	F, G, T, Ur	*in situ*
619	Poaceae	*Eragrostis aff. pectinacea* (Michx.) Nees	SRL-85	Pasto legítimo	2	Fodder = 20	0.1738	-0.3545	0.0074	-0.904	0		0		0		0		Bal	0	Ixc	W	F, G, T, Ur	*in situ*
622	Poaceae	*Erioneuron avenaceum* (Humb., Bonpl. & Kunth) Tateoka	RLF-292	Pasto	2		0.1738	-0.955	0.0074	-1.2392	0		0		0		0		Iz	0	Ixc	W	F	*in situ*
623	Poaceae	*Heteropogon contortus* (L.) P.Beauv. ex Roem. & Schult.	RLF-202	Pasto	2		0.1738	-0.955	0.0074	-1.2392	0		0		0		0		Iz	0	Ixc	W	F	*in situ*
624	Poaceae	*Hilaria cenchroides* Kunth	SRL-281, SRL-308	Pasto	2		0.1738	-0.8824	0.0074	-1.1673	0		0		0		0		BN, Palm, Paz	0.002708	Ixc	W	F	*in situ*
625	Poaceae	*Hordeum vulgare* L.	Photo record	Cebada	1	Fodder = 10	0.0794		0		0		0		0		0		Sol, TS	0.000026	EAAA	D	P, Prp, T	*ex situ*
626	Poaceae	*Lasiacis* sp.	SRL-1506	Otate	1		0		0.0074	-1.9051	0		0		0		0		Me	0	Ixc	W		
627	Poaceae	*Lycurus phleoides* Kunth	SRL-307	Pasto	2		0.1738	-0.8745	0.0074	-1.1595	0		0		0		0		Paz	0.003002	Ixc	W	F	*in situ*
628	Poaceae	*Muhlenbergia gigantea* (E.Fourn.) Hitchc.	RLF-305	Pastón	2		0		0.0074	-1.1597	0		0		0		0		Iz	0.001189	Ixc	W	G	*in situ*
629	Poaceae	*Muhlenbergia robusta* (E.Fourn.) Hitchc.	RLF-66, SRL-169	Pastón	2		0		0.0074	-1.0966	0		0		0		0		BEA, BG	0.003568	Ixc	W	G	*in situ*
630	Poaceae	*Nassella tenuissima* (Trin.) Barkworth	RLF-258	Pasto	2		0.1738	-0.955	0.0074	-1.2392	0		0		0		0		Iz	0	Ixc	W	F	*in situ*
631	Poaceae	*Oryza sativa* L.	Photo record	Arroz	1		0		0		0		0		0		0		Sol	0.000026	EAAA	D	E, P	*ex situ*
632	Poaceae	*Otatea acuminata* (Munro) C.E.Calderón & Soderstr.	RLF-250	Otate	2		0		0	-1.3925	0		0		0		0		Iz	0	Ixc	W	G	*in situ*
638	Poaceae	*Panicum maximum* Jacq.	RLF-147	Pasto cenizo, pastón	2		0.1738		0.0074		0		0		0		0		Paz	0	Nat-EAAA	R-W	F	*ex situ*
633	Poaceae	*Phalaris canariensis* L.	ERL-231	Alpiste	1		0		0		0		0		0		0		Sol	0.000026	EAAA	D	P, Prp	*ex situ*
634	Poaceae	*Piptochaetium fimbriatum* (Humb., Bonpl. & Kunth) Hitchc.	RLF-137, SRL-260, SRL-413	Pasto	3		0.1738	-0.3887	0.0074	-0.7823	0	-0.5508	0		0		0		BEA, BG, Me, Paz	0.003708	Ixc	W	F, G	*in situ*
635	Poaceae	*Setaria grisebachii* E.Fourn.	RLF-231,RL-358	Pasto de semilla	3		0.1738	-0.4232	0.0074	-0.8164	0	-0.5721	0		0		0		Iz, Palm, Paz	0.002422	Ixc	W	F, G	*in situ*
636	Poaceae	*Sporobolus indicus* (L.) R.Br.	RLF-132	Pastón	3		0.1738	-0.4882	0.0074	-0.8807	0		0		0		0		Me	0	Ixc	W	F, G	*in situ*
637	Poaceae	*Triticum aestivum* L.	SRL-172	Trigo	2	Edible = 95	0.0573		0		0		0.0344		0		0		Sol, TS	0	EAAA	D	P, Prp	*ex situ*
639	Poaceae	*Zea mays* L.	SRL-174	Maíz	3	Fodder = 80, edible = 100, ceremonial = 1	0.3047		0		0		0.0376		0		0		Sol, TS	0.000230	Mex	D	F, P, Prp, T	*ex situ*
640	Poaceae		RLF-157	Pasto	3		0.1738	-0.4882	0.0074	-0.8807	0		0		0		0		Paz	0	Ixc	W	F, G	*in situ*
641	Poaceae		SRL-311	Pasto de semilla	3		0.1738	-0.3818	0.0074	-0.7755	0	-0.5465	0		0		0		Paz	0.003967	Ixc	W	F, G	*in situ*
642	Poaceae		SRL-258	Pasto	2		0.1738	-0.7199	0.0074	-1.0066	0		0		0		0		BEA, BEC	0.008764	Ixc	W	F	*in situ*
643	Poaceae		RLF-291	Pasto	2		0.1738	-0.4149	0.0074	-0.705	0		0		0		0		Iz	0.020134	Ixc	W	F	*in situ*
644	Poaceae		RLF-316	Pasto	2		0.1738	-0.955	0.0074	-1.2392	0		0		0		0		Iz	0	Ixc	W	F	*in situ*
645	Poaceae		RLF-331	Pasto	2		0.1738	-0.955	0.0074	-1.2392	0		0		0		0		Iz	0	Ixc	W	F	*in situ*
646	Poaceae		RLF-332	Pasto	2		0.1738	-0.955	0.0074	-1.2392	0		0		0		0		iz	0	Ixc	W	F	*in situ*
647	Poaceae		RLF-333	Pasto	2		0.1738	-0.955	0.0074	-1.2392	0		0		0		0		iz	0	Ixc	W	F	*in situ*
648	Poaceae		SRL-394	Pasto	2		0.1738	-0.955	0.0074	-1.2392	0		0		0		0		BEA	0	Ixc	W	F	*in situ*
649	Poaceae		RLF-317	Pasto	2	Fodder = 20	0.1738	-0.3545	0.0074	-0.904	0		0		0		0		Bal, Sol	0	Ixc	W	F, G, T, Ur	*in situ*
650	Polemoniaceae	*Loeselia caerulea* (Cav.) G.Don	RLF-265, SRL-96, SRL-353, SRL-1267, SRL-1282, SRL-1364, SRL-1401, SRL-1458		2		0	-0.2933	0		0	-0.6054	0		0		0		BEA, BN, CaCe, Me, Pal, Palm	0.011792	Ixc	W	F, G	*in situ*
651	Polygalaceae	*Polygala compacta* Rose	SRL-255				0		0		0		0		0		0		BEA, BEC, Iz	0.008358	Ixc	W		
652	Polygalaceae	*Polygala scoparia* Kunth	RLF-224, RLF-287		2		0	-0.4	0		0	-0.8599	0		0		0		BN, Iz	0.007838	Ixc	W	F, G	*in situ*
653	Polygonaceae	*Rumex crispus* L.	SRL-1533				0		0		0		0		0		0		Bal, Sol	0	Nat-EAAA	W	T	*ex situ*
654	Polypodiaceae	*Pleopeltis conzattii* (Weath.) R.M.Tryon & A.F.Tryon	RLF-46, SRL-135, SRL-1237		1		0	-1.0765	0		0		0		0		0		BEA, BEM	0	Ixc	W	F	*in situ*
655	Polypodiaceae	*Pleopeltis polylepis* (Roemer ex Kunze) T.Moore	SRL-1434				0		0		0		0		0		0		BEM	0	Ixc	W		
656	Polypodiaceae	*Polypodium martensii* Mett.	RLF-47, SRL-137, SRL-1433	Cilandrillo	2		0	-0.7655	0	-1.4404	0		0		0		0		BEA, BEM	0	Ixc	W	F	*in situ*
658	Polypodiaceae	*Polypodium thyssanolepis* A.Braun ex Klotzsch	RLF-294	Cilandrillo			0		0		0		0		0		0		Iz	0.001316	Ixc	W		
657	Polypodiaceae	*Polypodium* sp.	SRL-352	Cilandrillo			0		0		0		0		0		0		Me	0.000872	Ixc	W		
660	Portulacaceae		SRL-415		1		0		0		0		0		0		0		BG	0.003064	Nat-Uk	W	F	*ex situ*
661	Primulaceae	*Anagallis arvensis* L.	ERL-108, ERL-228, RLF-200, SRL-87, SRL-100, SRL-1133	Jabonera, hierba de pollo	3		0		0		0.0065		0.0066		0		0		Bal, BN, Iz, Palm, Sol, TS	0.002474	Nat-EAAA	R-W	G, T	*ex situ*
759	Primulaceae	*Bonellia macrocarpa* (Cav.) B.Ståhl & Källersjö	SRL-1330				0		0		0		0		0		0		CaCe	0	Ixc	W		
662	Proteaceae	*Grevillea robusta* A.Cunn. ex R.Br.	ERL-6		2	Ornamental = 12	0		0.0042		0		0		0		0		Sol	0.000051	EAAA	W	P, Ti	*ex situ*
663	Pteridaceae	*Adiantum capillus-veneris* L.	SRL-1518				0		0		0		0		0		0		Me	0	Ixc	W		
664	Pteridaceae	*Adiantum poiretii* Wikstr.	SRL-202,SRL-427		1		0		0		0	-0.9676	0		0		0		BG, VR	0.004886	Ixc	W	G	*in situ*
665	Pteridaceae	*Astrolepis crassifolia* (Houlston & T.Moore) D.M.Benham & Windham	RLF-34, SRL-389				0		0		0		0		0		0		BEA	0	Ixc	W		
666	Pteridaceae	*Cheiloplecton rigidum* (Sw.) Fée	RLF-112, RLF-213, RLF-254, SRL-1457	Cilandrillo			0		0		0		0		0		0		Iz, Me	0	Ixc	W		
667	Pteridaceae	*Notholaena* sp.	SRL-230				0		0		0		0		0		0		VR	0	Ixc	W		
668	Pteridaceae	*Pellaea* sp.	RLF-185				0		0		0		0		0		0		Iz	0	Ixc	W		
671	Ranunculaceae	*Anemone mexicana* Kunth	RLF-43, RLF-128, RLF-271, SRL-1240	Mariposa	2		0	-0.7655	0	-1.4404	0		0		0		0		BEA, BEM, BG, Pal, VR	0	Ixc	W	F	*in situ*
672	Ranunculaceae	*Clematis dioica* L.	SRL-303, SRL-1305				0		0		0		0		0		0		Me, Paz	0	Ixc	W		
673	Ranunculaceae	*Consolida ajacis* (L.) Schur	ERL-182	Conejito	2	Ceremonial = 14	0		0.0147		0		0		0		0		Sol	0	EAAA	W	E, P, Prp	*ex situ*
674	Ranunculaceae	*Delphinium bicornutum* Hemsl.	SRL-1200	Conejito	1	Ceremonial = 8	0		0		0		0		0	-1.5677	0		BEA	0	Ixc	W	G	*in situ*
675	Ranunculaceae	*Thalictrum gibbosum* Lecoy.	RLF-212, RLF-302	Chichicasle	1		0		0		0	-1.0487	0		0		0		Iz	0	Ixc	W	G	*in situ*
676	Rhamnaceae	*Condalia mexicana* Schltdl.	RLF-86, SRL-457, SRL-1147	Espino capulín	3	Ornamental = 29	0		0.0074	0.0446	0		0	-0.8476	0		0		Pal, Sol	0.000128	Ixc	W	G, T	*in situ*
677	Rhamnaceae	*Ziziphus amole* (Sessé & Moc.) M.C.Johnst.	SRL-1329	Cholulo	1		0		0		0		0		0		0		CaCe	0	Ixc	W	G	*in situ*
679	Rosaceae	*Cercocarpus fothergilloides* Kunth	SRL-1489	Ramoncillo	2		0		0		0		0		0		0		Me	0	Ixc	W	G	*in situ*
680	Rosaceae	*Crataegus mexicana* Moc. & Sess‚ ex DC	SRL-1424	Tejocote	1	Edible = 35	0		0		0		0.0108	-0.4619	0		0		Paz, TS	0	Ixc	D	G, Prp, T	*in situ*
681	Rosaceae	*Eriobotrya japonica* (Thunb.) Lindl.	SRL-50	Níspero	2	Ornamental = 47, edible = 15	0		0.0042		0		0.0082		0		0		Sol	0.000205	EAAA	D	E, P, Prp, Ti	*ex situ*
682	Rosaceae	*Lindleya mespiloides* Kunth	SRL-1223, SRL-1493	Hierba de pajarito, campanita grande	2		0		0		0	-0.8599	0		0.0147	-1.3761	0		Me, SB	0	Ixc	W	G	*in situ*
678	Rosaceae	*Malacomeles denticulata* (Kunth) G.N.Jones	RLF-10, RLF-243, SRL-261, SRL-338, SRL-474, SRL-1257, SRL-1258	Tlasisle	4		0.0121	1.5381	0		0	0.571	0	0.033	0		0		BEA, BEC, BN, Iz, Me, Palm, TS	0.045749	Ixc	W	F, G, T	*in situ*
686	Rosaceae	*Malus domestica* Borkh.	ERL-82, ERL-205, SRL-227	Manzana	2	Ornamental = 94, edible = 5	0		0		0		0.0150		0		0		Sol	0.000409	EAAA	D	P, Ti	*ex situ*
683	Rosaceae	*Prunus armeniaca* L.	ERL-51, ERL-198	Chabacano	1		0		0		0		0.0095		0		0		Sol	0.000153	EAAA	D	P, Prp, Ti	*ex situ*
684	Rosaceae	*Prunus persica* (L.) Batsch	SRL-226, ERL-199	Durazno	2	Ornamental = 82, edible = 25	0		0.0098		0		0.0129		0		0		Pal, Sol	0.000358	EAAA	D	P, Prp, Ti	*ex situ*
685	Rosaceae	*Prunus serotina* subsp. *capuli* (Cav. ex Spreng.) McVaugh	SRL-1412	Capulí	1		0		0		0		0.0029		0		0		Paz, TS	0	TCV	D	G, Prp, T	*ex situ*
687	Rosaceae	*Rosa* sp.	Photo record	Rosa	2	Ornamental = 59, ceremonial = 14	0		0.1298		0		0		0.0486		0		Sol	0.000256	EAAA	D	P, Prp, Ti	*ex situ*
688	Rosaceae	*Rosa* sp.	ERL-240	Rosa de ramito	2	Ornamental = 6	0		0.0165		0		0		0		0		Sol	0.000026	EAAA	D	P, Ti	*ex situ*
689	Rosaceae	*Xerospiraea hartwegiana* (Rydb.) Henrickson	SRL-1490				0		0		0		0		0		0		Me	0	Ixc	W		
690	Rubiaceae	*Bouvardia longiflora* (Cav.) Kunth	Photo record	Huele de noche	1		0		0		0		0		0.0058	-1.6222	0		Me	0	Ixc	W	G	*in situ*
691	Rubiaceae	*Bouvardia ternifolia* (Cav.) Schltdl.	RLF-41, RLF-166, SRL-262, SRL-334, SRL-1417	Ventorilla, flor de triste	4	Ceremonial = 8	0	0.4335	0.0294	0.2563	0	-0.1102	0		0	-0.5674	0		BEA, BEC, Me, Palm, Paz, TS	0.001181	Ixc	W	F, G, T, Ur	*in situ*
692	Rubiaceae	*Chiococca alba* (L.) Hitchc.	SRL-336, SRL-470, SRL-1111, SRL-1331, SRL-1441	Campanita	3	Ceremonial = 99	0		0.0294	1.2554	0		0		0.0661	1.7204	0	0.6818	CaCe, Me, Sol	0.000291	Ixc	W	G, P, Ti	*ex situ, in situ*
693	Rubiaceae	*Coutaportla ghiesbreghtiana* (Baill.) Urb.	SRL-406				0		0		0		0		0		0		Iz	0	Ixc	W		
694	Rubiaceae	*Crusea diversifolia* (Kunth) W.R.Anderson	RLF-21, RLF-111, SRL-381, SRL-1181				0		0		0		0		0		0		BEA, Me, Palm	0	Ixc	W		
695	Rubiaceae	*Crusea* sp.	RLF-136, SRL-1180		1		0		0		0		0		0	-1.6375	0		Me, Palm	0	Ixc	W	G	*in situ*
696	Rubiaceae	*Didymaea alsinoides* (Cham. & Schltdl.) Standl.	SRL-322				0		0		0		0		0		0		BEA, BEC	0.005571	Ixc	W		
697	Rubiaceae	*Galium* sp.	RLF-82, RLF-280, SRL-344		1		0		0		0	-0.9933	0		0		0		BEA, Me, Pal, Palm	0.003340	Ixc	W	G	*in situ*
698	Rubiaceae	*Randia capitata* DC.	RLF-281, SRL-1208	Limoncito de coyote	1		0		0		0	-1.0487	0		0		0		BEA, Pal, VR	0	Ixc	W	G	*in situ*
699	Rubiaceae	*Randia thurberi* S.Watson	SRL-1344				0		0		0		0		0		0		CaCe	0	Ixc	W		
700	Rutaceae	*Casimiroa edulis* La Llave	ERL-130, ERL-176	Zapote blanco	4	Edible = 5	0		0		0.021		0.0132		0		0.0095		Sol	0.000153	TCV	D	E, P, Prp, T	*ex situ*
701	Rutaceae	*Citrus aurantiifolia* (Christm.) Swingle	Photo record	Limón	3	Ornamental = 71, medicinal = 5, edible = 100	0		0.0189		0.0046		0.0124		0		0		Sol	0.000307	EAAA	D	E, P, T, Ti	*ex situ*
704	Rutaceae	*Citrus maxima* (Burm.) Merr.	Photo record	Toronja	1	Ornamental = 6	0		0.0147		0		0		0		0		Sol	0.000026	EAAA	D	P, Ti	*ex situ*
703	Rutaceae	*Citrus sinensis* (L.) Osbeck	Photo record	Naranja	4	Ornamental = 12, edible = 100	0		0.0147		0.0015		0		0		0		Sol	0.000051	EAAA	D	P, T, Ti	*ex situ*
705	Rutaceae	*Citrus reticulata* Blanco	Photo record	Mandarina	1		0		0		0		0		0		0		Sol	0.000051	EAAA	D	P, T, Ti	*ex situ*
702	Rutaceae	*Citrus* × *latifolia* (Yu.Tanaka) Yu.Tanaka	Photo record	Lima	2		0		0		0.0056		0		0		0		Sol	0.000026	EAAA	D	P, Ti	*ex situ*
706	Rutaceae	*Ptelea trifoliata* L.	ERL-196, RLF-27, RLF-308, SRL-274, SRL-466, SRL-467	Hierba de zorrillo	3	Firewood = 100	0		0		0.0028	-0.2649	0		0		0.0071	-0.7535	BEA, BEC, BG, BN, Iz, Me, Palm, Sol, TS	0.007574	Ixc	W	G, T	*in situ*
707	Rutaceae	*Ruta chalepensis* L.	ERL-93, ERL-127, ERL-208, ERL-241, SRL-68	Ruda	2	Ornamental = 53	0		0		0.0427		0		0		0		Sol	0.000230	EAAA	W	P, Prp	*ex situ*
708	Rutaceae	*Zanthoxylum* sp.	SRL-1221				0		0		0		0		0		0		Iz, SB	0.000678	Ixc	W		
709	Rutaceae	*Zanthoxylum* sp.	SRL-326	Hierba de zorrillo	1		0		0		0		0		0		0	-1.932	BEA	0	Ixc	W	G	*in situ*
710	Rutaceae	*Zanthoxylum* sp.	SRL-1348		1		0		0		0	-1.0487	0		0		0		CaCe	0	Ixc	W	G	*in situ*
394	Salicaceae	*Neopringlea viscosa* (Liebm.) Rose	SRL.337				0		0		0		0		0		0		Me	0.000118	Ixc	W		
711	Salicaceae	*Salix bonplandiana* Kunth	SRL-204	Sauce	3		0		0		0		0		0		0		Pal	0	TCV	W	Ti	*ex situ*
779	Santalaceae	*Phoradendron reichenbachianum* (Seem.) Oliv.	RLF-329, SRL-1483	Injerto			0		0		0		0		0		0		CaMy, BE, Iz	0	Ixc	W	Ur	*in situ*
780	Santalaceae	*Phoradendron* sp.	ERL-180, SRL-1558	Injerto, chahuistle			0		0		0		0		0		0		Me, Sol	0.000051	Ixc	W	Ur	*in situ*
781	Santalaceae	*Phoradendron* sp.	RLF-228, SRL-1268	Injerto	2		0	-0.298	0		0	-0.5662	0		0		0		Iz, Me	0	Ixc	W	G, Ur	*in situ*
712	Sapindaceae	*Dodonaea viscosa* (L.) Jacq.	RLF-30, SRL-294, SRL-473, SRL-1118, ERL-189	Cachovenado	4	Firewood = 100	0		0.0147	0.2881	0		0		0		0.0549	-0.2043	BEA, BEC, BN, Iz, Me, Palm, Sol, TS	0.021155	Ixc	W	G, T	*in situ*
713	Sapindaceae	*Urvillea ulmacea* Kunth	SRL-1332				0		0		0		0		0		0		SB	0	Ixc	W		
715	Sapotaceae	*Sideroxylon palmeri* (Rose) T.D.Penn.	ERL-219, SRL-454	Tempesquistle	1	Edible = 90	0		0		0		0.0132		0		0		Sol	0.000051	TCV	D	P, Prp, T	*ex situ*
716	Sapotaceae	*Sideroxylon salicifolium* (L.) Lam.	RLF-244	Tempesquitle cimarrón, laurelillo	1		0	-1.0765	0		0		0		0		0		Iz	0	Ixc	W	F	*in situ*
714	Sapotaceae	*Sideroxylon capiri* (A.DC.) Pittier	SRL-1508				0		0		0		0		0		0		AA	0	Ixc	W		
730	Selaginellaceae	*Selaginella lepidophylla* (Hook. & Grev.) Spring	SRL-374, SRL-1497		1		0		0		0	-1.0487	0		0		0		BEA, Me	0	Ixc	W	G	*in situ*
731	Simaroubaceae	*Castela erecta* Turpin	SRL-1382				0		0		0		0		0		0		SB	0	Ixc	W		
732	Smilacaceae	*Smilax moranensis* M.Martens & Galeottii	SRL-233				0		0		0		0		0		0		BEA	0	Ixc	W		
733	Solanaceae	*Brugmansia × candida* Pers.	SRL-63	Floribundio	2	Ornamental = 12, ceremonial = 17	0		0		0		0		0.0139		0		Sol	0.000051	AC	D	P, Prp	*ex situ*
734	Solanaceae	*Capsicum annuum* L.	ERL-165, ERL-204	Chilar de arbolito, caquita de ratón, chiltepe, cuaresmeño, guajillo, piquín, verde	3	Edible = 100	0		0		0		0.0065	4.5368	0		0		SB, Sol	0.000153	Ixc	D, W	E, G, P, Prp, T, Ti	*ex situ, in situ*
735	Solanaceae	*Capsicum pubescens* Ruiz & Pav.	ERL-181	Chile canario	2		0		0		0		0.0020		0		0		Sol	0.000077	AC	D	E, P, Prp, T, Ti	*ex situ*
736	Solanaceae	*Capsicum* sp.	RLF-135		1		0		0		0	-1.0487	0		0		0		Me	0	Ixc	W	G	*in situ*
737	Solanaceae	*Capsicum* sp.	SRL-165		1		0	-1.0765	0		0		0		0		0		BEA, Pal, VR	0	Ixc	W	F	*in situ*
738	Solanaceae	*Datura stramonuim* L.	SRL-1284				0		0		0		0		0		0		Pal	0	Ixc	R-W, W	T	*in situ*
739	Solanaceae	*Jaltomata procumbens* (Cav.) J.L.Gentry	SRL-180, SRL-1297	Hierba mora	2		0		0		0	-0.6249	0	-1.0133	0		0		Palm, Sol	0	Ixc	R-W, W	G, T	*in situ*
740	Solanaceae	*Lycianthes ciliolata* (M.Martens & Galeotti) Bitter	SRL-1149	Ojo de toro	2		0		0		0.0051	-0.5422	0.0020	-0.9978	0		0		BEA, BG, Pal, Palm, Sol	0	Ixc	R-W, W	G, T	*in situ*
741	Solanaceae	*Nicotiana glauca* Graham	ERL-37, RLF-105, SRL-171, SRL-1274	Gigante	4	Ornamental = 6, firewood = 100	0		0		0.0028		0		0		0.0069		Bal, Pal, Sol, TS	0.000026	Nat-AC	R-W	G, T	*ex situ*
742	Solanaceae	*Nicotiana tabacum* L.	SRL-240	Tabaco	1		0		0		0		0		0		0		Sol	0.000077	Mex	D	G, T	*ex situ*
743	Solanaceae	*Physalis philadelphica* Lam.	ERL-36, ERL-63, ERL-64, ERL-113, RLF-312, SRL-26, SRL-1138, SRL-1298	Miltomate, tomate, tomate de milpa	2	Edible = 100	0		0		0.0069	1.5091	0.0150	0.9152	0		0		Sol, Ts	0.001383	Ixc	D, R-W	E, P, Prp, T, Ti	*in situ*
744	Solanaceae	*Solandra maxima* (Moc. & Sessé ex Dunal) P.S.Green	Photo record	Copa de oro	1		0		0.0059		0		0		0		0		Sol	0	AC	D	P, Ti	*ex situ*
745	Solanaceae	*Solanum americanum* Mill.	SRL-1234	Ticungo	1		0		0		0		0	-1.1768	0		0		Sol	0.000026	Ixc	R-W	G, T	*in situ*
746	Solanaceae	*Solanum erianthum* D.Don.	ERL-91	Tepozán	1		0		0		0.0046	-0.7375	0		0		0		Sol	0.000026	Ixc	R-W, W	G, T	*in situ*
747	Solanaceae	*Solanum lanceolatum* Cav	ERL-195	Tepozán	1		0		0		0.0046	-0.6538	0		0		0		BEA, BEC, BG, Palm, Sol	0.005064	Ixc	R-W, W	G, T	*in situ*
748	Solanaceae	*Solanum lesteri* Hawkes & Hjert.	RLF-151	Hierba del tomate pinto	1		0		0		0		0	-1.3855	0		0		Paz	0	Ixc	W	G	*in situ*
749	Solanaceae	*Solanum lycopersicum* L.	Photo record	Jitomate	1	Edible = 100	0		0		0		0.0128		0		0		Pal, Sol	0.000205	TCV	D	E, P, Prp, T, Ti	*ex situ*
750	Solanaceae	*Solanum rostratum* Dunal	SRL-380	Chicalote de burro	1		0		0		0	-1.0487	0		0		0		BEA	0	Ixc	R-W	G	*in situ*
751	Solanaceae	*Solanum rudepannum* Dunal	RLF-22, RLF-95, RLF-120, RLF-275, SRL-128, SRL-302	Tepozán	2		0		0		0.0046	-0.784	0	-1.2217	0		0		Sol, BEA, BEC, Me, Pal, Paz	0	Ixc	R-W, W	G	*in situ*
753	Solanaceae	*Solanum tridynamum* Dunal	SRL-1361, SRL-1391				0		0		0		0		0		0		CaCe	0	Ixc	R-W		
754	Solanaceae	*Solanum tuberosum* L.	Photo record	Papa	1	Edible = 100	0		0		0		0.0044		0		0		Sol, TS	0	AC	D	P, Prp	*ex situ*
752	Solanaceae	*Solanum* sp.	SRL-27				0		0		0		0		0		0		Bal	0	Ixc	R-W	T	*in situ*
756	Sterculiaceae	*Melochia* sp.	SRL-1555				0		0		0		0		0		0		CaCe	0	Ixc	W		
659	Talinaceae	*Talinum* sp.	SRL-414		1		0	-1.023	0		0		0		0		0		BG	0.001995	Ixc	W	F	*in situ*
757	Thelypteridaceae	*Thelypteris albicaulis* (Fée) A.R.Sm.	SRL-200	Pojalillo			0		0		0		0		0		0		Palm	0	Ixc	W		
758	Thelypteridaceae	*Thelypteris* sp.	SRL-161, RLF-303		1		0		0		0		0		0	-1.6245	0		BEA, Iz, Pal	0.000658	Ixc	W	G	*in situ*
760	Tropaeolaceae	*Tropaeolum majus* L.	ERL-18, ERL-89, RLF-182, SRL-60, SRL-196	Mastuerzo	3	Ornamental = 18	0		0.0033		0		0		0.0056		0		Sol	0.000077	Nat-AC	R-W	P, Prp, T	*ex situ*
762	Typhaceae	*Typha* sp.	Photo record				0		0		0		0		0		0		VR	0	Ixc	W		
764	Urticaceae	*Parietaria pensylvanica* Muhl. ex Willd.	ERL-73, RLF-88, RLF-266, SRL-18	Paletaria	1		0		0		0.0159	-0.5533	0		0		0		BEA, Pal, Sol, VR	0.000026	Ixc	W	G, T	*in situ*
765	Urticaceae	*Pilea microphylla* (L.) Liebm.	RLF-171, SRL-1256, SRL-1309	Pinolillo	1	Ornamental = 6	0		0	0.0738	0		0		0		0		BEA, Me, Sol	0.000026	Ixc	W	P, Prp	*ex situ, in situ*
766	Urticaceae	*Urera caracasana* (Jacq.) Gaudich. ex Griseb.	SRL-1543	Chichicasle	2		0		0		0.0031	-0.5744	0		0		0		Sol	0	Ixc	W	G, T	*in situ*
768	Verbenaceae	*Citharexylum aff. bourgeauianum* Greenm.	SRL-1215		1		0		0	-2.1063	0		0		0		0		SB	0	Ixc	W		
769	Verbenaceae	*Citharexylum tetramerum* Brandegee	Photo record				0		0		0		0		0		0		Palm	0	Ixc	W		
770	Verbenaceae	*Glandularia elegans* (Kunth) Umber	RLF-5, SRL-110, SRL-279, SRL-1326, SRL-1479		1		0		0		0	-1.0167	0		0		0		Bal, BEA, BN, Sol	0.001928	Ixc	R-W	G	*in situ*
771	Verbenaceae	*Lantana achyranthifolia* Desf.	RLF-61, RLF-62, SRL-109, SRL-152, SRL-369, SRL-1296	Hierba buena de monte	2		0	-0.2001	0		0	-0.4950	0		0		0		BEA, BN, Me, Pal, Palm	0.000747	Ixc	R-W, W	F, G, T, Ur	*in situ*
772	Verbenaceae	*Lantana camara* L.	RLF-91, RLF-197, SRL-115, SRL-459, SRL-1112, SRL-1154, SRL-1169, SRL-1365	Tiundica, siete negritos	4		0.0054	3.3596	0	0.8495	0.0056	2.2797	0	1.3843	0		0		BEA, BEC, BN, CaCe, Iz, Me, Palm, Sol	0.003620	Ixc	R-W, W	F, G, P, Ti	*ex situ, in situ*
773	Verbenaceae	*Lantana velutina* M.Martens & Galeotti	ERL-185, RLF-31, RLF-204, SRL-272, SRL-1115, SRL-1168	Tiundica blanca, cinco negritos	4	Ornamental = 12	0		0	1.484	0	2.4772	0	1.6392	0		0		BEA, BEC, BN, Iz, Me, Pal, Palm, Sol	0.010387	Ixc	R-W, W	G, T, Ti	*ex situ, in situ*
774	Verbenaceae	*Lippia graveolen* Kunth		Oreganillo, salvarreal de castilla	4	Medicinal = 5	0.0065	0.0052	0		0.0069	0.0526	0	-0.8419	0		0		CaCe, Me, Pal	0	Ixc	W	F, G	*in situ*
775	Verbenaceae	*Lippia oaxacana* B.L.Rob. & Greenm.	SRL-71, SRL-1378, SRL-1454, SRL-1549	Salvarreal	2	Medicinal = 60	0		0		0.2636	10.3582	0.0066	1.002	0		0		Me, Sol	0	Ixc	W	G, P, Ti	*ex situ, in situ*
776	Verbenaceae	*Priva mexicana* (L.) Pers.	RLF-29	Piojito			0		0		0		0		0		0		BEA	0	Ixc	R-W		
777	Verbenaceae	*Stachytarpheta acuminata* A.DC.	SRL-1380				0		0		0		0		0		0		SB	0	Ixc	R-W		
778	Verbenaceae	*Verbena carolina* L.	RLF-93, SRL-125, SRL-173, SRL-456		1		0		0	-1.5594	0		0		0		0		BEA, Sol	0	Ixc	R-W	T	*in situ*
782	Vitaceae	*Cissus* sp.	RLF-101, RLF-173, SRL-1373, SRL-1535	Tripa de diablo	2		0		0	-1.2488	0	-0.6837	0		0		0		CaCe, Sol, TS	0	Ixc	R-W	T, Ur	*in situ*
783	Vitaceae	*Vitis vinifera* L.	SRL-54	Uva	2		0		0		0		0		0		0		Sol	0	EAAA	D	P, Ti	*ex situ*
27	Xanthorrhoeaceae	*Aloe vera* (L.) Burm.f.	ERL-188, SRL-78	Sábila	5	Ornamental = 47	0		0		0.0552		0		0		0		Sol	0.000205	EAAA	D, R-W	P, Prp, Ti	*ex situ*
82	Xanthorrhoeaceae	*Asphodelus fistulosus* L.	SRL-388, SRL-1415		1	Ornamental = 6	0		0		0		0		0		0		BEM, Pz, Sol	0.000026	Nat-EAAA	W	P, T, Ti, Ur	*ex situ*
83	Xanthorrhoeaceae	*Kniphofia uvaria* (L.) Oken	ERL-158	Bandera española	2	Ornamental = 24	0		0		0		0		0		0		Sol	0.000102	EAAA	W	P, Prp	*ex situ*
784	Zygophyllaceae	*Morkillia mexicana* (DC.) Rose & Painter	SRL-1338, SRL-1349				0		0		0		0		0		0		CaCe	0	Ixc	W		
785				Octavillo	1	Ceremonial = 17	0		0		0		0		0.025	-1.433	0		BEM	0	Ixc	W	G	*in situ*

Notes

^a^ Collectors name: *ERL* Erandi Rivera Lozoya, *RLF* Ricardo Lemus Fernández, *RJS* José Rosario Jiménez Salazar, *SRL* Selene Rangel Landa

^b^ Fodder plants Sutrop Index details: Number of lists = 31; Average length of lists = 6; Number of cited items = 65; Total number of cited items = 195; Number of collected lists for no new information addition = 14. Sutrop Index rarefaction curve 1

^c^ Ornamental plants Sutrop Index details: Number of lists = 34; Average length of lists = 6; Number of cited items = 85; Total number of cited items = 200; Number of collected lists for no new information addition = 25. Sutrop Index rarefaction curve 2

^d^ Medicinal plants Sutrop Index details: Number of lists = 36; Average lengthof lists = 8; Number of cited items =76; Total number of cited items = 285; Number of collected lists for no new information addition = 19. Sutrop Index rarefaction curve 3

^e^ Edible plants Sutrop Index details: Number of lists = 38; Average length of lists = 10; Number of cited items =83; Total number of cited items = 387; Number of collected lists for no new information addition = 19. Sutrop Index rarefaction curve 4

^f^ Ceremonial plants Sutrop Index details: Number of lists = 36; Average length of lists = 5; Number of cited items =41; Total number of cited items = 185; Number of collected lists for no new information addition = 13. Sutrop Index rarefaction curve 5

^g^ Firewood Sutrop Index details: Number of lists = 35; Average length of lists = 7; Number of cited items =39; Total number of cited items = 244; Number of collected lists for no new information addition = 9. Sutrop Index rarefaction curve 6

^h^ Key to vegetation type: *AA* Anicent settlements, *Bal* Urban secondary vegetation, *BEA* Quercus liebmanni and Quercus laeta forest, *BEC* Quercus urbanni forest, *BEM* Quercus spp.forest, *BG* Gallery forest (Taxodium mucronatum), *BN* Juniperus flaccida forest, *CaCe* Cephalocereus colummna-trajanni shrubland, *CaMy* Pseudomytrocereus fulviceps shrubland, *Iz* Izotal (shrubland dominated by rosettes), *Me* Mexical, *Pal* Mescal factories, *Palm* Palm shrubland of Brahea dulcis, *Paz* grassland, *SB* Tropical dry forest, *Sol* Homegardens, *TS* Agricultural fields, *VR* Riparian vegetation

^i^ Key to Area of Origin: *AC* American Continent, *EAAA* Europa, Asia, Africa, Australia, *Ixc* Ixcatlán (species with wild populations in Ixcatlán territory, and Mesoamerican area native species that have naturalized populations in Ixcatlán territory), *Mex* Mexico, *TCV* Tehuacán-Cuicatlán Valley (plants natives of VTC but in Ixcatlan only could be finding in settlements under cultivation), *Uk* Unknown

^j^: Key to Ecological Status: *D* Domesticated, *R-W* Ruderal-Weedy, *W* Wild

^k^: Key to Management practices: *E* Enhancement, *F* Forage, *G* Gathering, *P* Protection, *Prp* Propagation, *T* Tolerance, *Ti* Transplanting of individuals, *Ur* Uproot

^l^: Key to Management site: In situ = when management take place in sites where species wild populations are distributed; ex situ = when management take place in sites out of species wild populations distribution

### Names of Botanical experts who contributed to determine the voucher specimens

Aarón Rodríguez Contreras, Abisaí Josué García-Mendoza, Alfonso Valiente-Banuet, Ana Rosa Lopez Ferrari, Mario Adolfo Espejo Serna, Anna Paizanni Guillén, Claudio Delgadillo Moya, Darisol Pacheco Rivera, Eduardo Ruíz-Sánchez, Emmanuel Pérez Calix, Ernesto Velázquez, Montes, Gerardo A. Salazar Chávez, Guadalupe Cornejo-Tenorio, Guillermo Ibarra Manríquez, José Luis Villaseñor Ríos, Juan Ismael Calzada, Luz María González-Villarreal, María de los Angeles Herrera, Mauricio Antonio Mora Jarvio, Oswaldo Tellez Valdés, Pablo Carrillo-Reyes, Rafael Lira Saade, Rosario Redonda-Martínez, Rosalinda Medina-Lemos, Salvador Arias, Sergio Zamudio Ruíz, Susana Valencia Ávalos, Verónica Juárez-Jaimes, Victor W. Steinmann

## References

[1] Toledo VM, Ortiz-Espejel B, Cortés L, Moguel P, de Ordonez M (2003). The multiple use of tropical forests by indigenous peoples in Mexico: a case of adaptive management. Conserv. Ecol..

[2] Berkes F, Folke C (1998). Linking social and ecological systems: managment practices and social mechanisms for building resilience.

[3] Boege E (2008). El patrimonio biocultural de los pueblos indígenas de México.

[4] Toledo VM, Barrera-Bassols N (2008). La Memoria Biocultural: la importancia ecológica de las sabidurias tradicionales.

[5] Toledo VM, Boege E, Barrera-Bassols N (2010). The biocultural heritage of Mexico: an overview. Langscape..

[6] Casas A, Camou-Guerrero A, Otero-Arnaiz A, Rangel-Landa S, Cruse-Sanders J, Solís L (2014). Manejo tradicional de biodiversidad y ecosistemas en Mesoamérica: el Valle de Tehuacán. Investig. Ambient. Cienc. y política pública..

[7] Berkes F, Colding J, Folke C (2000). Rediscovery of traditional ecological knowledge as adaptive management. Ecol. Appl..

[8] Moreno-Calles AI, Toledo VM, Casas A (2013). Los sistemas agroforestales tradicionales de México: una aproximación biocultural. Bot. Sci..

[9] Pretty J, Adams B, Berkes F, de Athayde SF, Dudley N, Hunn E (2009). The intersections of biological diversity and cultural diversity: Towards. Conserv. Soc..

[10] Casas A, Otero-Arnaiz A, Pérez-Negrón E, Valiente-Banuet A (2007). In situ management and domestication of plants in Mesoamerica. Ann. Bot..

[11] Blancas J, Casas A, Rangel-Landa S, Moreno-Calles A, Torres I, Pérez-Negrón E (2010). Plant management in the Tehuacan-Cuicatlan Valley, Mexico. Econ. Bot..

[12] El OE (2011). gobierno de los bienes comunes. La evolución de las instituciones de acción colectiva. 2nd ed.

[13] Toledo VM, Steep JR (2002). Etnoecology: A conceptual framework for the study of indigenous knowledge of nature. Ethnobiology and cultural diversity.

[14] González-Insuasti MS, Martorell C, Caballero J (2008). Factors that influence the intensity of non-agricultural management of plant resources. Agrofor. Syst..

[15] Blancas J, Casas A, Pérez-Salicrup D, Caballero J, Vega E (2013). Ecological and socio-cultural factors influencing plant management in Náhuatl communities of the Tehuacán Valley, Mexico. J Ethnobiol. Ethnomed..

[16] Arellanes Y, Casas A, Arellanes A, Vega E, Blancas J, Vallejo M (2013). Influence of traditional markets on plant management in the Tehuacán Valley. J. Ethnobiol. Ethnomed..

[17] Blancas J, Pérez-Salicrup D, Casas A (2014). Evaluando la incertidumbre en la disponibilidad de recursos vegetales. Gaia Sci..

[18] Phillips O, Gentry AH (1993). The useful plants of Tambopata, Peru: II. Additional hypothesis testing in quantitative ethnobotany. Econ Bot.

[19] Albuquerque UP, Soldati GT, Ramos MA, Melo JG, Medeiros PM, Nascimento ALB, Albuquerque UP, Medeiros PM, Casas A (2015). The influence of the environment on natural resource use: evidence of apparency. Evolutionary ethnobiology.

[20] Zent EL (2013). Jotï ecogony, Venezuelan Amazon. Environ. Res. Lett..

[21] Moreno-Calles A, Casas A, Blancas J, Torres I, Masera O (2010). Javier Caballero, et al. Agroforestry systems and biodiversity conservation in arid zones: the case of the Tehuacán Valley, Central México. Agrofor. Syst.

[22] Vallejo M, Casas A, Blancas J, Moreno-Calles AI, Solís L, Rangel-Landa S (2014). Agroforestry systems in the highlands of the Tehuacán Valley, Mexico: indigenous cultures and biodiversity conservation. Agrofor. Syst..

[23] MacNeish R (1992). The origins of agriculture and setteled life.

[24] Dávila P, Arizmendi M del C, Valiente-Banuet A, Villaseñor JL, Casas A, Lira R (2002). Biological diversity in the Tehuacán-Cuicatlán Valley, Mexico. Biodivers. Conserv.

[25] Lira R, Casas A, Rosas-López R, Paredes-Flores M, Pérez-Negrón E, Rangel-Landa S (2009). Traditional knowledge and useful plant richness in the Tehuacán–Cuicatlán Valley, Mexico. Econ. Bot..

[26] Diario Oficial de la Federación (1948). Resolución sobre conflicto por límites de bienes comunales al poblado de Santa María Ixcatlán, municipio del mismo nombre, Estado de Oaxaca.

[27] Hironymous MO (2007). Santa María Ixcatlan, Oaxaca: From colonial cacicazgo to modern municipio.

[28] Servicio Meteorológico Nacional (2010). Normales climatológicas 1951-2010: Estación 00020129 Santa María Ixcatlán, Oaxaca.

[29] García E, García EC (2008). Climas, Catálogo de metadatos geográficos 1:1000000.

[30] INEGI (2013). Conjunto de datos vectoriales de la carta de uso del suelo y vegetación 1:250,000, serie V (Conjunto Nacional).

[31] INEGI (2010). México en cifras: Santa María Ixcatlán, Oaxaca.

[32] Nava C, Romero M (2007). Ixcatecos, pueblos indígenas del México contemporáneo.

[33] Cook SF (1958). Santa María Ixcatlán: habitat, population, subsistence. Ibero-Amer.

[34] Lewis MP, Simons GF, Fennig CD, Paul LM, Simons GF, Fennig CD (2016). Ethnologue: Languages of the World.

[35] Swanton M, Van Doesburg S (2008). La escritura indígena como “material lingüístico”. Una carta en lengua ixcateca al presidente Lázaro Cárdenas. Pictografía y escritura alfabética en Oaxaca.

[36] Valiente-Banuet A, Solís L, Dávila P, del Arizmendi M, Arizmendi Mdel C, Silva C, Ortega-Ramírez J (2009). Guía de la vegetación del Valle de Tehuacán-Cuicatlán.

[37] The Plant List Version 1.1. Published on the Internet. 2013. http://www.theplantlist.org/. Accessed on Mar 2016.

[38] Martin G (2004). Ethnobotany a methods manual.

[39] Pérez-Negrón E, Casas A (2007). Use, extraction rates and spatial availability of plant resources in the Tehuacán-Cuicatlán Valley, Mexico: The case of Santiago Quiotepec, Oaxaca. J. Arid Environ..

[40] Delgado-Lemus A, Torres I, Blancas J, Casas A (2014). Vulnerability and risk management of Agave species in the Tehuacán Valley, México. J. Ethnobiol. Ethnomed..

[41] Husson F, Lê S, Pagès J (2011). Exploratory multivariate analysis by example using R.

[42] SAS-Institute-INC. JMP 8.0. 2008. http://www.jmp.com

[43] Sutrop U (2001). List task and a cognitive salience index. Field Methods..

[44] Pennec F, Wencelius J, Garine E, Raimond C, Bohbot H (2012). FLAME v1.0: Free-list analysis under Microsoft Excel.

[45] Curtis JT (1959). The vegetation of Wisconsin, an ordination of plant communities.

[46] Casas A, Viveros JL, Caballero J (1994). Etnobotánica mixteca: sociedad, cultura y recursos naturales en la Montaña de Guerrero.

[47] Farfán B, Casas A, Ibarra-Manríquez G, Pérez-Negrón E (2007). Mazahua ethnobotany and subsistence in the Monarch Butterfly Biosphere Reserve, Mexico. Econ. Bot..

[48] Alcorn JB (1984). Huastec Mayan Ethnobotany.

[49] Hunn ES (2008). A Zapotec natural history.

[50] Moreno-Calles AI, Casas A, García-Frapolli E, Torres-García I (2012). Traditional agroforestry systems of multi-crop “milpa” and “chichipera” cactus forest in the arid Tehuacán Valley, Mexico: their management and role in people’s subsistence. Agrofor. Syst..

[51] Velázquez DeLara G, Acuña R (1984). Relación de Ixcatlán, Quiotepec y Tecomahuaca. Relaciones Geográficas del siglo XVI: Antequera.

[52] Rangel-Landa S, Rivera-Lozoya E, Casas A (2014). Uso y manejo de las palmas Brahea spp. (Arecaceae) por el pueblo ixcateco de Santa María Ixcatlán Oaxaca, México. Gaia Sci.

[53] García-Frapolli E, Toledo VM, Martínez-Alier J (2008). Apropiación de la naturaleza por una comunidad Maya Yucateca: Un análisis económico-ecológico. Rev. Iberoaméricana Econ. Ecológica..

[54] Toledo VM, Barrera-Bassols N, García-Frapolli E, Alarcón-Cháires P (2008). Uso múltiple y biodiversidad entre los mayas yucatecos (México). Interciencia..

[55] Belcher B, Ruíz-Pérez M, Achdiawan R (2005). Global patterns and trends in the use and management of commercial NTFPs: Implications for livelihoods and conservation. World Dev..

[56] Casas A, Valiente-Banuet A, Viveros JL, Caballero J, Cortés L, Dávila P (2001). Plant resources of the Tehuacán-Cuicatlán Valley, Mexico. Econ. Bot..

[57] Casas A, Rangel-Landa S, Torres I, Pérez-Negrón E, Solís L, Parra F, de Albuquerque UP, Alves M (2008). In situ management and conservation of plant resources in the Tehuacan-Cuicatlan Valley, Mexico: an ethnobotanical and ecological perspective. Current topics in Ethnobotany.

[58] Casas A, Blancas J, Otero-Arnaiz A, Cruse-Sanders J, Lira R, Avendaño A, Lira R, Casas A, Blancas J (2016). Evolutionary ethnobotanical studies of incipient domestication of plants in Mesoamerica. Ethnobotany of Mexico: interactions of people and plants in Mesoamerica.

[59] Halstead P, O´Shea J (1989). Bad year economics: cultural responses to risk and uncertainty.

[60] Parlee B, Berkes F (2006). Indigenous knowledge of ecological variability and commons management: a case study on berry harvesting from Northern Canada. Hum. Ecol..

[61] Casas A, Parra F, Rangel S, Guillén S, Blancas J, Figueredo CJ, Albuquerque UP, Medeiros PM, Casas A (2015). Evolutionary ecology and ethnobiology. Evolutionary ethnobiology.

[62] Atangana A, Khasa D, Chang S, Degrande A (2014). Tropical agroforestry.

[63] Nair PKR (1985). Classification of agroforestry systems. Agrofor. Syst..

[64] Moreno-Calles AI, Galicia-Luna VJ, Casas A, Toledo VM, Ramos MV, Santos-Fita D (2014). La Etnoagroforestería: el estudio de los sistemas agroforestales tradicionales de México. Etnobiología..

[65] Casas A, Parra F, Blancas J, Albuquerque UP, Medeiros PM, Casas A (2015). Evolution of humans and by humans. Evolutionary ethnobiology.

[66] Mendoza E (1998). Los eternos tejedores de Santa María Ixcatlán. México Desconoc..

[67] Bartolomé M (1991). Historia Ixcateca. México: Instituto Nacional de Antropología e Historia, CIESAS unidad Oaxaca and Gobierno del Estado de Oaxaca.

[68] González-Insuasti MS, Caballero J (2007). Managing plant resources: How intensive can it be?. Hum. Ecol..

[69] Paredes M, Lira R, Dávila P (2007). Estudio etnobotánico de Zapotitlán Salinas, Puebla. Acta Botánica Mex..

[70] Larios C, Casas A, Vallejo M, Moreno-Calles AI, Blancas J (2013). Plant management and biodiversity conservation in Náhuatl homegardens of the Tehuacán Valley, Mexico. J. Ethnobiol. Ethnomed..

[71] Blanckaert I, Swennen R, Paredes-Flores M, Rosas López R, Lira R (2004). Floristic composition, plant uses and management practices in homegardens of San Rafael Coxcatlán, Valley of Tehuacán-Cuicatlán, Mexico. J. Arid Environ..

[72] Castaneda H, Stepp JR (2007). Ethnoecological importance value (EIV) methodology: Assessing the cultural importance of ecosystems as sources of useful plants for the Guaymi people of Costa Rica [Internet]. Ethnobot. Res. Appl..

[73] Challenger A, Bocco G, Equihua M, Chavero EL, Maass M (2014). La aplicación del concepto del sistema socio-ecológico: alcances, posibilidades y limitaciones en la gestión ambiental de México. Investig. Ambient. Cienc. y política pública..

[74] Lucena RFP, Medeiros PM, de Araújo L E, Alves AGC, Albuquerque UP (2012). The ecological apparency hypothesis and the importance of useful plants in rural communities from northeastern Brazil: an assessment based on use value. J. Environ. Manage.

[75] Maldonado B, Caballero J, Delgado-Salinas A, Lira R (2013). Relationship between use value and ecological importance of floristic resources of seasonally dry tropical forest in the Balsas river basin, Mexico. Econ. Bot..

[76] Medeiros PM, Ladio AH, Albuquerque UP, Albuquerque UP, Medeiros PM, Casas A (2015). Local criteria for medicinal plant selection. Evolutionary ethnobiology.

[77] Camou-Guerrero A. Los recursos vegetales en una comunidad rarámuri: aspectos culturales, económicos y ecológicos, Ph.D. thesis. Mexico: Universidad Nacional Autónoma de México; 2008.

[78] González-Insuasti MS, Casas A, Méndez-Ramírez I, Martorell C, Caballero J (2011). Intra-cultural differences in the importance of plant resources and their impact on management intensification in the Tehuacán Valley, Mexico. Hum. Ecol..

[79] Torres I, Blancas J, León A, Casas A (2015). TEK, local perceptions of risk, and diversity of management practices of Agave inaequidens in Michoacán, Mexico. J. Ethnobiol. Ethnomed..

[80] Berkes F, Turner NJ (2006). Knowledge, learning and the evolution of conservation practice for social-ecological. Hum. Ecol..

[81] Berkes F (2007). Understanding uncertainty and reducing vulnerability: lessons from resilience thinking. Nat. Hazards..

[82] Espinosa-García FJ, Díaz-Pérez R (1996). El uso campesino de plantas arvenses como forraje en el Valle de México. Etnoecológica..

[83] Bhagwat S, Willis KJ, Birks HJB, Whittaker RJ (2008). Agroforestry: a refuge for tropical biodiversity?. Trends Ecol. Evol.

[84] García LE. Aspectos socio-ecológicos para el manejo sustentable del copal en el Ejido de Acateyahualco, Gro, Bachelor thesis. Licenciatura en Ciencias Ambientales, México: Universidad Nacional Autónoma de México; 2012.

[85] Fowler CS, Lepofsky D, Anderson EN, Pearsall DM, Hunn ES, Turner NJ (2011). Traditional resource and environmental management. Ethnobiology.

